# Statistical analysis of synonymous and stop codons in pseudo-random and real sequences as a function of GC content

**DOI:** 10.1038/s41598-023-49626-9

**Published:** 2023-12-27

**Authors:** Valentin Wesp, Günter Theißen, Stefan Schuster

**Affiliations:** 1 Department of Bioinformatics, Matthias Schleiden Institute, Friedrich Schiller University Jena, Ernst-Abbe-Platz 2, 07743 Jena, Germany; 2 Department of Genetics, Matthias Schleiden Institute, Friedrich Schiller University Jena, Philosophenweg 12, 07743 Jena, Germany

**Keywords:** Computational models, Data mining, Genome informatics, Statistical methods, Eukaryote, Genome, Prokaryote

## Abstract

Knowledge of the frequencies of synonymous triplets in protein-coding and non-coding DNA stretches can be used in gene finding. These frequencies depend on the GC content of the genome or parts of it. An example of interest is provided by stop codons. This is relevant for the definition of Open Reading Frames. A generic case is provided by pseudo-random sequences, especially when they code for complex proteins or when they are non-coding and not subject to selection pressure. Here, we calculate, for such sequences and for all 25 known genetic codes, the frequency of each amino acid and stop codon based on their set of codons and as a function of GC content. The amino acids can be classified into five groups according to the GC content where their expected frequency reaches its maximum. We determine the overall Shannon information based on groups of synonymous codons and show that it becomes maximum at a percent GC of 43.3% (for the standard code). This is in line with the observation that in most fungi, plants, and animals, this genomic parameter is in the range from 35 to 50%. By analysing natural sequences, we show that there is a clear bias for triplets corresponding to stop codons near the 5′- and 3′-splice sites in the introns of various clades.

## Introduction

A frequently used method in gene finding is based on determining codon frequencies and comparing them with frequencies in known coding and non-coding sequences^1–3^. For example, in protein-coding sequences, stop codons (a.k.a. translation termination codons) occur less often than expected just by chance. A generic case is provided by sequences without any bias. These can be considered as pseudo-random sequences, especially in the cases where they code for highly complex proteins^4^.

Different definitions of sequence complexity have been proposed^5,6^. Here, we use this term in its intuitive meaning, implying that complex sequences involve a high variability in composition (triplets or amino acids) and only few repeats (see also Discussion). Examples of proteins with high sequence complexity are enzymes and regulatory proteins, in contrast to structural proteins or ALU repeats, which often involve or consist of regions of biased composition containing simple sequence repeats, which lead to low complexity^7–10^. Since the GC content differs considerably among species, it is worthwhile analyzing pseudo-random sequences under the side constraint that the percent GC is fixed and may differ from 50%. Obviously, the codon frequency depends on this parameter. For example, it was argued that the high GC content of grass genomes and the structure of the triplets encoding for alanine, GCN, may contribute to its frequent occurrence^10^. Moreover, a positive linear correlation between the GC contents in coding sequences and in the genomic region containing these sequences was found^11^.

Many organisms show differences in the frequency of occurrence of synonymous codons, a phenomenon called codon bias^12^. This bias can vary not only from organism to organism, but also from gene to gene within the same organism^13^. Here, we consider the case where the codon bias is only determined by the GC content. In particular, this implies the assumption that the GC skew and AT skew are zero. For example, isoleucine is encoded by AUA, AUU and AUC. If percent GC is low, then AUA and AUU occur more often than AUC. In contrast, a gene- or organism specific bias towards AUA or AUU is neglected here. It has indeed been found in human and mouse genes that the higher the GC content is, the higher is the usage of C. This is mainly seen in the third codon position (wobble base) and to some extent in the first position^11^. However, since we here study pseudo-random sequences, we neglect the different effects on codon positions.

Interestingly, also many non-coding sequences such as intergenic regions and constitutively spliced introns can be considered to be pseudo-random if they are subject to low selection pressure. In this context, it is worth mentioning that several lines of evidence suggest that novel genes (or their precursors, sometimes called proto-genes) can emerge also from non-coding regions^14–16^. Since start and end signals for transcription as well as splice sites are rather short, they are expected to occur frequently even in random sequences^14^. This facilitates de novo emergence of genes. Tautz and coworkers^15^ expressed clones with synthetically generated random sequences (as equimolar mixes of A, C, G and T) in *Escherichia coli* and showed that transcribed and translated random sequences could indeed have a high potential to become functional. In view of all the above-mentioned observations, we consider it useful to analyse highly complex sequences, which we assume to be quasi-random.

Several decades ago, Temple Smith^17^ (especially well-known for the Smith-Waterman algorithm) calculated, for the standard genetic code (SGCode), the frequency of each amino acid based on its set of codons and as a function of GC content and determined the inherent Shannon information^18,19^ for this amino acid frequency distribution. Furthermore, he calculated the GC content at which the Shannon information has its maximum. Hasegawa and Yano^20^ extended this work by considering stationary second-order Markov chains. Mir et al.^3^ introduced a geometric model for the evaluation of several genome statistics in bacteria, like ORF number and length distribution, in dependence on codon usage and GC content. For the special case of stop codons, we have presented a statistical analysis of codon distribution in dependence on GC content previously^2^.

Here, we perform the above-mentioned statistical analyses^2,4,17^ in more detail and extend them by considering 25 genetic codes. Although the SGCode and its codon assignments are predominantly used in almost all life forms^21,22^, variations exist, for example, in some archaea, eubacteria (especially those with small genomes), yeasts as well as mitochondria and several types of plastids^23,24^. As of August 2022, the National Center for Biotechnology Information (NCBI) catalogued 24 alternative codes^25^.

In particular, we determine several features in dependence on GC content, because that parameter differs from 50% in many genomes and the evolution of de novo genes depends on that parameter^16^. Our analysis is aimed at two main applications: calculating the variability of proteins (expressed by Shannon’s information) and determining the frequency of translation termination codons. We show where the frequency functions reach their maxima, that is, for which GC content a given amino acid would occur most often in pseudo-random sequences. We also calculate how much information is contained in such sequences for each genetic code in dependence on GC content using Shannon’s entropy equation. In doing so, we consider the different codon numbers of the different amino acids and the stop codon. Therefore, the information content differs from what would be obtained by just considering the distribution of nucleotides. Additionally, we analyse the GC contents of the genomes of archaea, eubacteria, fungi, plants, protozoa, invertebrates, vertebrates, and viruses and compare them with the calculated GC content at maximum information.

The second, related goal of our paper concerns the distribution of stop codons. As mentioned above, in protein-coding sequences, those triplets occur less often than expected by chance. Thus, in the SGCode, for a GC content of 50%, a termination codon will appear less often than at every 64/3 ≈ 21st triplet^2,3^. Accordingly, de novo genes should emerge more frequently in genomic regions with elevated GC content because these tend to involve fewer AT-rich stop codons^16^. For our analysis, it is important that this average distance depends not only on GC content, but also on the genetic code used. The *thraustochytrium mitochondrial* code, for example, includes an additional stop codon, UUA, so that at 50% GC content every 16th codon would encode termination purely by chance. In the alternative flatworm mitochondrial code, there is only the “amber” triplet UAG, which would occur purely by chance at every 64th triplet. In the first part of our study, in which we analyse pseudo-random sequences, we neglect the property of stop codons to occur less often in protein-coding sequences than expected by chance.

Stop signals are relevant in the definition of ORFs. In their most basic definition, ORFs are nucleotide sequences that are enclosed by a start and a stop codon, whose lengths are divisible by three and that do not have any other stop codons in between^3,28,29^. While this definition is sufficient as a first step for gene finding in prokaryotes^2,3^, it often fails to be applicable in eukaryotes due to the presence of introns^28,30^. Most introns contain sequences that would be stop codons if in a coding region and/or cause shifts between reading frames. Henceforth, we use the term stop signal in the general case where it is not yet clear whether or not the sequence is coding a protein sequence.

A further problem with the traditional ORF definition is the occurrence of alternative start codons^28,31^. A third problem is that the 5′ and 3′ untranslated regions are part of the gene and transcript while not being included in the start-to-stop stretch^32^. For all of these reasons, an alternative ORF definition is often used, especially in gene finding software, saying that an ORF is delimited by two consecutive stop codons^4,28,33^. Extending our analysis from pseudo-random sequences to natural genomes, we will here investigate, by empirical analysis, the stop signal distribution in introns of hundreds of genomes from several kingdoms of life. We compare those results to the predicted distribution for a given GC content. This is relevant for the question as to how far a predicted ORF according to the alternative definition extends into an intron, although mainly exonic sequences are searched for in gene finding.

## Methods and data

### Genetic codes

The mapping tables of the 25 known genetic codes are taken from the NCBI genetic code databank^25^.

### Frequencies of amino acids and stop signal in pseudo-random sequences

We determine the frequency of each amino acid according to the equations presented by Smith^17^, Pohl et al.^2^ and Mir et al.^3^. Since we consider pseudo-random sequences, our calculations are independent of the reading frame. According to Chargaff’s second parity rule, the frequencies of the complementary bases in each strand are (almost) equally distributed, that is, P(A) ≈ P(U) and P(G) ≈ P(C)^34–36^. Therefore, the frequency of each base is dependent on the GC content – denoted here by *g*:

1 $${P}_{G}\left(g\right)={P}_{C}\left(g\right)=\frac{g}{2}$$

2 $${P}_{A}\left(g\right)={P}_{U}\left(g\right)=\frac{1-g}{2}$$

For pseudo-random sequences, statistical independence of the nucleotide positions can be assumed. Thus, the probability of a codon can be calculated by multiplying the frequency of each base in the triplet. For example, the frequency of the “amber” triplet UAG is as follows:

3 $${P}_{UAG}\left(g\right)={P}_{U}\left(g\right)*{P}_{A}\left(g\right)*{P}_{G}\left(g\right)=\frac{\frac{{\left(1-g\right)}^{2}}{4}*g}{2}=\frac{g-2{g}^{2}+{g}^{3}}{8}$$

The expected frequency of an amino acid is calculated by summing up the probability of each codon by which it is encoded. In the analysis of pseudo-random sequences, we neglect that stop codons usually occur less often in protein-coding sequences than expected by chance. Thus, for the SGCode, the expected frequency of the stop signal is as follows^2^:

4 $${P}_{SGCode,Stop}\left(g\right)={P}_{UAA}\left(g\right)+{P}_{UAG}\left(g\right)+{P}_{UGA}\left(g\right)=\frac{1-3g+3{g}^{2}-{g}^{3}}{8}+2*\frac{g-2{g}^{2}+{g}^{3}}{8}=\frac{{g}^{3}-{g}^{2}-g+1}{8}$$

This is done analogously for all canonical amino acids and genetic codes by calculating the frequencies for all GC contents (Supplement S4).

Each codon-to-amino-acid assignment is usually unique. In our calculations, we take into account that in some alternative codes, codon assignment is non-unique for some canonical amino acids or translation stop. For example, in the *ascidian mitochondrial* code, the codons AGA and AGG can code for glycine, arginine or serine. In the mitochondrial genome of *Halocynthia roretzi*, which uses that code, the tRNA with the anticodon UCU encodes glycine when the first uracil is a 5-carboxymethylaminomethyl-uridine (cmnm^5^U)^26,27^.

In this case, the codon frequency is evenly split between the respective signals for simplicity’s sake. For the example mentioned above, AGA and AGG are assigned by 1/3 to each of the amino acids glycine, arginine, and serine. All cases of non-unique assignments are outlined in the Supplement S5.

### Shannon’s entropy of genetic codes

Finally, we calculate the inherent information content of each code given the frequencies of each amino acid and the stop signal as a function of GC content using Shannon’s entropy equation^18^:

5 $$I=-{\sum }_{i}^{n}{p}_{i}*{log}_{2}{p}_{i},$$

where $n$ is the number of all signals and ${p}_{i}$ is the frequency of amino acid $i$ or the stop signal in pseudo-random sequences based on its codon number. In addition, for each genetic code, we numerically calculate at which GC content the maximum entropy is reached. This is in accordance with an optimality principle saying that complex proteins should have as much variability as possible (measured by Shannon’s information).

### Impact on ORF definition

The probability of the absence of a stop codon in a stretch of *c* triplets for a given GC content is calculated, starting from any given point^2^ (see also^37^). Thus, the probability of a sequence involving *c* triplets and at least one stop signal for a given GC content is as follows (for the SGCode):

6 $$p\left(c,g\right)=1-{\left(1-{P}_{SGCode,Stop}\left(g\right)\right)}^{c}=1-{\left(1-\frac{{g}^{3}-{g}^{2}-g+1}{8}\right)}^{c},$$

where ${P}_{SGCode,Stop}\left(g\right)$ is given by Eq. (4).

To obtain the minimum required triplet length for a given sequence probability with at least one stop codon, we solve Eq. (6) for *c*:

7 $$c\left(p,g\right)=\frac{{log}_{2}\left(1-p\left(c,g\right)\right)}{{log}_{2}\left(1-{P}_{SGCode,Stop\left(g\right)}\right)}=\frac{{log}_{2}\left(1-p\left(c,g\right)\right)}{{log}_{2}\left(1-\frac{{g}^{3}-{g}^{2}-g+1}{8}\right)}$$

We perform the same calculations for all other genetic codes and for all proteinogenic amino acids (Supplement S6).

### Genome data

The genome files in Fasta format and the genetic information files in GFF format of the genomes of all archaea, bacteria, fungi, plants, protozoa, invertebrates, vertebrates, and viruses currently available in the NCBI Genome RefSeq database (https://ftp.ncbi.nlm.nih.gov/genomes/refseq/, 19/12/2022) are retrieved using a custom Python script (v3.10). All species names and RefSeq IDs are given in Supplement S7.

### Genome GC content

The GC contents of the above-mentioned genomes are determined using the Bio.SeqUtils library of the Biopython package (v1.79). If the genome file of an organism contains more than one sequence (scaffolds, chromosomes, etc.), the average GC content over all sequences is taken.

### Intron stop signal distribution

By data mining in the above-mentioned genomes, we examine the relative frequency of stop signals per triplet in all three frames of all introns near both splice sites (for intron with length n → 5′-splice site: positions at 1 to 3, 2 to 4 and 3 to 5; 3′-splice site: positions at n − 4 to n − 2, n − 3 to n − 1 and n − 2 to n) as well as in the in-between (non-splice site) intron sequence. For the 5′- and 3′-splice sites of each intron, the stop signal frequencies are calculated by counting the number of stop codons in the three frames divided by the overall number of introns for this organism. For the intermediate sequences, the number of initial nucleotide positions is determined for each reading frame in all introns. For example, for two hypothetical introns with length five and seven, we can write {0, 1, 2, 0, 1} and {0, 1, 2, 0, 1, 2, 0}, where the numbers indicate the three reading frames. Now we can ignore the last two positions in each intron because they cannot form triplets. Next, we count the number of stop signals over all introns, for each frame separately. These counts are divided by the overall number of triplets (over all introns together) in the respective frame. In the above examples, that number equals three for frame zero (one coming from the first intron and two from the second), three for frame one and two for frame two.

To be able to define the three intron regions sufficiently (near 5′-splice site, near 3′-splice site and intermediate sequence), all introns with lengths less than 100 nt are removed for this analysis (Table 1).

**Table 1:** Number of organisms and introns for each clade as available from the NCBI Genome RefSeq database*.

Clade	#Species	#Introns
Archaea	1,027	–
Eubacteria	461	–
Protozoa	94	771,653
Fungi	481	1,605,788
Plants	157	6,873,069
Invertebrates	316	24,464,214
(Non-mammalian) Vertebrates	296	60,714,298
Mammals	189	38,186,671
Viruses	11,542	–

*Downloaded from ftp.ncbi.nlm.nih.gov/genomes/refseq/ (19/12/2022). Here, we only count introns with a minimum length of 100 bases and do not consider archaeal, bacterial, and viral introns.

## Results

In the Results section, we focus on the amino acid (and stop codon) frequencies of the SGCode. The results for the 24 alternative codes are shown in Supplement S1.

### Amino acid and stop codon frequencies for the standard genetic code

While Pohl et al.^2^ only calculated the frequency of stop codons (as a function of GC), Smith^17^ did so for all amino acids. However, he had only shown the calculation for phenylalanine explicitly. Here, we show the calculations, by way of example, for the amino acids serine and lysine in the SGCode:

8 $${P}_{SGCode,Ser}\left(g\right)={P}_{AGG}\left(g\right)+{P}_{AGU}\left(g\right)+{P}_{UCA}\left(g\right)+{P}_{UCC}\left(g\right)+{P}_{UCG}\left(g\right)+{P}_{UCU}\left(g\right)=3*\frac{1-g}{2}*\frac{{g}^{2}}{4}+3*\frac{(1-{g)}^{2}}{4}*\frac{g}{2}=\frac{3g-3{g}^{2}}{8}=\frac{3g(1-g)}{8}$$

9 $${P}_{SGCode,Lys}\left(g\right)={P}_{AAA}\left(g\right)+{P}_{AAG}\left(g\right)=\frac{{\left(1-g\right)}^{3}}{8}+\frac{{\left(1-g\right)}^{2}}{4}*\frac{g}{2}=\frac{1-2g+{g}^{2}}{8}=\frac{{\left(1-g\right)}^{2}}{8}$$

The equations for the remaining amino acids for the SGCode can be found in Table 2. Note that four formulas are cubic functions, which is understandable because the frequencies of three nucleotides are multiplied. However, the remaining formulas are quadratic functions because two cubic terms cancel each other (see Eq. (9)). Importantly, all amino acids for which the functions are quadratic are encoded by an even number of codons.

**Table 2:** Frequency equations for each amino acid (including stop signal) in random sequences for the SGCode, their maximum frequency, and GC content where that is reached.

Amino acid	Frequency equation	Max GC content	Max frequency
Asn, Lys, Phe, Tyr	$\frac{{\left(1-g\right)}^{2}}{8}$	0.0	0.125
Leu	$\frac{1-{g}^{2}}{8}$	0.0	0.125
Stop	$\frac{{\left(1-g\right)}^{2}\left(1+g\right)}{8}$	0.0	0.125
Ile	$\frac{{\left(1-g\right)}^{2}\left(2-g\right)}{8}$	0.0	0.25
Met	$\frac{g{\left(1-g\right)}^{2}}{8}$	0.33	0.0185
Asp, Cys, Gln, Glu, His	$\frac{g\left(1-g\right)}{8}$	0.5	0.03125
Thr, Val	$\frac{g\left(1-g\right)}{4}$	0.5	0.0625
Ser	$\frac{3g\left(1-g\right)}{8}$	0.5	0.09375
Trp	$\frac{{g}^{2}\left(1-g\right)}{8}$	0.67	0.0185
Ala, Gly, Pro	$\frac{{g}^{2}}{4}$	1.0	0.25
Arg	$\frac{g\left(1+g\right)}{8}$	1.0	0.25

**Figure 1:**
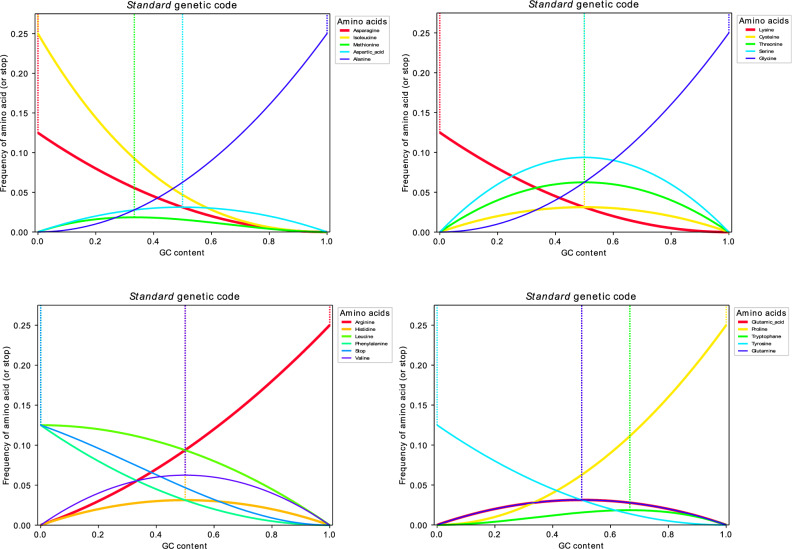
Frequencies of all amino acids (including stop signal) as encoded by the SGCode in random sequences as a function of GC content between 0 and 100%. For better visibility, the 20 amino acids and stop signal were grouped into four sets. The dashed lines mark the maximum achieved frequency for each group of synonymous codons.

It is of interest to see where these functions reach their maxima. In the SGCode, the maximum is reached at five different positions for different amino acids, notably at GC contents of 0%, 33.33%, 50%, 66.67% and 100% (Fig. 1). The maxima in the interior of the admissible interval, notably at 33.33% and 66.67%, correspond to methionine and tryptophan, respectively. Both amino acids only have one codon. This means, for example, that in a random sequence, methionine (and, thus, the start codon) occurs with the highest frequency of 1.85% for a GC of 33.33% (in contrast to 1/64 ≈ 1.56% at a GC content of 50%).

At 0% GC content, obviously, only those amino acids encoded by at least one codon only involving A and/or U, that is, asparagine, isoleucine, leucine, lysine, phenylalanine, tyrosine, and the stop signal can occur. Their frequency then is 12.5%, except for isoleucine with 25%. Isoleucine is the only amino acid encoded by three different codons: the purely AU-codons AUA and AUU as well as AUC.

Aspartic acid, cysteine, glutamic acid, glutamine, histidine, serine, threonine, and valine reach their maximum at 50% GC content. Threonine and valine then have a frequency of 6.25% (encoded by four codons) and serine has 9.375% (encoded by six codons) while the remaining amino acids (encoded by two codons) have 3.125%. Finally, at 100% GC content, only alanine, arginine, glycine, and proline can occur, notably with a frequency of 25%. They are all encoded by four codons (two of which are pure GC codons), except arginine, which is encoded by six.

For the 24 other genetic codes considered, apart from the maxima of expected frequencies at 0%, 33.33%, 50%, 66.67% and 100% GC (see above), eight alternative maxima arise for the amino acids cysteine, glutamine, leucine, serine and tryptophan. For example, cysteine and glutamine reach their maxima at 42.26% GC content in the *euplotid nuclear* and *ciliate-dasycladacean-hexamita nuclear* codes, respectively. Moreover, GC values where maxima are situated, include 18.35%, 45.14%, 46.48%, 47.74%, 53.52%, 54.86%, and 57.74%. Interestingly, every amino acid in the *ascidian mitochondrial*, *invertebrate mitochondrial*, *rhabdopleuridae mitochondrial*, v*ertebrate mitochondrial*, and *yeast mitochondrial* codes is encoded by at least two codons and all maxima are reached at 0%, 50% or 100% GC (for more detail, see Supplement).

**Figure 2:**
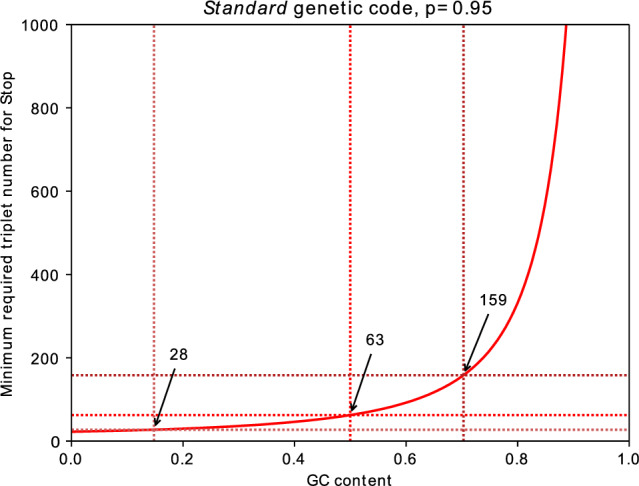
Number of triplets in a random sequence so as to contain at least one stop codon with a probability of 95% using the SGCode as a function of GC content. The horizontal and vertical dashed lines indicate the number of triplets for GC contents of 14.8% (28 triplets) as found in the protozoon *Leishmania braziliensis*, 50% (63 triplets) as found in *E. coli* and 70.3% (159 triplets) in the slime mold *Fonticula alba*.

For gene finding and for the stop-to-stop ORF definition, the frequency of stop codons is of interest. As mentioned above, at 50% GC content, on average every 64/3 ≈ 21^st^ codon in a random sequence would be a termination codon just by chance alone. However, the distance fluctuates around this average value according to a monotonic decreasing exponential distribution with respect to the distance^4^. The curve of the function given in Eq. (7) is shown in Fig. 2. For GC contents tending to 100%, stop signals occur less and less often. Mathematically, the cubic polynomial in the denominator in Eq. (7) then tends to zero, so that the argument of the logarithm tends to one and the reciprocal of the logarithm diverges. Thus, the curve grows very steeply near 100% GC.

We can calculate the minimum sequence length so that at least one stop codon occurs with a probability of 95%. At 50% GC content and with the SGCode, a length of 63 triplets (or 189 nt) is obtained (Fig. 2). Since the average length of introns in the human genome, for example, equals about 1806 triplets (5419 nt)^38,39^ and is, thus, much longer than 63 triplets, introns practically always contain a stop signal in any reading frame.

For the alternative codes, the values at a GC content of 50% are given in Supplement S1. The lowest length of 47 triplets is calculated for the *thraustochytrium mitochondrial* and *vertebrate mitochondrial* genetic codes. These are the only two codes where the stop signal is encoded by an additional triplet (compared to the SGCode), namely UUA, which reduces the required length. This is in contrast to the *karyorelict nuclear* genetic code where the stop signal is only encoded by the “opal” triplet UGA, which can also be transcribed into tryptophan. In this case, the highest length of 382 triplets is obtained. Besides those two, four additional lengths are calculated (see Supplement S1).

### Maximum potential information at around 43% GC

**Figure 3:**
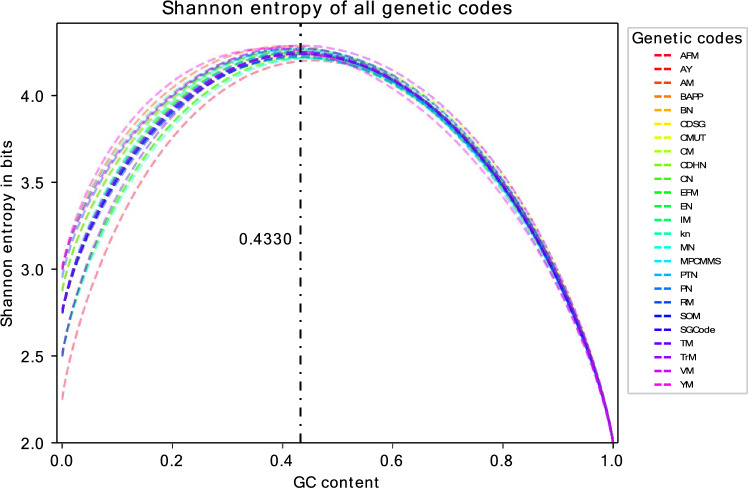
Entropies of 25 genetic codes for their given codon assignments and GC contents between 0 and 100% as calculated by Shannon’s entropy Eq. (5). The dash-dotted line indicates the GC content (43.3%) at which the SGCode reaches its entropy maximum (4.24 bits). The abbreviations are as follows: AFM, *Alternative Flatworm Mitochondrial*; AY, *Alternative Yeast*; AM, *Ascidian* *Mitochondrial*; BAPP, *Bacterial-Archaeal-Plant Plastid*; BN, *Blastocrithidia Nuclear*; CDSG, *Candidate Division SR1 and Gracilibacteria*; CMUT, *Cephalodiscidae Mitochondrial UAA-Tyr*; CM, *Chlorophycean Mitochondrial*; CDHN, *Ciliate-Dasycladacean-Hexamita Nuclear*; CN, *Condylostoma Nuclear*; EFM, *Echinoderm-Flatworm Mitochondrial*; EN, *Euplotid Nuclear*; IM, *Invertebrate Mitochondrial*; KN, *Karyorelict Nuclear*; MN, *Mesodinium Nuclear*; MPCMMS, *Mold-Protozoan-Coelenterate Mitochondrial* and *Mycoplasma Spiroplasma*; PTN, *Pachsyolen Tannophilus Nuclear*; PN, *Peritrich Nuclear*; RM, *Rhabdopleuridae Mitochondrial*; SOM, *Scenedesmus Obliquus Mitochondrial*; SGCode, *Standard Genetic Code*; TM, *Thraustochytrium Mitochondrial*; TrM, *Trematode Mitochondrial*; VM, *Vertebrate Mitochondrial*; YM, *Yeast Mitochondrial*.

Using the expected amino acid (including stop signal) frequencies of the various genetic codes as input for Shannon’s entropy, we determined their potential information content and at what GC content the codes reach their maximum entropies (Fig. 3). The optimal GC content for the SGCode is 43.3%. The entropy value then amounts to 4.24 bits, which is near the maximum possible value of log_2_(20) ≈ 4.32 bits achieved upon equal distribution of amino acids. For the alternative codes, the values are given in Supplement S2. The lowest GC content implying maximum information is for the *yeast mitochondrial* code with 38.11% (the only code for which the optimum is reached at a GC below 40%), while the highest is for the *alternative flatworm mitochondrial* genetic code with 45.61%. Note that at 100% GC for all genetic codes, the Shannon entropy equals two bits because only the four amino acids alanine, arginine, glycine, and proline can be encoded then and are equally distributed. On the other hand, at 0% GC content, the entropies are between 2.25 bits (for the *alternative flatworm mitochondrial* genetic code) and 3 bits (for the *ascidian mitochondrial*, *invertebrate mitochondrial*, *vertebrate mitochondrial* and *yeast mitochondrial* genetic codes).

### GC contents of fungi, plants and metazoa cluster around 40%

**Figure 4:**
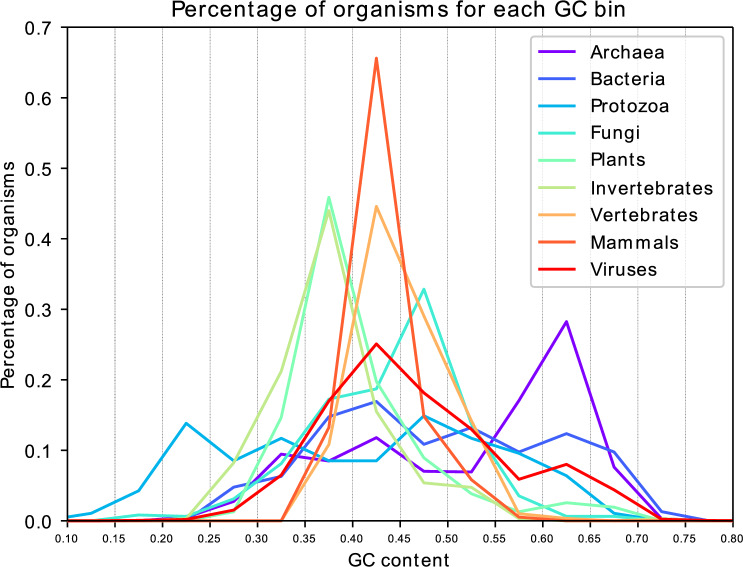
Percentages of organisms for the clades archaea, bacteria, protozoa, fungi, plants, invertebrates, non-mammalian vertebrates, mammalian vertebrates and viruses with given genomic GC contents binned in 5% intervals. For the numerical data, see Supplement S3.

Looking at the distribution of genomic GC contents across the clades, it can be seen that in complex organisms, notably fungi, plants, invertebrates, and vertebrates, the genomic GC contents are mainly in the range from 35 to 50% (Fig. 4). Especially in non-mammalian and mammalian vertebrates, around 44.6% and 65.6% of genomes, respectively, have a GC content between 40 and 45% which coincides with the maximum obtained information content in the SGCode. In contrast, GC contents in the genomes of less complex organisms, notably archaea, eubacteria, protozoa, and viruses, are distributed across a GC range from 10 to 70%. Extreme cases are the protozoon *Leishmania braziliensis* with a GC content of 14.8% and the slime mold *Fonticula alba* with a GC content of 70.3%. Less than 50% of lower genomes have GC contents between 35 and 50% except for viral genomes.

### 5′ and 3′-splice sites are biased for stop signals

**Figure 5:**
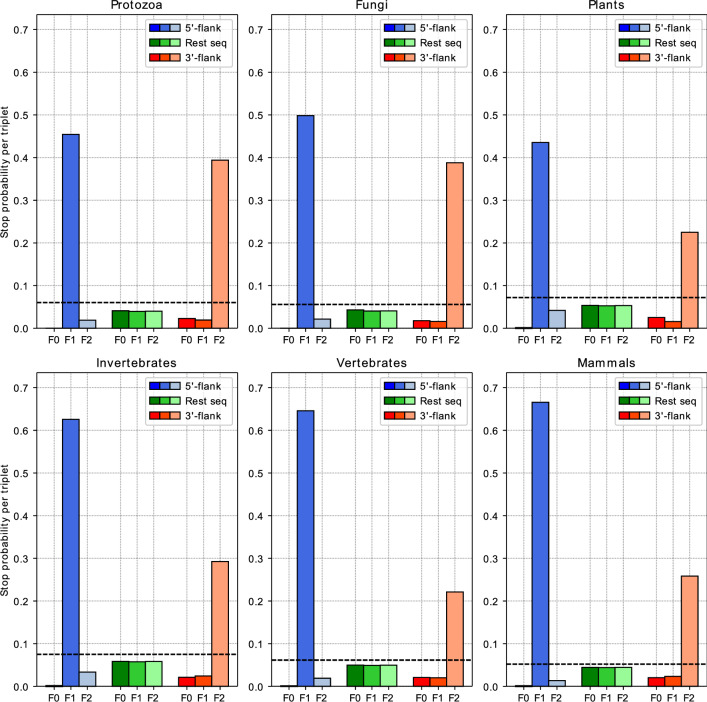
Frequencies of stop signals in the first and last three triplets as well as the remaining sequence for all three frames derived from the introns of genomes of the six clades protozoa, fungi, plants, invertebrates, mammalian vertebrates and non-mammalian vertebrates (Table 1). ‘5′-flank’ (blue) indicates intron positions 1 to 3, 2 to 4 and 3 to 5. ‘3′-flank’ (red) indicates intron positions n − 4 to n − 2, n − 3 to n − 1 and n − 2 to n. ‘Rest seq’ (green) indicates the average stop signal frequency in the intermediate intron sequences between both flanks from positions 6 to n − 5. ‘F0’ − ‘F2’ indicate the frames. The dashed line indicates the probability calculated with Eq. (4) given the average GC content of each in-between sequence averaged over all sequences. For the numerical data, see Supplement S3.

In view of the stop-to-stop definition of ORFs, we looked at the stop signal frequencies in introns of fungi, plants, protozoa, invertebrates, and vertebrates. All six groups of organisms show very similar results (Fig. 5). There seems to be a clear bias in introns near the 5′- and 3′-splice sites (i.e., acceptor and donor splice sites, respectively) for the occurrence of a stop signal. In the genomes of invertebrates, non-mammalian, and mammalian vertebrates over 60% of introns contain a stop signal in nucleotide positions 2–4 downstream of the 5′-splice sites (i.e., at the next triplet position in frame 1). In introns of fungi, plants and protozoa, such triplets are also enriched at the same position but with lower frequencies. Near 3′-splice sites in introns of plants, invertebrates, non-mammalian, and mammalian vertebrates, stop signals appear in frame 2 with frequencies between 20 and 30%. Frequencies are considerably higher in protozoan and fungal introns, notably 39.4% and 38.8%, respectively. This finding corroborates the suitability of the ORF definition in terms of stop-to-stop.

For the in-between sequences, the stop signal frequencies determined by data mining range from around 4% to around 6% per triplet. The calculated probabilities given the average GC content over all intermediate sequences and the SGCode range from 5.2% (mammals) to 7.5% (invertebrates).

## Discussion

Here, we have calculated the frequencies of all groups of synonymous codons in pseudo-random sequences in dependence on GC content. We neglected any codon bias apart from that resulting from the varying GC content. Following earlier approaches^4,17^, we use pseudo-random sequences as a proxy for highly complex DNA sequences such as encoding enzymes or regulatory proteins (coding) or introns (non-coding). It should be noted, however, that a random sequence need not have maximum complexity (i.e., Kolmogorov complexity)^40^. A long random sequence can contain a repeat like AAAA, while this cannot occur in the maximally complex sequence because it can be compressed to 4A.

In our calculations, we used Chargaff’s second parity rule saying that the frequencies of G and C are equal in each strand, and so are those of A and T. However, this rule is not fulfilled in mitochondria, plastids, single-stranded viral DNA genomes and (single- or double stranded) viral RNA genomes^41,42^. Therefore, that parity rule may not be valid in all alternative genetic codes. For simplicity’s sake, we ignored this feature here.

Based on the calculated frequencies, we have determined the potential information entropies. In the Shannon formula, we have used overall frequencies of amino acids (summed over the synonymous codons). It is worth mentioning that the formula used by Zeeberg^11^ differs in that a double sum over amino acids and over synonymous codons was used, which implies that the Shannon information is calculated on the basis of the frequencies of all codons. The mathematical difference is that the logarithm is calculated for the different amino acids in our approach (tracing back to^17^) and for the different codons in the latter approach. Therefore, the maxima are reached at different GC contents. In our calculations, the entropies reach their maxima between GC contents of about 38% and 46%. The GC content of several mammals, birds and reptiles using the SGCode are indeed between 40 and 50%^43^. For example, the GC content of the human genome is 40.9%^44^ and, therefore, only about 2% below the optimal value for the SGCode.

An interesting outcome is that the optimal GC contents do not differ considerably from each other for different genetic codes. Moreover, although the amino acids are not equally distributed, the maximum information content is very close to the maximum possible value of 4.32 bits which would be achieved in the case of equipartition. Importantly, the region around the maximum entropy (at the amino acid level) of all genetic codes is relatively flat. For example, the calculated information in the SGCode for the plant *Arabidopsis thaliana* and the green alga *Chlamydomonas reinhardtii* with GC contents of 36% and 64%^45^, respectively, is still high, notably about 4.11 bits. A similar pattern can be seen for all the other genetic codes. Even at a GC content as low as 28%, none of the entropies of any genetic code fall below 4 bits. Due to the flat shape of the maxima, genetic codes allow some flexibility in the composition of the nucleotide structure of genomes while still providing a high information encoding.

In addition to sorting out GC contents from the literature, we extracted such values also from all the genomes in the NCBI Genome RefSeq database. Thus, we were able to show that in complex organisms, genomic GC contents cluster in the regions where the SGCode reaches its maximum information content, namely in range of 35% to 50% GC content. These findings support the hypothesis put forward here that evolution has optimized the GC content to maximize variability of amino acid sequences.

However, it is unclear whether the GC content and the nucleotide structure of a genome have been mainly adapted during evolution to encode as much information as possible or some other mechanisms play key roles. It is worth noting that there are species with GC contents lower than 20% or greater than 70%. For example, the values in bacteria can range from as low as 17% (*Carsonella ruddii*) to as high as 74% (*Anaeromyxobacter dehalogenans*)^46,47^. Low GC contents can be explained by GC to AT transitions due to methylation of cytosine and subsequent deamination to thymine. This has been shown to be one of the most common mutations in both prokaryotes and eukaryotes^48–50^. However, in many genes, this is counteracted by biased gene conversion leading, on average, to a higher GC content than in non-coding regions^51,52^. In general, regions with high GC contents are associated with increased transcription^53^.

A further cause for GC drift may be related to viral defence mechanisms. Bacteria are able to discriminate between their own and foreign DNA based on differences in GC content^54^. It was also shown that bacteriophages try to mimic the GC contents of their host to evade this mechanism whereas the same could not be seen for non-bacteria-infecting viruses^55^. For example, the GC contents of vertebrate viruses can range from 33 to 70%^56^. At the same time, in our viral dataset, the majority of viruses have a GC content of 40–45% which also coincides with the GC content of most vertebrates.

An important point is that different amino acids imply different metabolic costs in their synthesis (in terms of ATP and carbon). These costs can be computed by metabolic network analyses^57,58^. A compromise needs to be found between maximum variability and minimum costs. Interestingly, there is an analogy to thermodynamics in that the minimization of free energy also implies a trade-off between maximum entropy and minimum energy^59^. This factor is implicitly included in our analysis by the different codon numbers of the amino acids. Amino acids such as tryptophan and tyrosine that are “costly” in terms of carbons and energy have lower codon numbers and, hence, occur less frequently in proteomes than “cheap” amino acids such as glycine and alanine. A correlation between metabolic costs of amino acids and codon bias was found^58^. In particular, it can be hypothesized that the factors influencing the number of codons during the evolution of genetic codes^60,61^, include metabolic costs of amino acids. It would be interesting in future studies to consider the costs more explicitly.

As a second application, we analysed the frequency of stop signals. Considering ORFs of a minimum length of 100 triplets, Pohl et al.^2^ showed that, with a significance level of p = 0.05, random and non-random distributions of stop signals can be distinguished below a GC content of 61.8%. Here, we have calculated that in pseudo-random sequences, such triplets occur often enough at those GC contents so that any intron (in the typical length range) is very likely to involve at least one of them in any reading frame. This supports the ORF definition in terms of stop-to-stop^28^. As mentioned in the introduction, Pohl et al.^2^ used their method to search in prokaryotes since their genomes do not contain any introns and, therefore, splicing is not an issue. It is worth noting that, using the stop-to-stop definition, the method is also applicable to eukaryotic genomes.

To compare our statistical analysis concerning the occurrence of stop signals with real sequence data, we performed data mining and looked at the distribution in the intron sequences of six clades. Although splicing and subsequent frameshifts pose a problem, we are able to show that there is a bias towards stop signals encoding near the 5′-splice and 3′-splice sites. At the same time, the frequencies in the other frames and the intermediate sequences clearly show depletion of stop codons. This increases the applicability of the stop-to-stop definition of ORFs even more.

Our results are further supported by the fact that a very common splice site motif in introns is GT…AG^62^. The thymine in the 5′-splice site is often followed by an adenine or guanine which gives the canonical GTR motif^63^. Since two of the three stop codons are TAA and TGA, two of the three required nucleotides are already provided by the 5′-splice site motif. Thus, there is a considerable probability that a stop signal is formed by the triplet starting at the second nucleotide of the intron sequence just by chance alone. At the 3′-splice site, the adenine is often preceded by a cytosine or thymidine, which gives the canonical YAG motif^64^. Similar to the 5′-splice site motif, there is a considerable probability that the YAG motif forms the remaining stop codon TAG in the last three nucleotides of the intron sequence just by chance alone. Overall, this fact can potentially be used in gene finding to ‘hop’ from exon to exon by following consecutive stop codons, the first one upstream of an exon (i.e., at the end of the preceding intron or in the 5′UTR) and the next one at the beginning of the following intron or the canonical termination of the final exon.

An interesting extension of our analysis is to take into account that, in many species including humans, the GC content varies considerably along their genome. Moreover, simulating the dynamics of approaching the distribution of synonymous codons at given GC content is an interesting topic for future studies. In addition to gene finding, our results may be relevant for applications in synthetic biology. For example, when synthetic genomes are constructed^65,66^, it is advantageous to optimize the GC content so as to maximize their inherent information (in the sense of variability) or to enrich specific amino acids of interest.

### Supplementary Information

Supplementary Information 1.

Supplementary Information 2.

Supplementary Information 3.

Supplementary Information 4.

Supplementary Information 5.

Supplementary Information 6.

Supplementary Information 7.

## References

[1] Lin MF, Jungreis I, Kellis M (2011). PhyloCSF: A comparative genomics method to distinguish protein coding and non-coding regions. Bioinformatics.

[2] Pohl M, Theiβen G, Schuster S (2012). GC content dependency of open reading frame prediction via stop codon frequencies. Gene.

[3] Mir K, Neuhaus K, Scherer S, Bossert M, Schober S (2012). Predicting statistical properties of open reading frames in bacterial genomes. PLoS ONE.

[4] Claverie J-M, Poirot O, Lopez F (1997). The difficulty of identifying genes in anonymous vertebrate sequences. Comput. Chem..

[5] Koslicki D (2011). Topological entropy of DNA sequences. Bioinformatics.

[6] Li M, Vitányi P (2008). An introduction to Kolmogorov complexity and its applications.

[7] Nandi T, Dash D, Ghai R, B-Rao C, Kannan K, Brahmachari SK, Ramakrishnan C, Ramachandran S (2003). A novel complexity measure for comparative analysis of protein sequences from complete genomes. J. Biomol. Struct. Dyn..

[8] Kato M, Zhou X, McKnight SL (2022). How do protein domains of low sequence complexity work?. RNA.

[9] Batzer MA, Deininger PL (2002). Alu repeats and human genomic diversity. Nat. Rev. Genet..

[10] Kottenhagen, N., Gramzow, L., Horn, F., Pohl, M. & Theißen, G. Polyglutamine and polyalanine tracts are enriched in transcription factors of plants. In *Proceedings GCB* (2012).

[11] Zeeberg B (2002). Shannon information theoretic computation of synonymous codon usage biases in coding regions of human and mouse genomes. Genome Res..

[12] Hershberg R, Petrov DA (2008). Selection on codon bias. Ann. Rev. Genet..

[13] Gustafsson C, Govindarajan S, Minshull J (2004). Codon bias and heterologous protein expression. Trends Biotechnol..

[14] Neme R, Tautz D (2013). Phylogenetic patterns of emergence of new genes support a model of frequent de novo evolution. BMC genomics.

[15] Neme R, Amador C, Yildirim B, McConnell E, Tautz D (2017). Random sequences are an abundant source of bioactive RNAs or peptides. Nat. Ecol. Evolut..

[16] Casola C (2018). From de novo to “de nono”: the majority of novel protein-coding genes identified with phylostratigraphy are old genes or recent duplicates. Genome Biol. Evol..

[17] Smith TF (1969). The genetic code, information density, and evolution. Math. Biosci..

[18] Shannon CE (1948). A mathematical theory of communication. Bell Syst. Tech. J..

[19] Schneider TD (2010). A brief review of molecular information theory. Nano Commun. Netw..

[20] Hasegawa M, Yano T-A (1975). The genetic code and the entropy of protein. Math. Biosci..

[21] Jukes TH, Osawa S, Osawa S, Honjo T (1991). Recent evidence for evolution of the genetic code. Evolution of life.

[22] Knight RD, Freeland SJ, Landweber LF (2001). Rewiring the keyboard: evolvability of the genetic code. Nat. Rev. Genet..

[23] Sengupta S, Higgs PG (2005). A unified model of codon reassignment in alternative genetic codes. Genetics.

[24] Yokobori S-I, Suzuki T, Watanabe K (2001). Genetic code variations in mitochondria: tRNA as a major determinant of genetic code plasticity. J. Mol. Evolut..

[25] Elzanowski, A. & Ostell, J. *The Genetic Codes*, NCBI. https://www.ncbi.nlm.nih.gov/Taxonomy/Utils/wprintgc.cgi. Accessed 1 August 2022.

[26] Yokobori S-I, Ueda T, Feldmaier-Fuchs G, Pääbo S, Ueshima R, Kondow A, Nishikawa K, Watanabe K (1999). Complete DNA sequence of the mitochondrial genome of the ascidian Halocynthia roretzi (Chordata, Urochordata). Genetics.

[27] Kondow A, Suzuki T, Yokobori S-I, Ueda T, Watanabe K (1999). An extra tRNA Gly (U* CU) found in ascidian mitochondria responsible for decoding non-universal codons AGA/AGG as glycine. Nucleic acids Res..

[28] Sieber P, Platzer M, Schuster S (2018). The definition of open reading frame revisited. Trends Genetics.

[29] Pevzner P (2000). Computational molecular biology: An algorithmic approach.

[30] Shafee T, Lowe R (2017). Eukaryotic and prokaryotic gene structure. WikiJ. Med..

[31] Guigó R, Knudsen S, Drake N, Smith T (1992). Prediction of gene structure. J. Mol. Biol..

[32] Min XJ, Butler G, Storms R, Tsang A (2005). OrfPredictor: Predicting protein-coding regions in EST-derived sequences. Nucleic Acids Res..

[33] Brent MR (2005). Genome annotation past, present, and future: How to define an ORF at each locus. Genome Res..

[34] Chargaff E (1950). Chemical specificity of nucleic acids and mechanism of their enzymatic degradation. Experientia.

[35] Chargaff E (1951). Structure and function of nucleic acids as cell constituents. Fed. Proc..

[36] Rudner R, Karkas JD, Chargaff E (1968). Separation of B. subtilis DNA into complementary strands. 3. Direct analysis. Proc. Natl. Acad. Sci..

[37] Ewens WJ, Grant GR (2005). Statistical methods in bioinformatics: An introduction.

[38] Sakharkar MK, Chow VTK, Kangueane P (2004). Distributions of exons and introns in the human genome. In Silico Biol..

[39] Lander ES (2001). Initial sequencing and analysis of the human germane. Nature.

[40] Pratas D, Pinho AJ, Alexandre LA (2017). On the approximation of the Kolmogorov complexity for DNA sequences. Pattern recognition and image analysis.

[41] Fariselli P, Taccioli C, Pagani L, Maritan A (2021). DNA sequence symmetries from randomness: The origin of the Chargaff’s second parity rule. Brief. Bioinform..

[42] Nikolaou C, Almirantis Y (2006). Deviations from Chargaff's second parity rule in organellar DNA: insights into the evolution of organellar genomes. Gene.

[43] Romiguier J, Ranwez V, Douzery EJP, Galtier N (2010). Contrasting GC-content dynamics across 33 mammalian genomes: Relationship with life-history traits and chromosome sizes. Genome Res..

[44] Piovesan A, Pelleri MC, Antonaros F, Strippoli P, Caracausi M, Vitale L (2019). On the length, weight and GC content of the human genome. BMC Res. Notes.

[45] Merchant SS, Prochnik SE, Vallon O, Harris EH, Karpowicz SJ, Witman GB, Terry A, Salamov A, Fritz-Laylin LK, Maréchal-Drouard L (2007). The Chlamydomonas genome reveals the evolution of key animal and plant functions. Science.

[46] Nishida H (2012). Evolution of genome base composition and genome size in bacteria. Front. Microbiol..

[47] Mann S, Chen Y-PP (2010). Bacterial genomic G+ C composition-eliciting environmental adaptation. Genomics.

[48] Ely B (2021). Genomic GC content drifts downward in most bacterial genomes. Plos One.

[49] Hershberg R, Petrov DA (2010). Evidence that mutation is universally biased towards AT in bacteria. PLoS genetics.

[50] Long H, Sung W, Kucukyildirim S, Williams E, Miller SF, Guo W, Patterson C, Gregory C, Strauss C, Stone C (2018). Evolutionary determinants of genome-wide nucleotide composition. Nat. Ecol. Evol..

[51] Bohlin J, Pettersson JH-O (2019). Evolution of genomic base composition: From single cell microbes to multicellular animals. Comput. Struct. Biotechnol. J..

[52] Pessia E, Popa A, Mousset S, Rezvoy C, Duret L, Marais GAB (2012). Evidence for widespread GC-biased gene conversion in eukaryotes. Genome Biol. Evolut..

[53] Khuu P, Sandor M, DeYoung J, Ho PS (2007). Phylogenomic analysis of the emergence of GC-rich transcription elements. Proc. Nat. Acad. Sci..

[54] Bahir I, Fromer M, Prat Y, Linial M (2009). Viral adaptation to host: A proteome-based analysis of codon usage and amino acid preferences. Mol. Syst. Biol..

[55] Mordstein C, Cano L, Morales AC, Young B, Ho AT, Rice AM, Liss M, Hurst LD, Kudla G (2021). Transcription, mRNA export, and immune evasion shape the codon usage of viruses. Genome Biol. Evol..

[56] Odon V, Fros JJ, Goonawardane N, Dietrich I, Ibrahim A, Alshaikhahmed K, Nguyen D, Simmonds P (2019). The role of ZAP and OAS3/RNAseL pathways in the attenuation of an RNA virus with elevated frequencies of CpG and UpA dinucleotides. Nucl. Acids Res..

[57] Kaleta C, Schäuble S, Rinas U, Schuster S (2013). Metabolic costs of amino acid and protein production in *Escherichia*
*coli*. Biotechnol. J..

[58] Akashi H, Gojobori T (2002). Metabolic efficiency and amino acid composition in the proteomes of *Escherichia*
*coli* and *Bacillus*
*subtilis*. Proc. Nat. Acad. Sci..

[59] Krick T, Verstraete N, Alonso LG, Shub DA, Ferreiro DU, Shub M, Sánchez IE (2014). Amino acid metabolism conflicts with protein diversity. Mol. Biol. Evolut..

[60] Buhrman H, van der Gulik PTS, Klau GW, Schaffner C, Speijer D, Stougie L (2013). A realistic model under which the genetic code is optimal. J. Mol. Evol..

[61] Szathmáry E (2003). Why are there four letters in the genetic alphabet?. Nat. Rev. Genetics.

[62] Mikl M, Hamburg A, Pilpel Y, Segal E (2019). Dissecting splicing decisions and cell-to-cell variability with designed sequence libraries. Nat. Commun..

[63] Qu W, Cingolani P, Zeeberg BR, Ruden DM (2017). A bioinformatics-based alternative mRNA splicing code that may explain some disease mutations is conserved in animals. Front. Genet..

[64] Murray JI, Voelker RB, Henscheid KL, Warf MB, Berglund JA (2008). Identification of motifs that function in the splicing of non-canonical introns. Genome Biol..

[65] Wang L, Jiang S, Chen C, He W, Wu X, Wang F, Tong T, Zou X, Li Z, Luo J (2018). Synthetic genomics: from DNA synthesis to genome design. Angew. Chem. Int. Ed..

[66] Ostrov N, Nyerges A, Chiappino-Pepe A, Rudolph A, Baas-Thomas M, Church GM (2020). Synthetic genomes with altered genetic codes. Curr. Opin. Syst. Biol..

