# Supplementary material for: Statistical analysis of synonymous and stop codons in pseudo-random and real sequences as a function of GC content
Source: Sci Rep. 2023 Dec 27;13:22996. doi: 10.1038/s41598-023-49626-9 (PMC10752896; doi:10.1038/s41598-023-49626-9)
Supplement: Supplementary file 1 — Supplementary Information 1. [file 41598_2023_49626_MOESM1_ESM.pdf]

S1

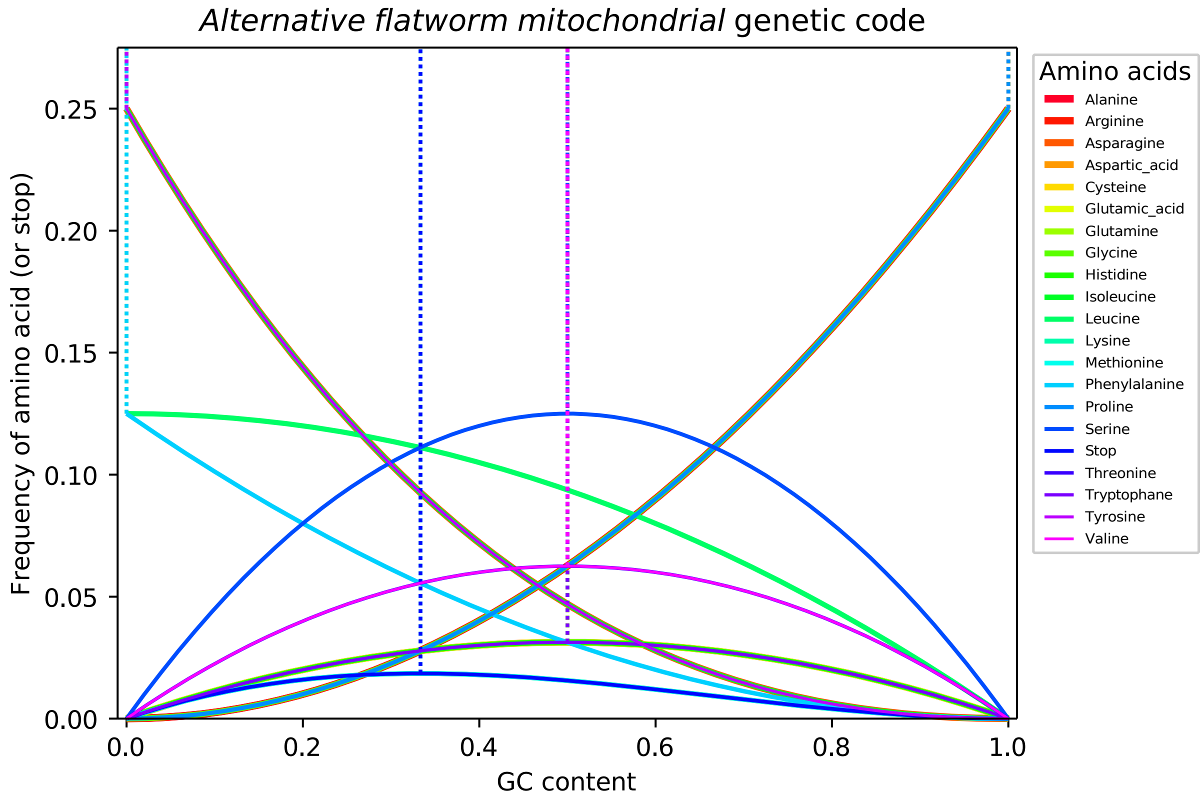

Figure S1. Frequencies of all amino acids (including stop) as encoded by the *alternative flatworm mitochondrial* genetic code in random sequences as a function of GC content between 0 % and 100 %. The dashed lines mark the maximum achieved frequency for each amino acid (including stop).

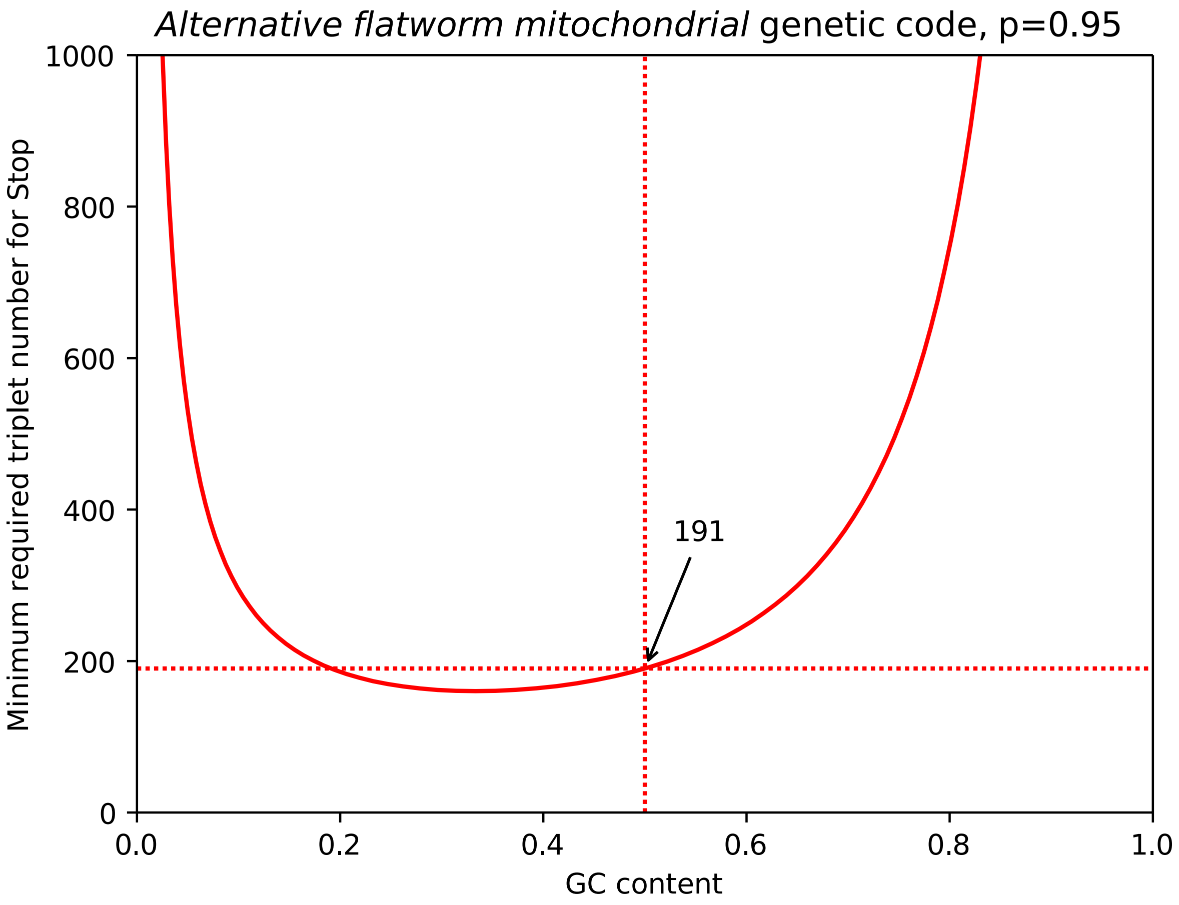

Figure S2. Number of triplets in a random sequence so as to contain at least one stop codon with a probability of 95 % using the *alternative flatworm mitochondrial* genetic code as a function of GC content. The horizontal and vertical dashed lines indicate the number of triplets for a GC content of 50 % (191 triplets).

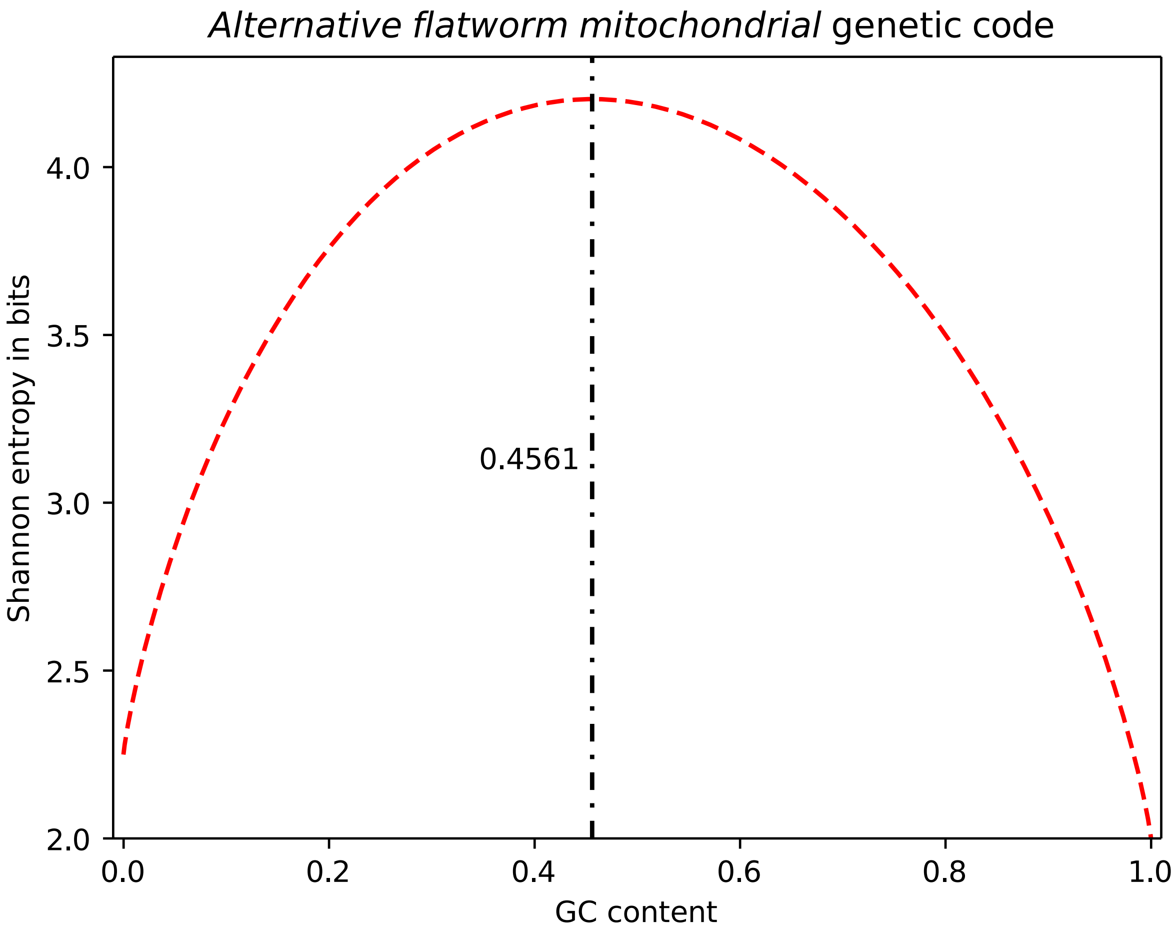

Figure S3. Entropy of the *alternative flatworm mitochondrial* genetic code for its given codon assignments and GC contents between 0 % and 100 % as calculated by Shannon's entropy equation. The dash-dotted line indicates the GC content (45.61 %) at which this code reaches its entropy maximum (4.20 bits).

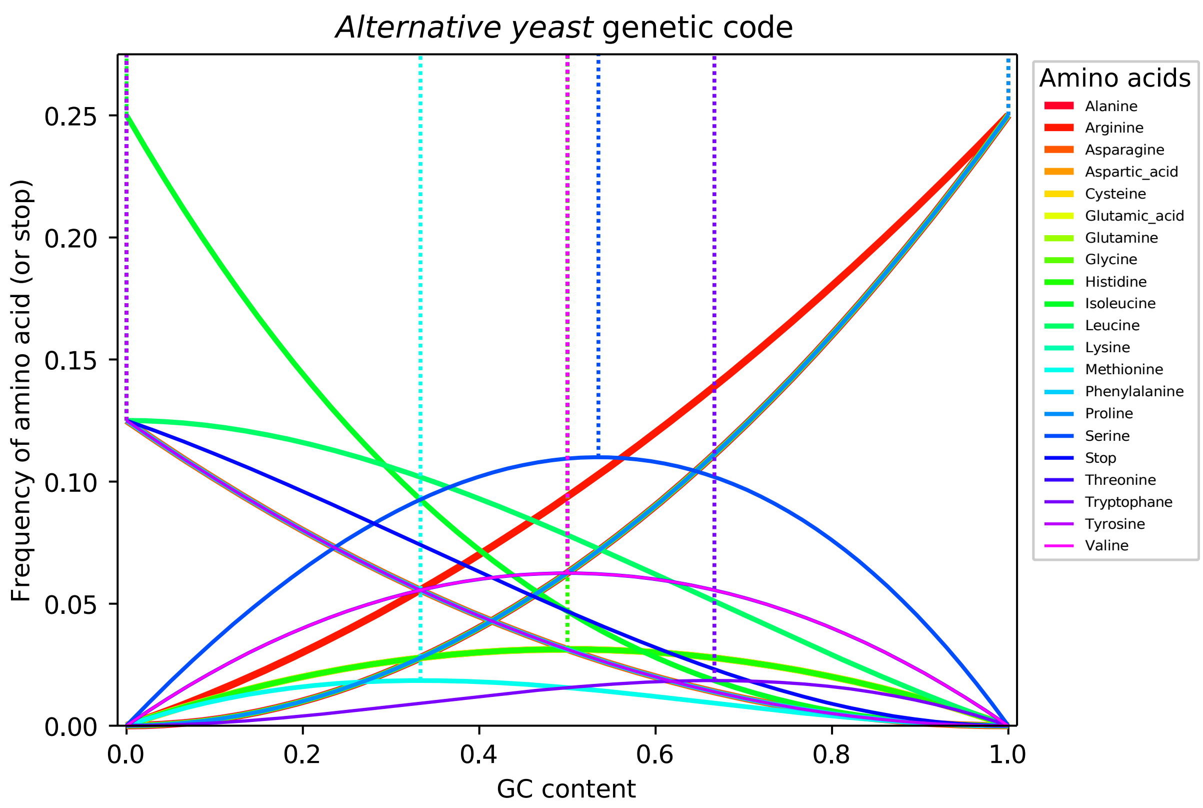

Figure S4. Frequencies of all amino acids (including stop) as encoded by the *alternative yeast* genetic code in random sequences as a function of GC content between 0 % and 100 %. The dashed lines mark the maximum achieved frequency for each amino acid (including stop).

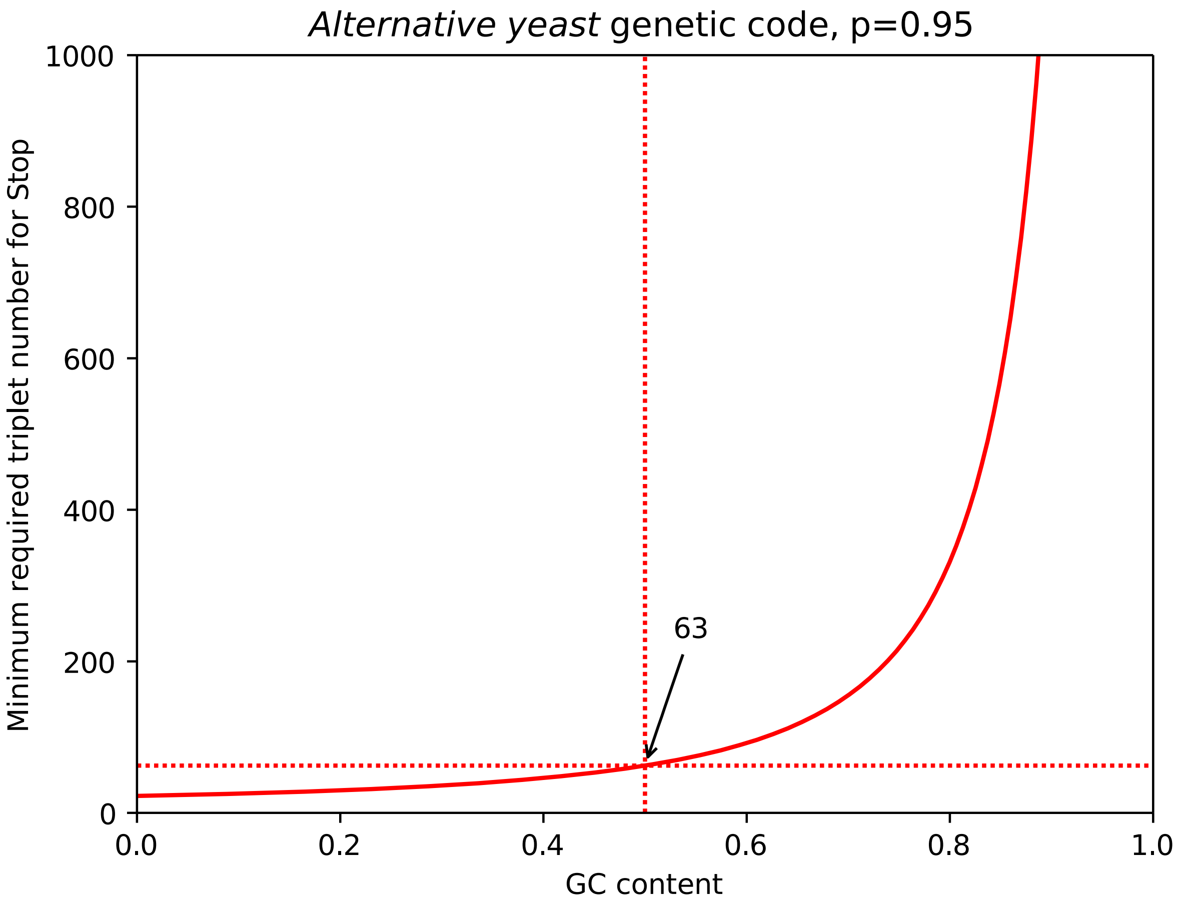

Figure S5. Number of triplets in a random sequence so as to contain at least one stop codon with a probability of 95 % using the *alternative yeast* genetic code as a function of GC content. The horizontal and vertical dashed lines indicate the number of triplets for a GC content of 50 % (63 triplets).

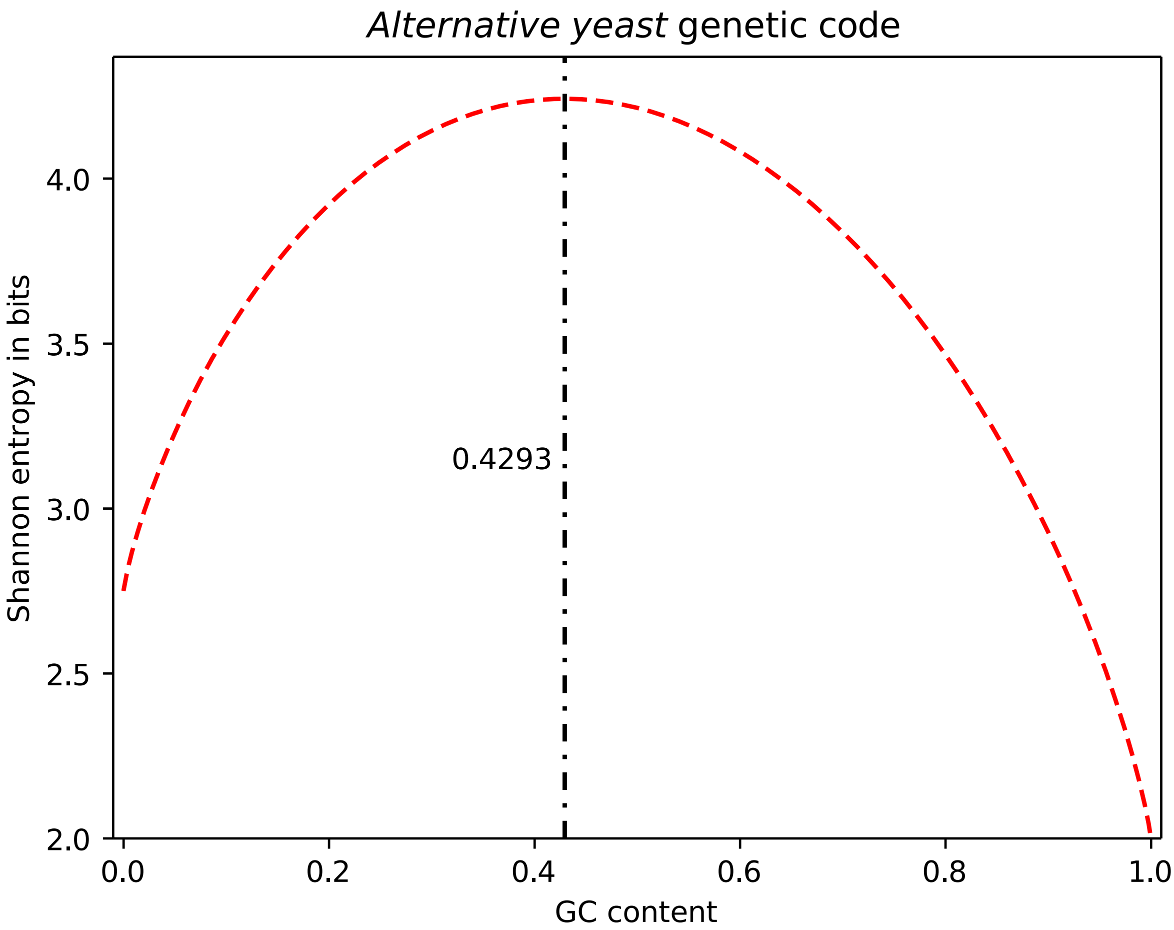

Figure S6. Entropy of the *alternative yeast* genetic code for its given codon assignments and GC contents between 0 % and 100 % as calculated by Shannon's entropy equation. The dash-dotted line indicates the GC content (42.93 %) at which this code reaches its entropy maximum (4.24 bits).

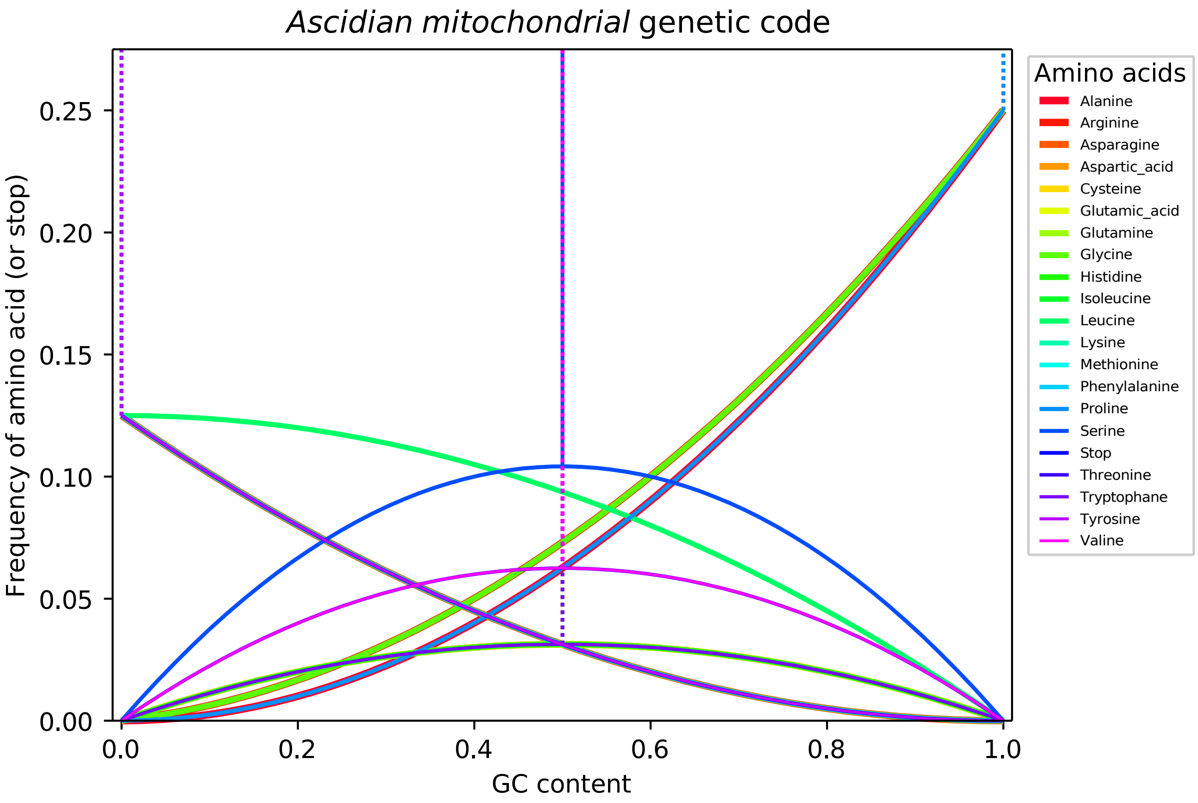

Figure S7. Frequencies of all amino acids (including stop) as encoded by the *ascidian mitochondrial* genetic code in random sequences as a function of GC content between 0 % and 100 %. The dashed lines mark the maximum achieved frequency for each amino acid (including stop).

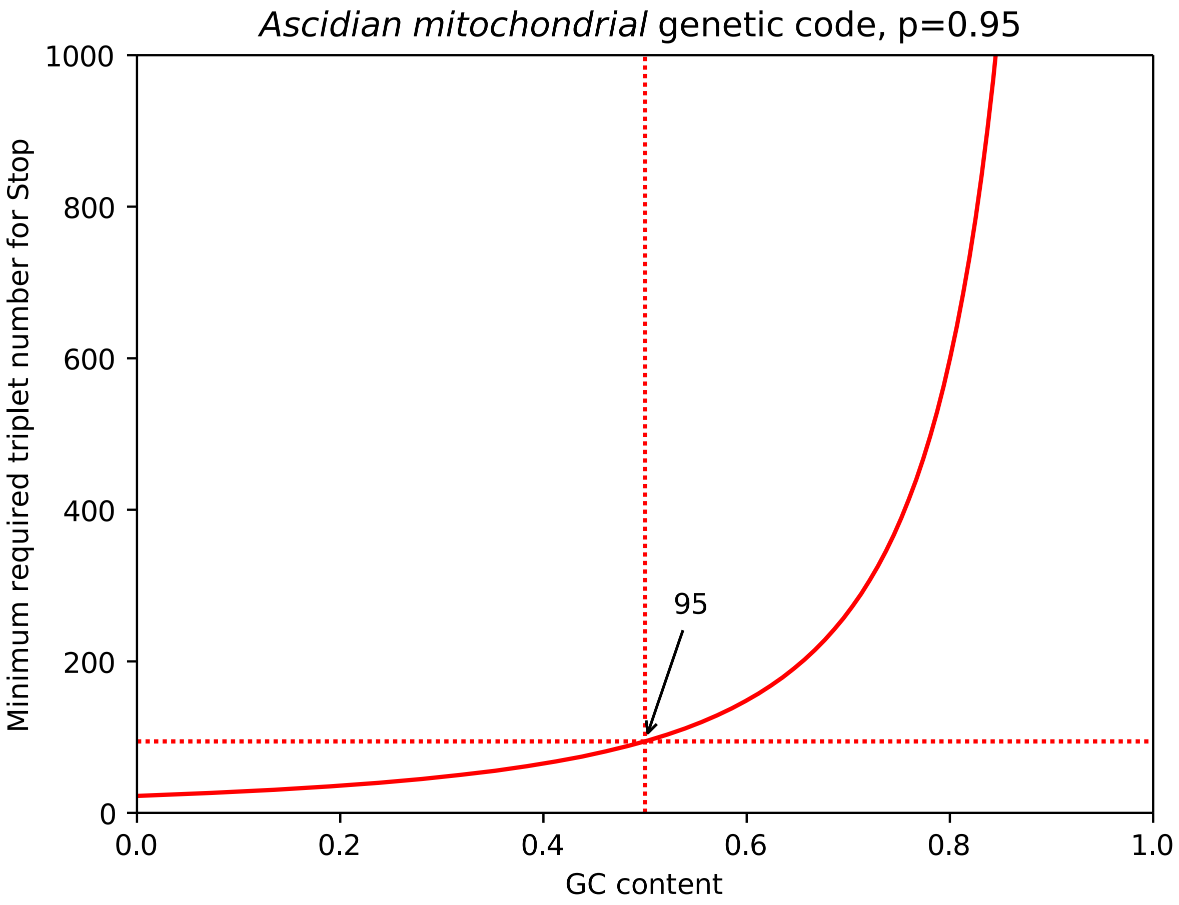

Figure S8. Number of triplets in a random sequence so as to contain at least one stop codon with a probability of 95 % using the *ascidian mitochondrial* genetic code as a function of GC content. The horizontal and vertical dashed lines indicate the number of triplets for a GC content of 50 % (95 triplets).

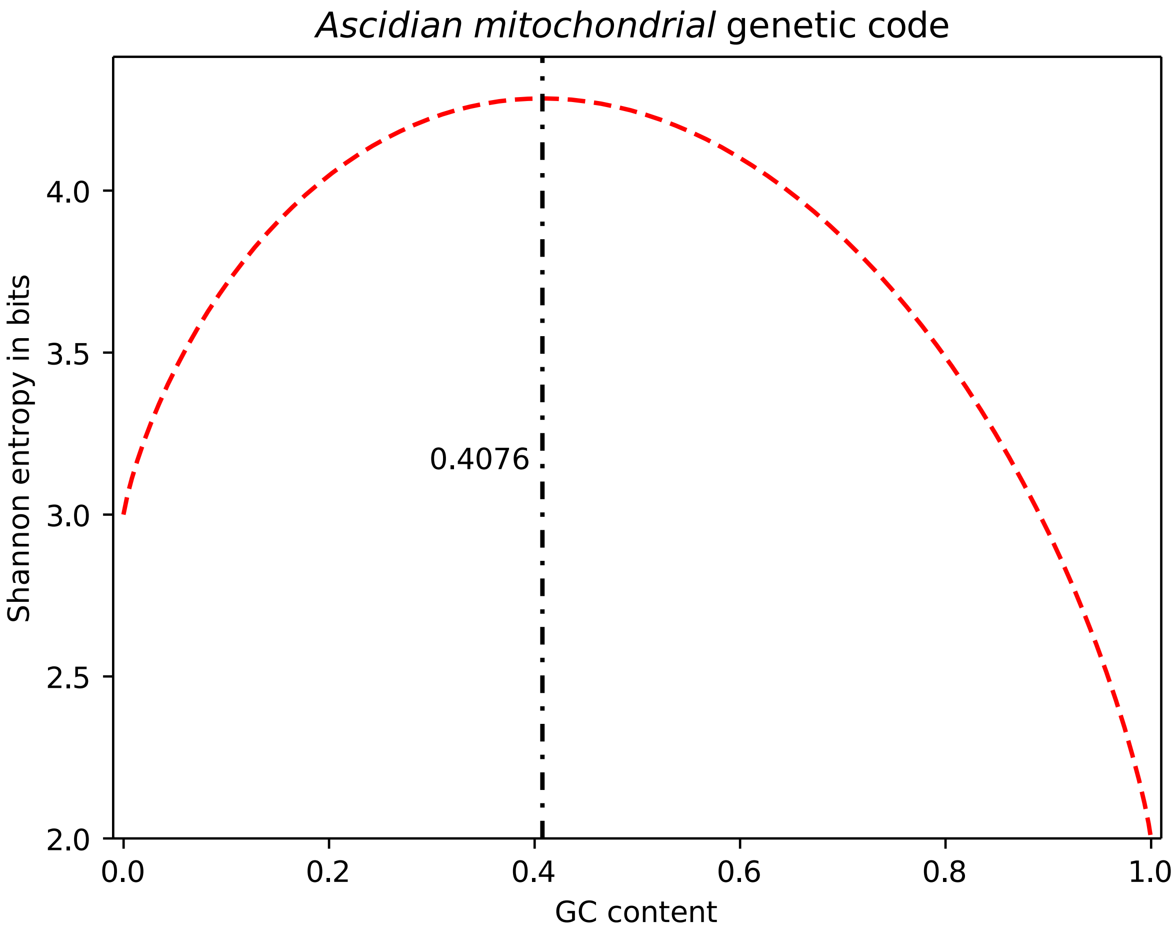

Figure S9. Entropy of the *ascidian mitochondrial* genetic code for its given codon assignments and GC contents between 0 % and 100 % as calculated by Shannon's entropy equation. The dash-dotted line indicates the GC content (40.76 %) at which this code reaches its entropy maximum (4.28 bits).

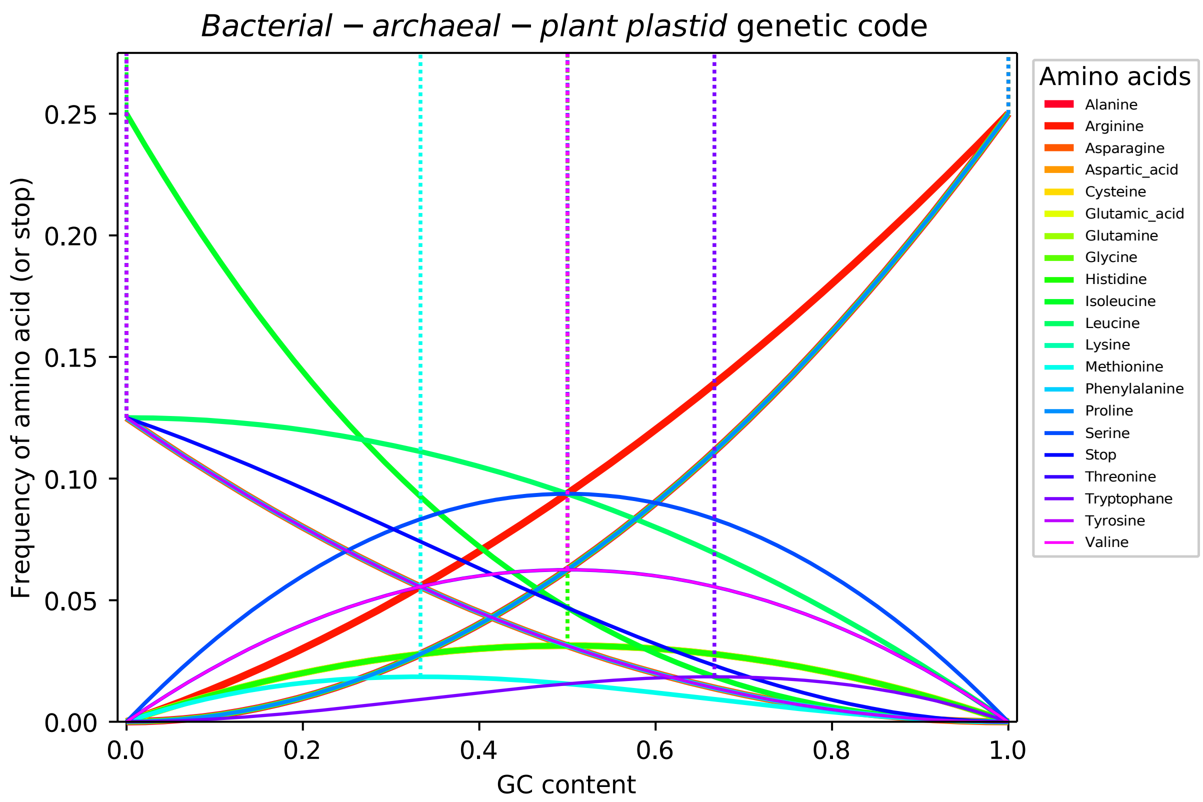

Figure S10. Frequencies of all amino acids (including stop) as encoded by the *bacteria – archaeal – plant plastid* genetic code in random sequences as a function of GC content between 0 % and 100 %. The dashed lines mark the maximum achieved frequency for each amino acid (including stop).

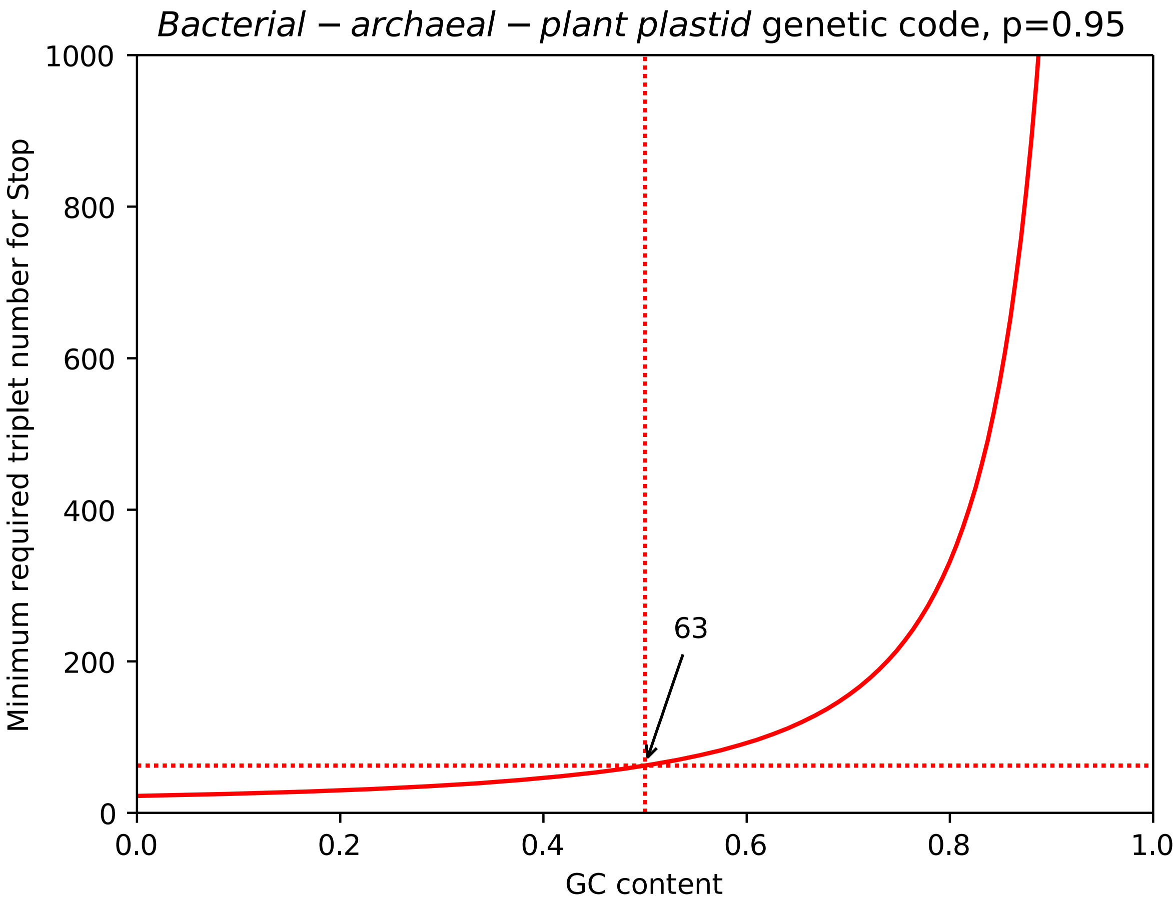

Figure S11. Number of triplets in a random sequence so as to contain at least one stop codon with a probability of 95 % using the *bacteria – archaeal – plant plastid* genetic code as a function of GC content. The horizontal and vertical dashed lines indicate the number of triplets for a GC content of 50 % (63 triplets).

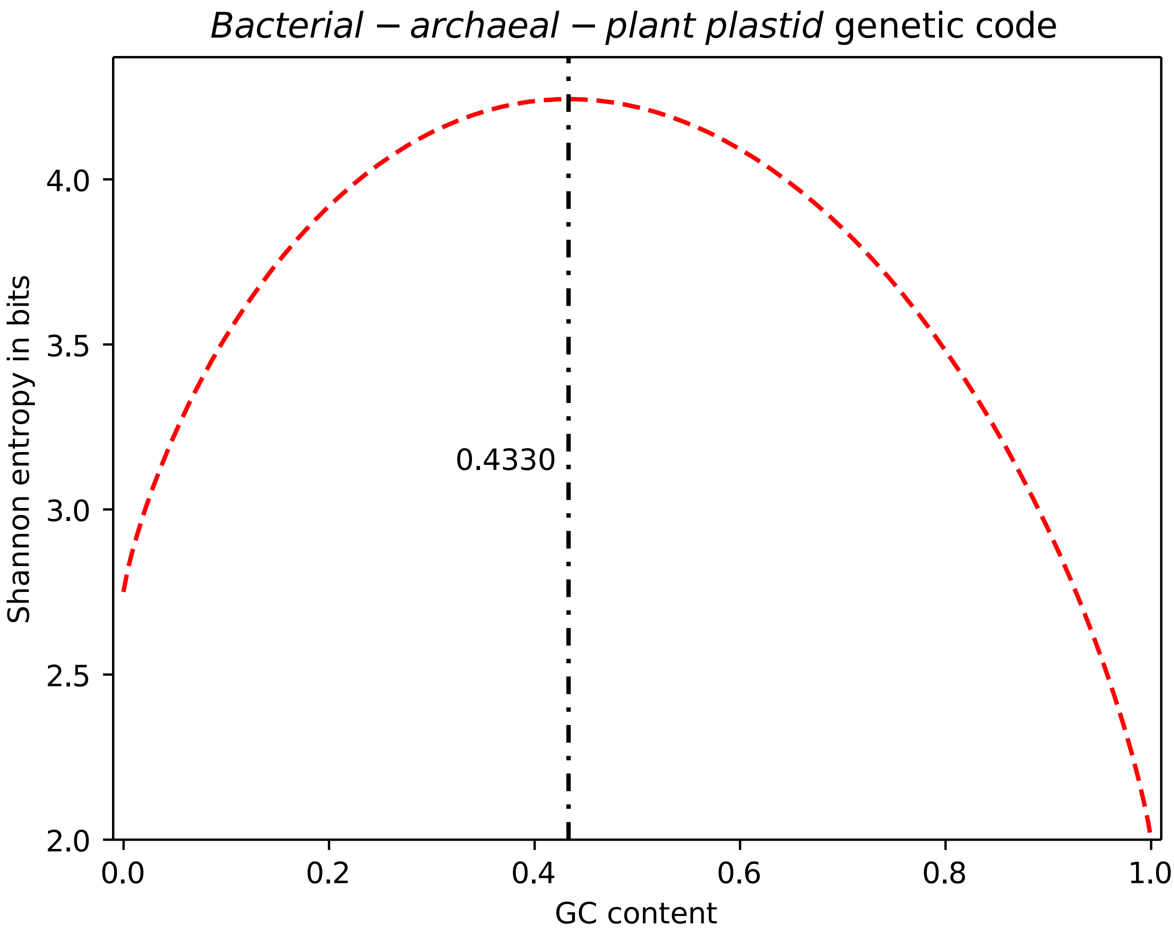

Figure S12. Entropy of the *bacteria – archaeal – plant plastid* genetic code for its given codon assignments and GC contents between 0 % and 100 % as calculated by Shannon's entropy equation. The dash-dotted line indicates the GC content (43.30 %) at which this code reaches its entropy maximum (4.24 bits).

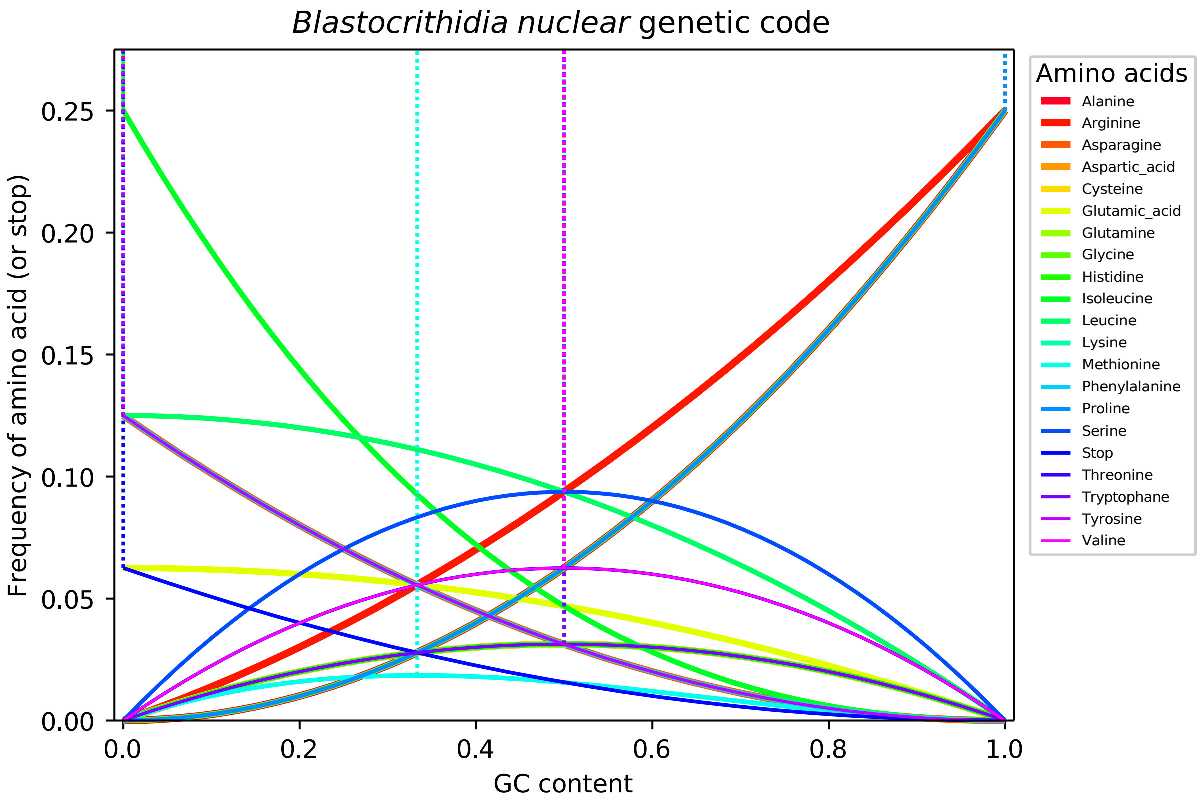

Figure S13. Frequencies of all amino acids (including stop) as encoded by the *Blastocrithidia nuclear* genetic code in random sequences as a function of GC content between 0 % and 100 %. The dashed lines mark the maximum achieved frequency for each amino acid (including stop).

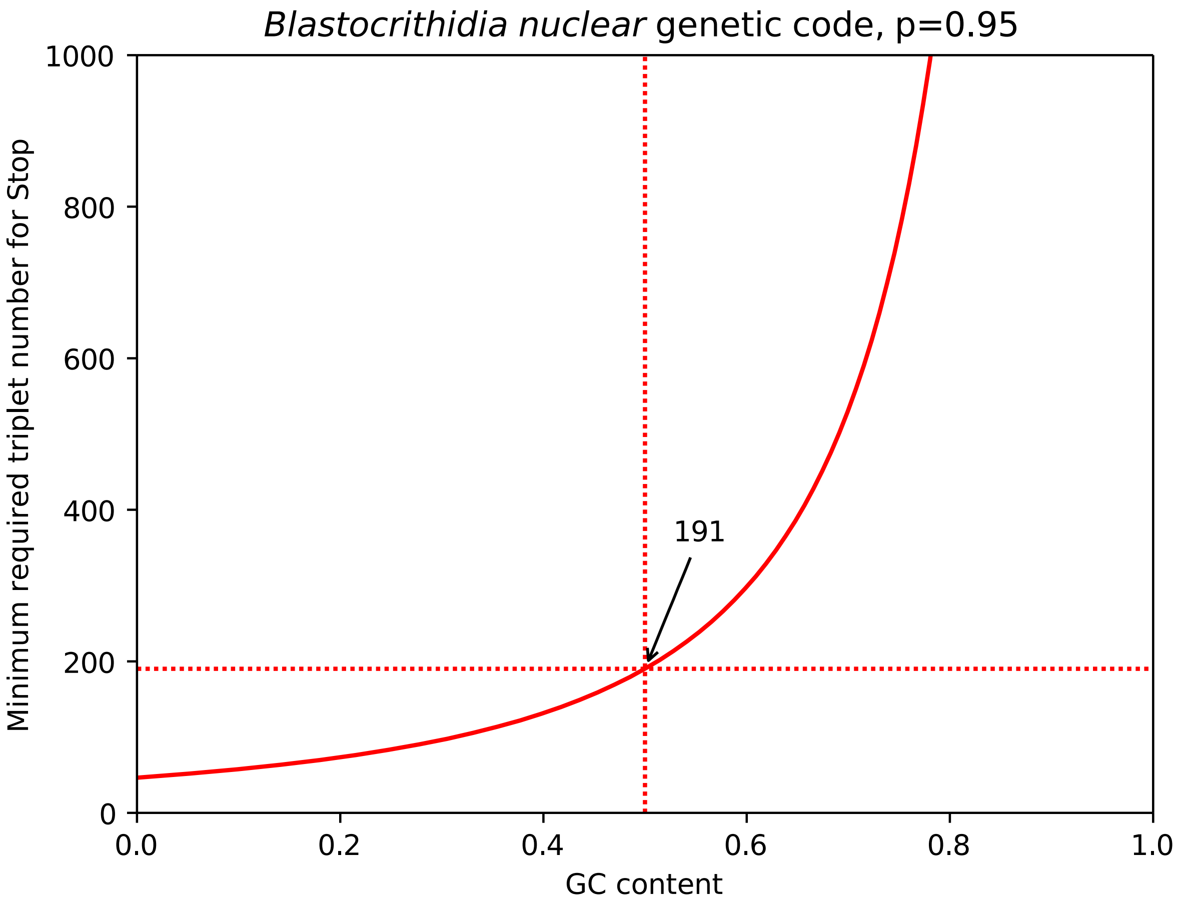

Figure S14. Number of triplets in a random sequence so as to contain at least one stop codon with a probability of 95 % using the *Blastocrithidia nuclear* genetic code as a function of GC content. The horizontal and vertical dashed lines indicate the number of triplets for a GC content of 50 % (191 triplets).

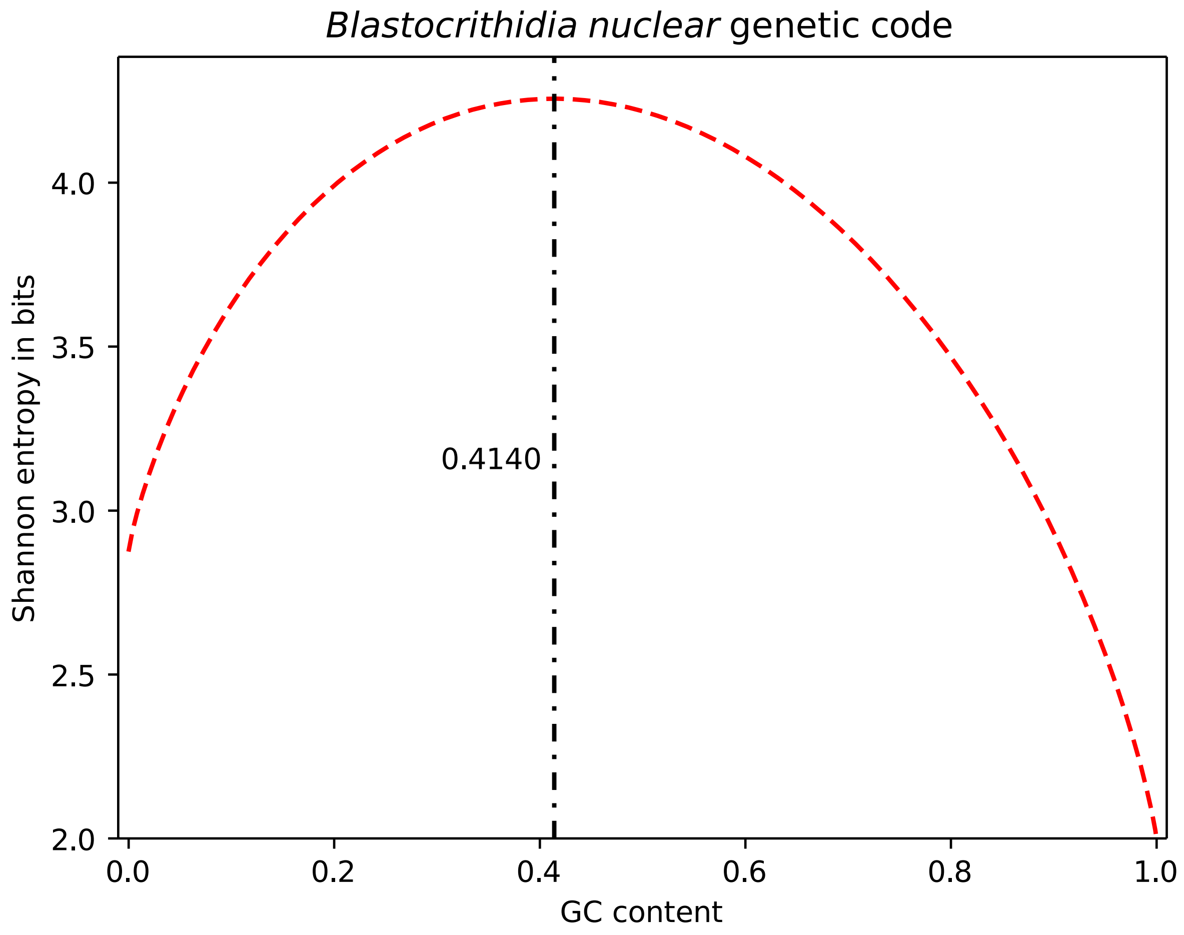

Figure S15. Entropy of the *Blastocrithidia nuclear* genetic code for its given codon assignments and GC contents between 0 % and 100 % as calculated by Shannon's entropy equation. The dash-dotted line indicates the GC content (41.40 %) at which this code reaches its entropy maximum (4.26 bits).

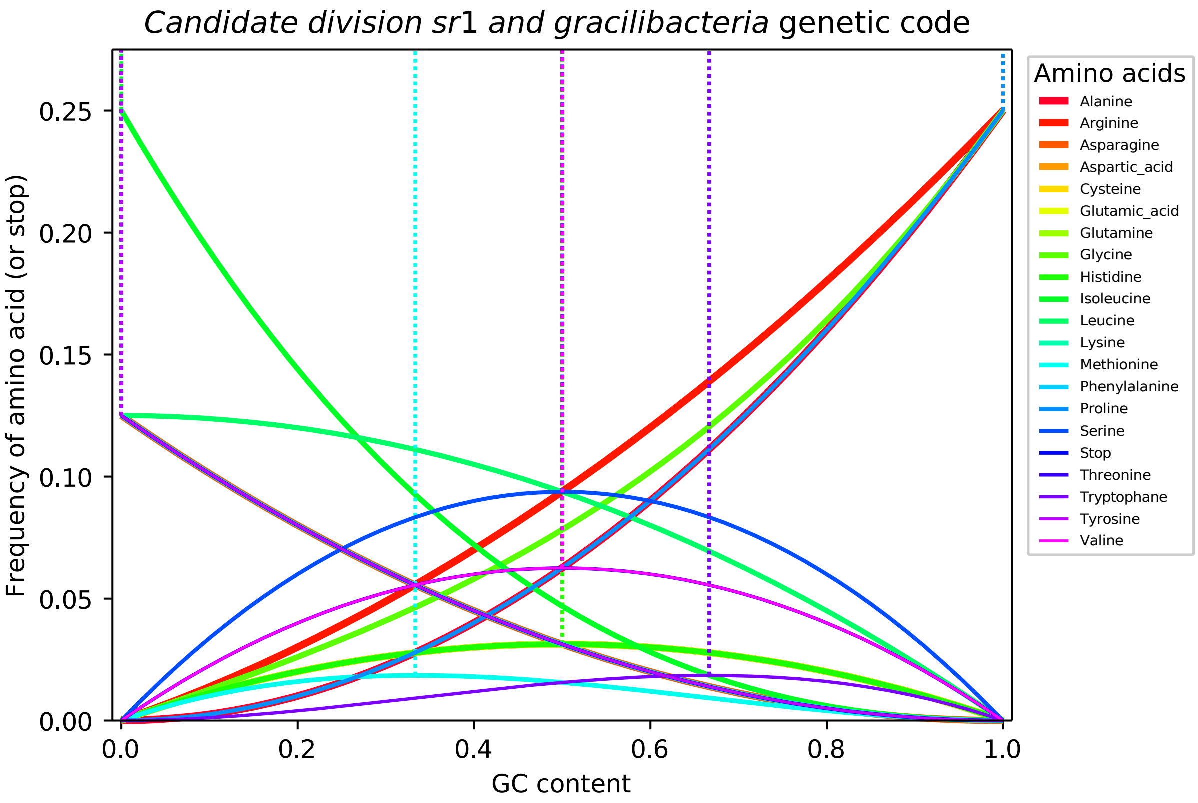

Figure S16. Frequencies of all amino acids (including stop) as encoded by the *candidate division sr1 and Gracilibacteria* genetic code in random sequences as a function of GC content between 0 % and 100 %. The dashed lines mark the maximum achieved frequency for each amino acid (including stop).

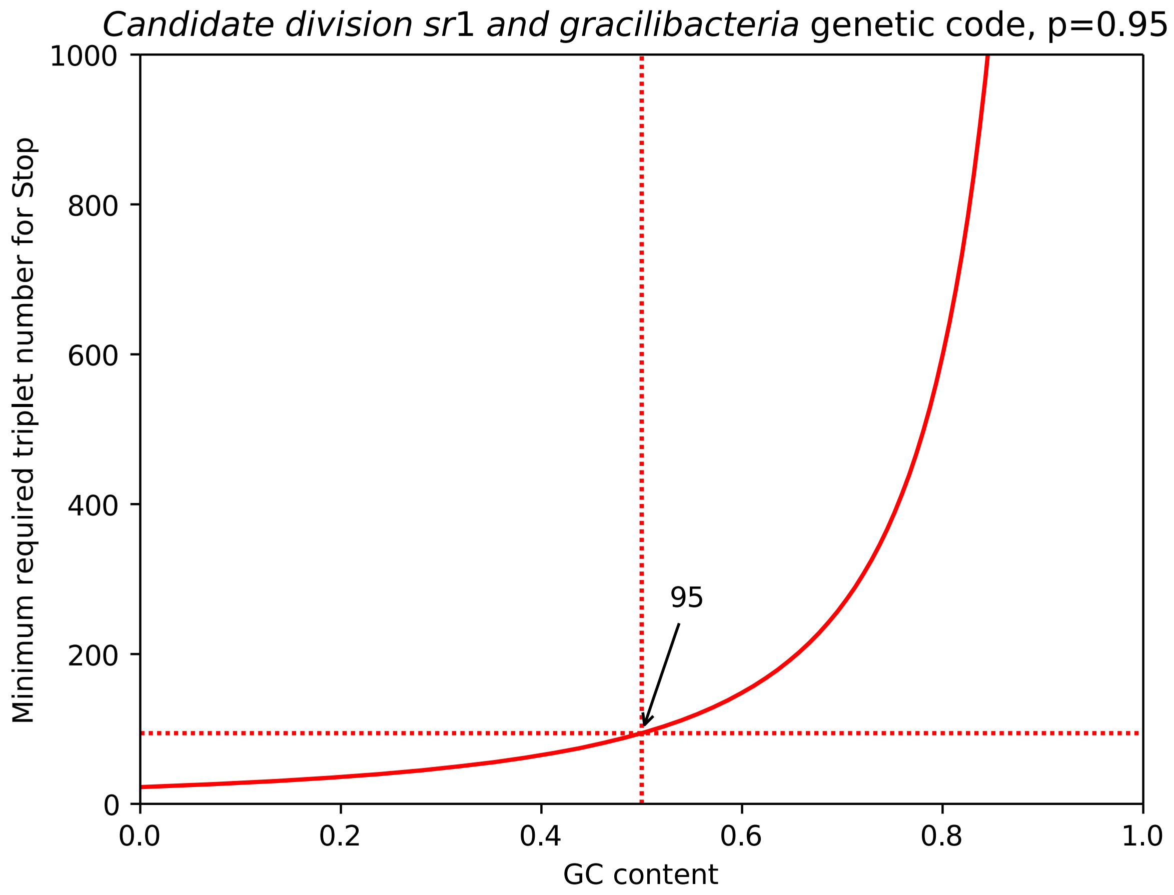

Figure S17. Number of triplets in a random sequence so as to contain at least one stop codon with a probability of 95 % using the *candidate division sr1 and Gracilibacteria* genetic code as a function of GC content. The horizontal and vertical dashed lines indicate the number of triplets for a GC content of 50 % (95 triplets).

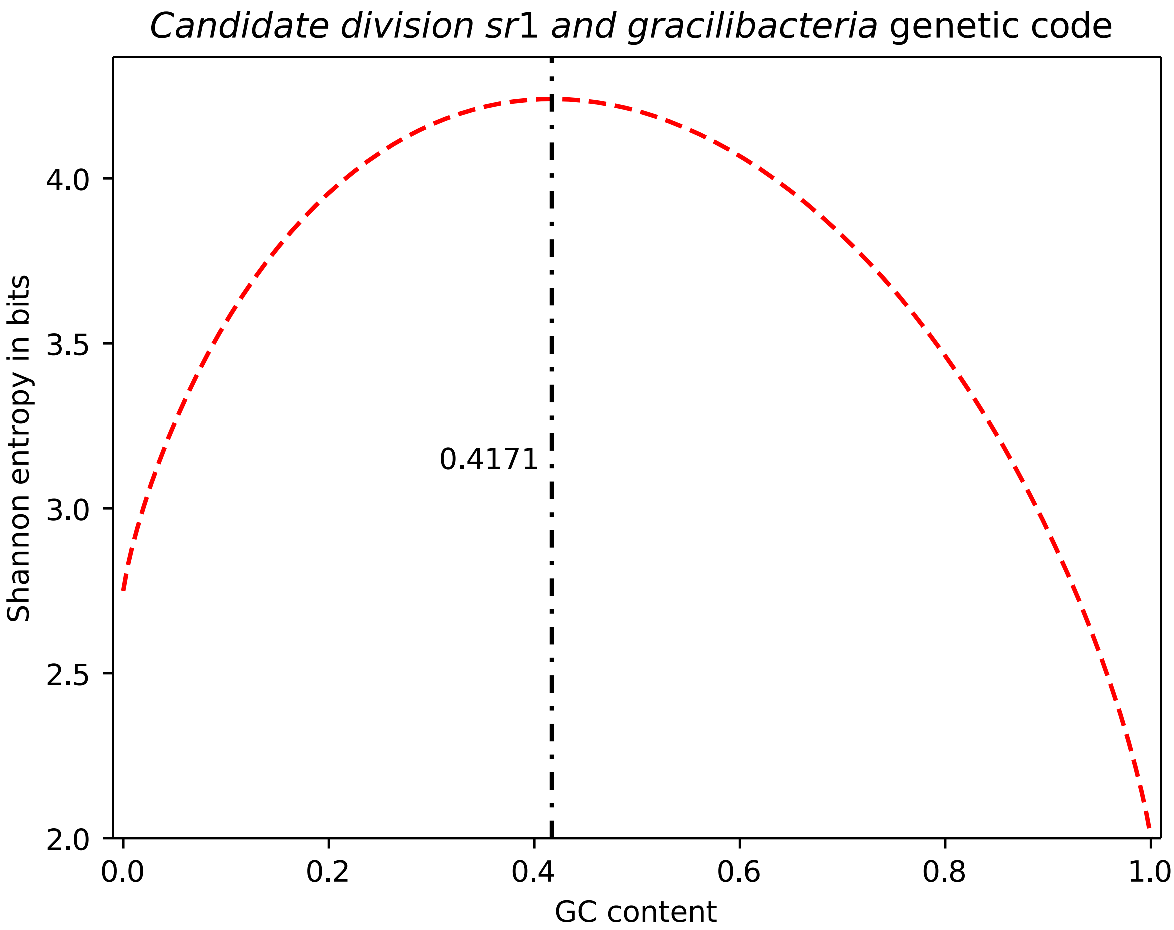

Figure S18. Entropy of the *candidate division sr1 and Gracilibacteria* genetic code for its given codon assignments and GC contents between 0 % and 100 % as calculated by Shannon's entropy equation. The dash-dotted line indicates the GC content (41.71 %) at which this code reaches its entropy maximum (4.24 bits).

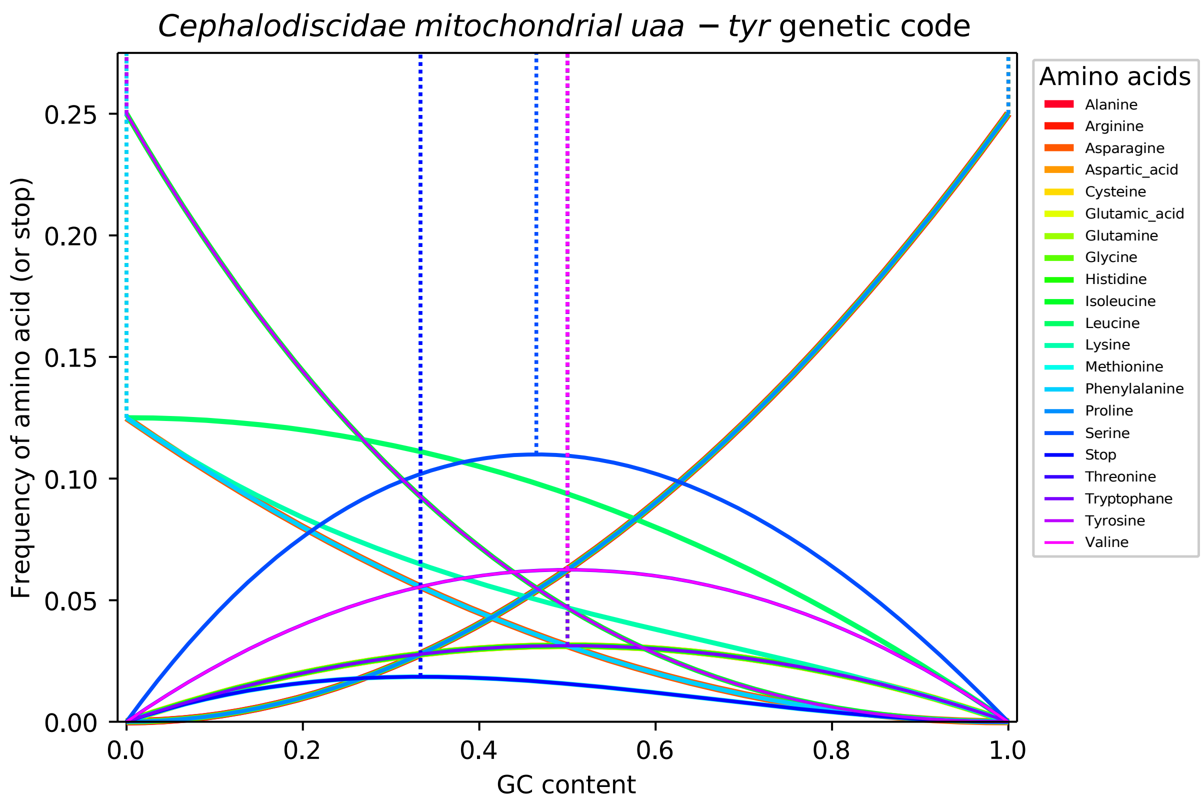

Figure S19. Frequencies of all amino acids (including stop) as encoded by the *Cephalodiscidae mitochondrial uaa – tyr* genetic code in random sequences as a function of GC content between 0 % and 100 %. The dashed lines mark the maximum achieved frequency for each amino acid (including stop).

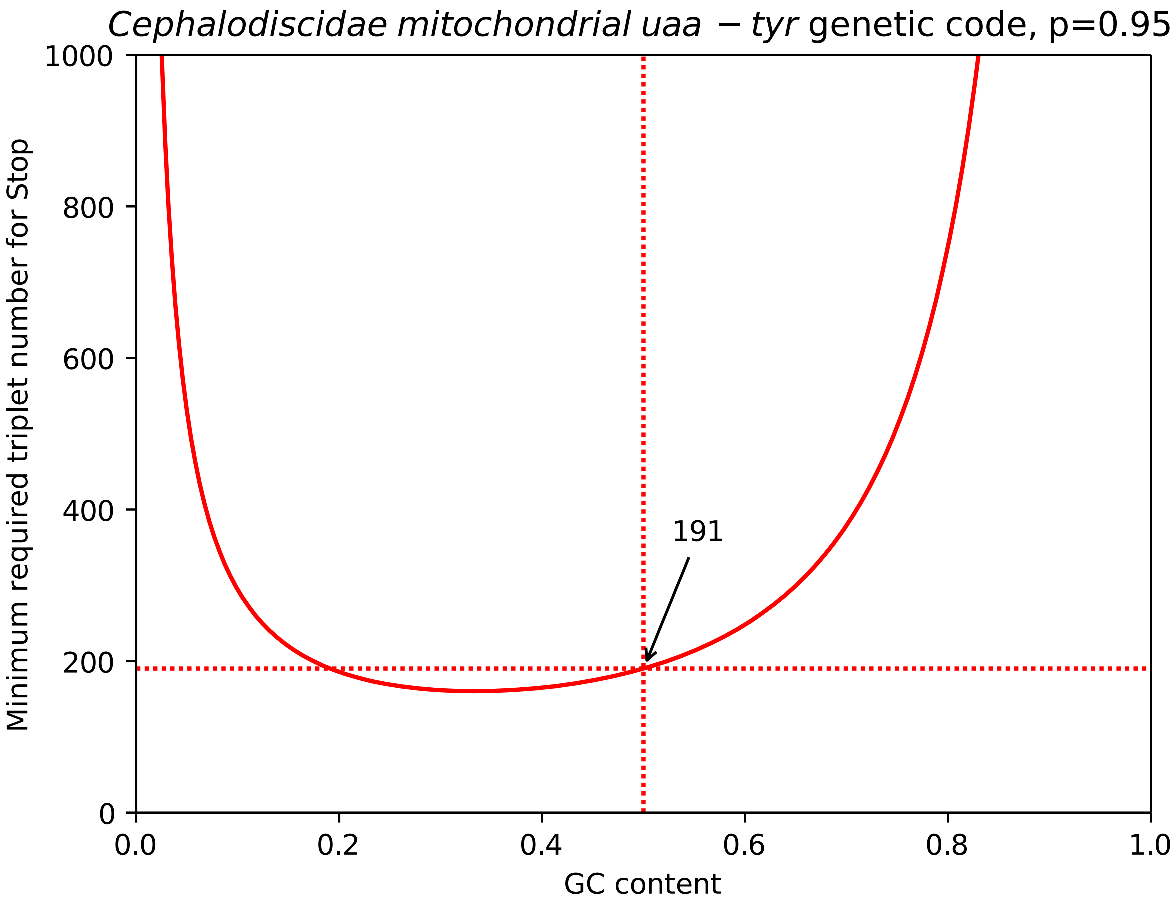

Figure S20. Number of triplets in a random sequence so as to contain at least one stop codon with a probability of 95 % using the *Cephalodiscidae mitochondrial uaa – tyr* genetic code as a function of GC content. The horizontal and vertical dashed lines indicate the number of triplets for a GC content of 50 % (191 triplets).

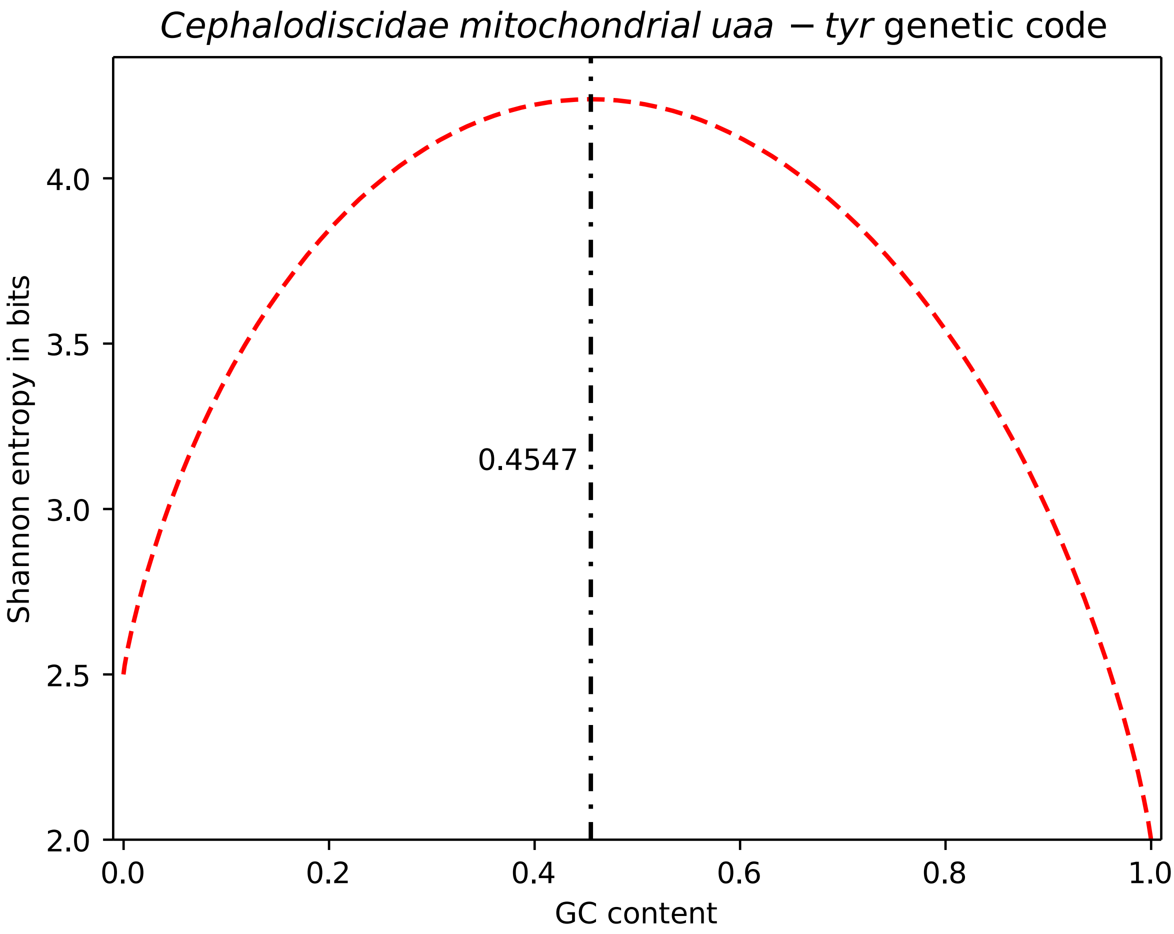

Figure S21. Entropy of the *Cephalodiscidae mitochondrial uaa – tyr* genetic code for its given codon assignments and GC contents between 0 % and 100 % as calculated by Shannon's entropy equation. The dash-dotted line indicates the GC content (45.47 %) at which this code reaches its entropy maximum (4.24 bits).

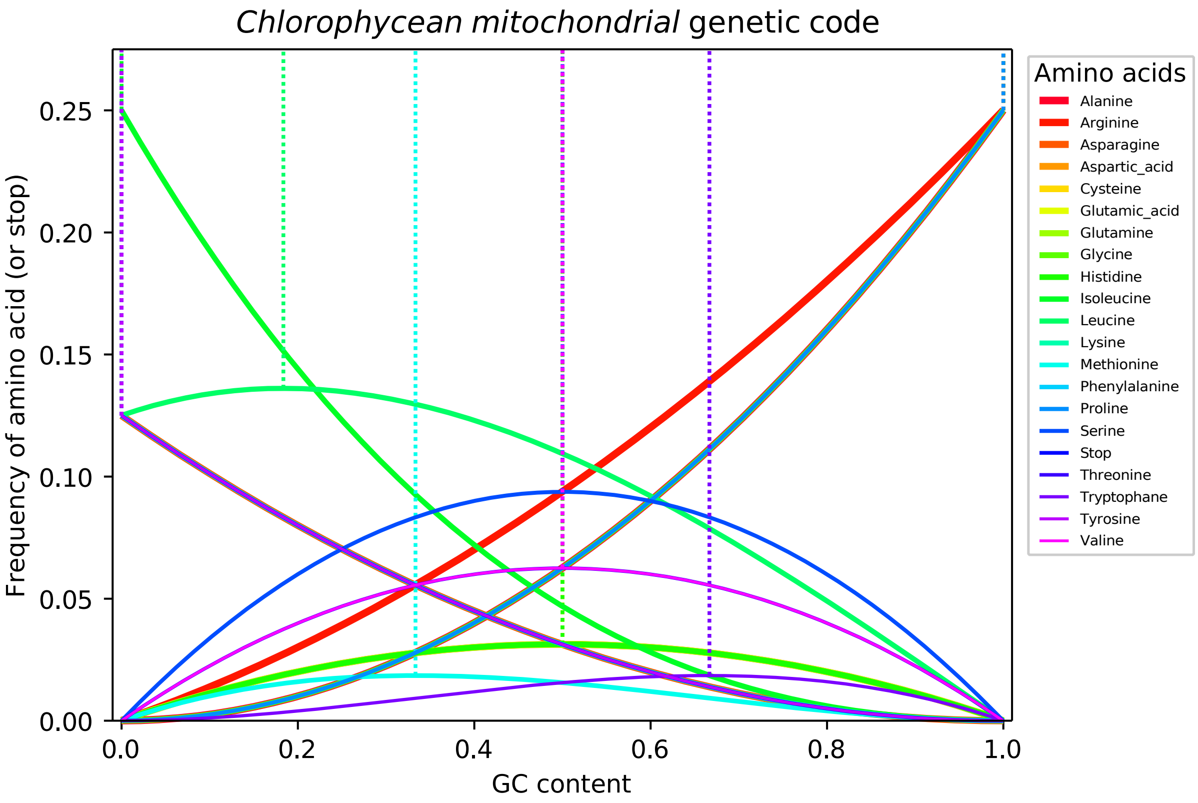

Figure S22. Frequencies of all amino acids (including stop) as encoded by the *Chlorophycean mitochondrial* genetic code in random sequences as a function of GC content between 0 % and 100 %. The dashed lines mark the maximum achieved frequency for each amino acid (including stop).

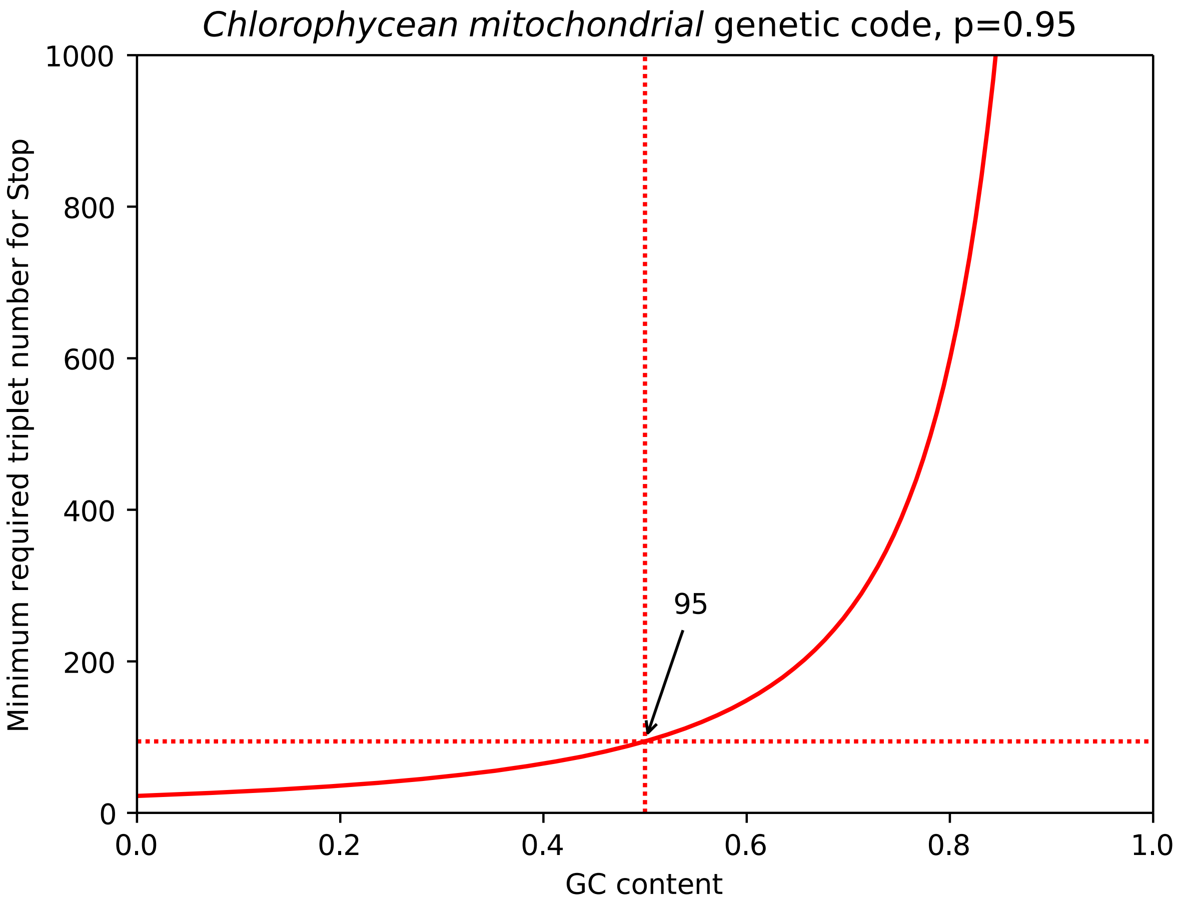

Figure S23. Number of triplets in a random sequence so as to contain at least one stop codon with a probability of 95 % using the *Chlorophycean mitochondrial* genetic code as a function of GC content. The horizontal and vertical dashed lines indicate the number of triplets for a GC content of 50 % (95 triplets).

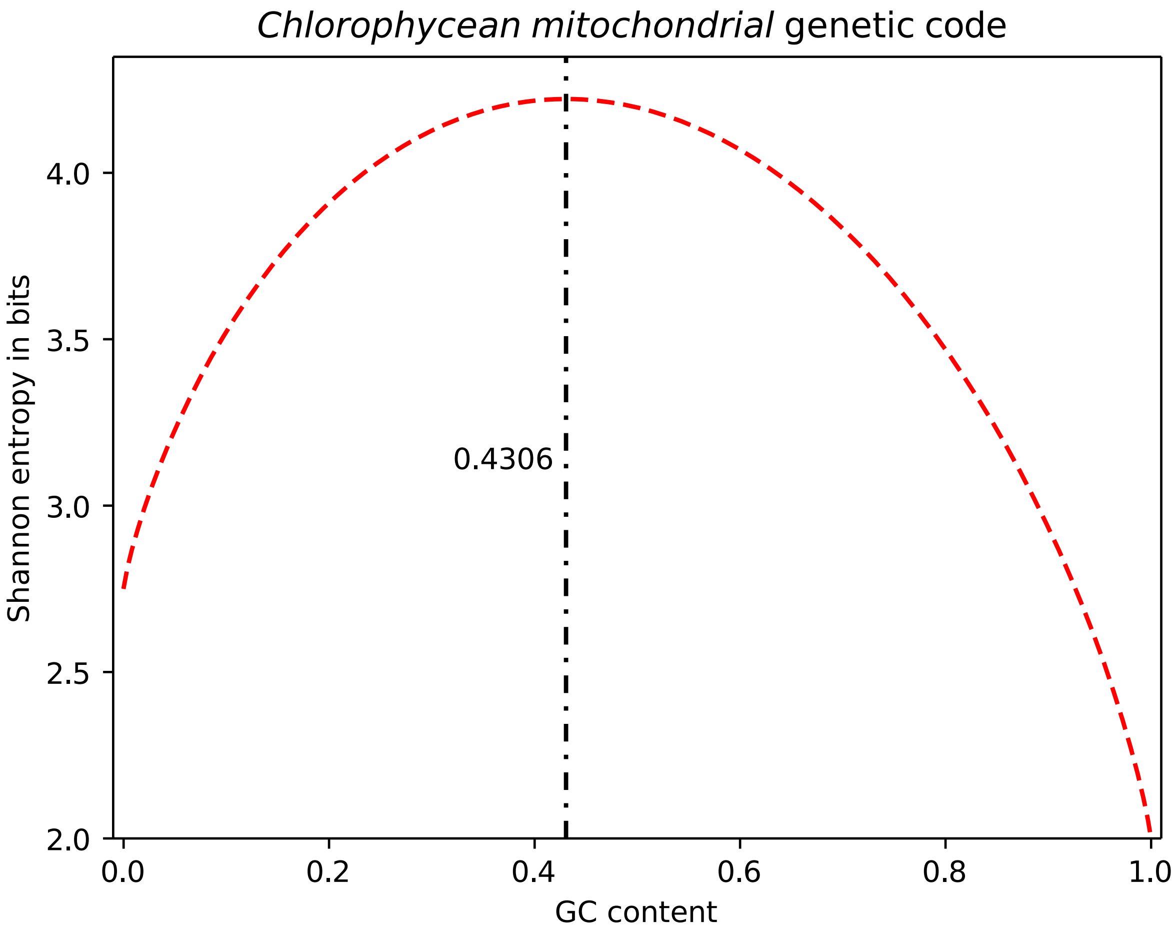

Figure S24. Entropy of the *Chlorophycean mitochondrial* genetic code for its given codon assignments and GC contents between 0 % and 100 % as calculated by Shannon's entropy equation. The dash-dotted line indicates the GC content (45.61 %) at which this code reaches its entropy maximum (4.22 bits).

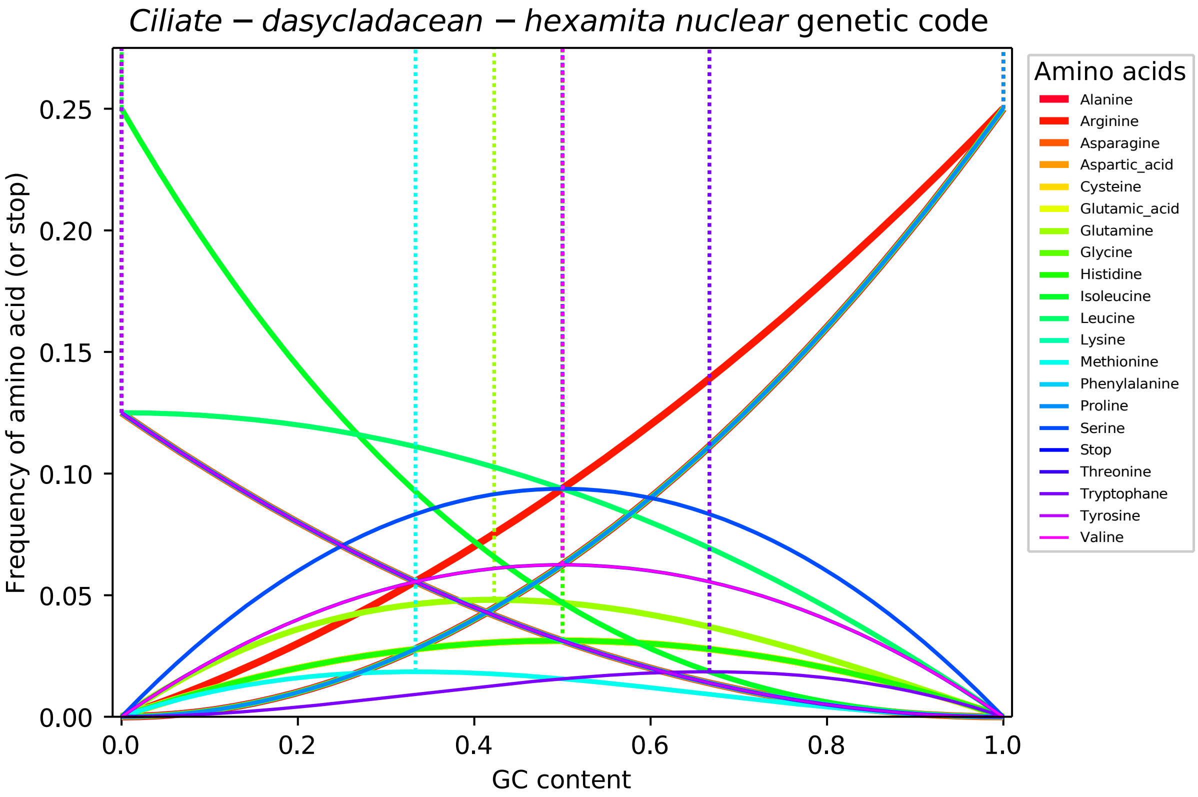

Figure S25. Frequencies of all amino acids (including stop) as encoded by the *Ciliate – Dasycladacean – Hexamita nuclear* genetic code in random sequences as a function of GC content between 0 % and 100 %. The dashed lines mark the maximum achieved frequency for each amino acid (including stop).

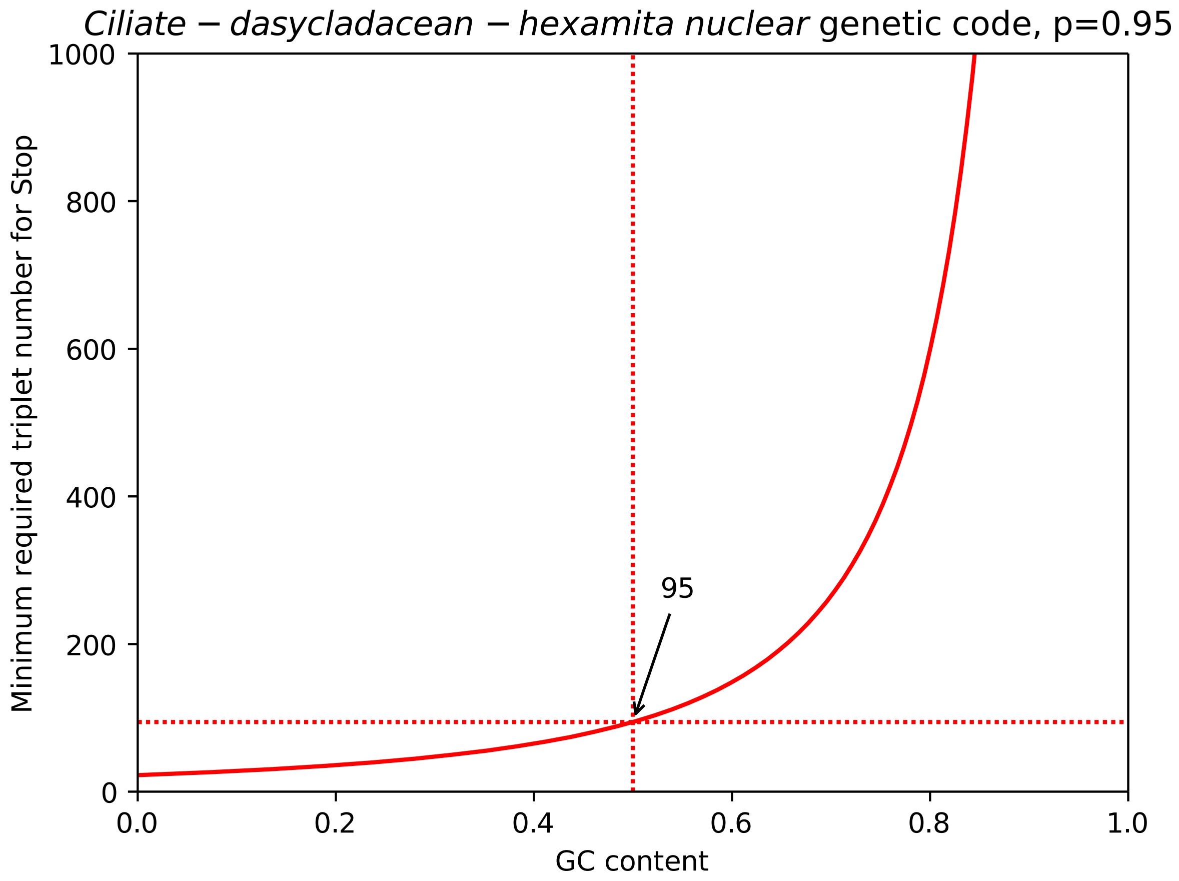

Figure S26. Number of triplets in a random sequence so as to contain at least one stop codon with a probability of 95 % using the *Ciliate – Dasycladacean – Hexamita nuclear* genetic code as a function of GC content. The horizontal and vertical dashed lines indicate the number of triplets for a GC content of 50 % (95 triplets).

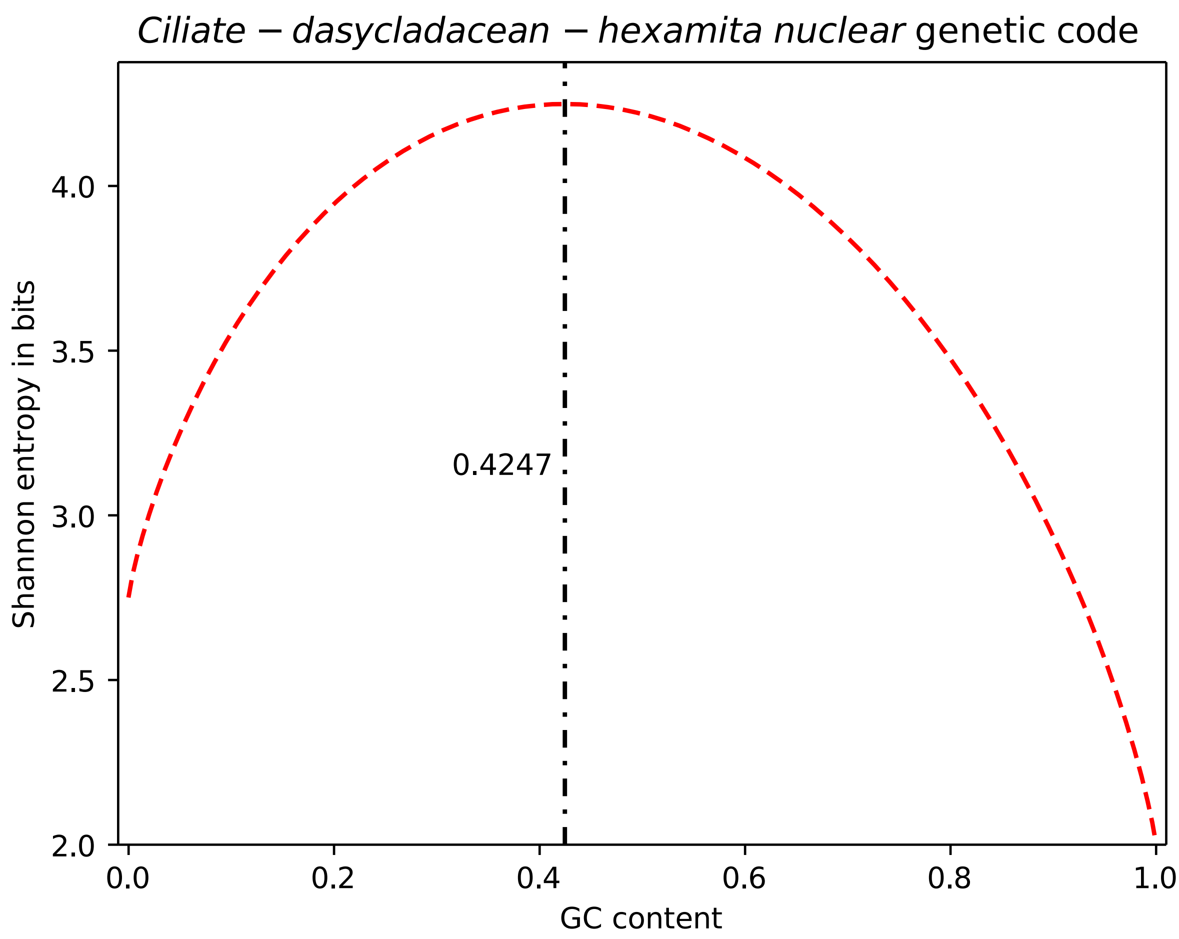

Figure S27. Entropy of the *Ciliate – Dasycladacean – Hexamita nuclear* genetic code for its given codon assignments and GC contents between 0 % and 100 % as calculated by Shannon's entropy equation. The dash-dotted line indicates the GC content (42.47 %) at which this code reaches its entropy maximum (4.25 bits).

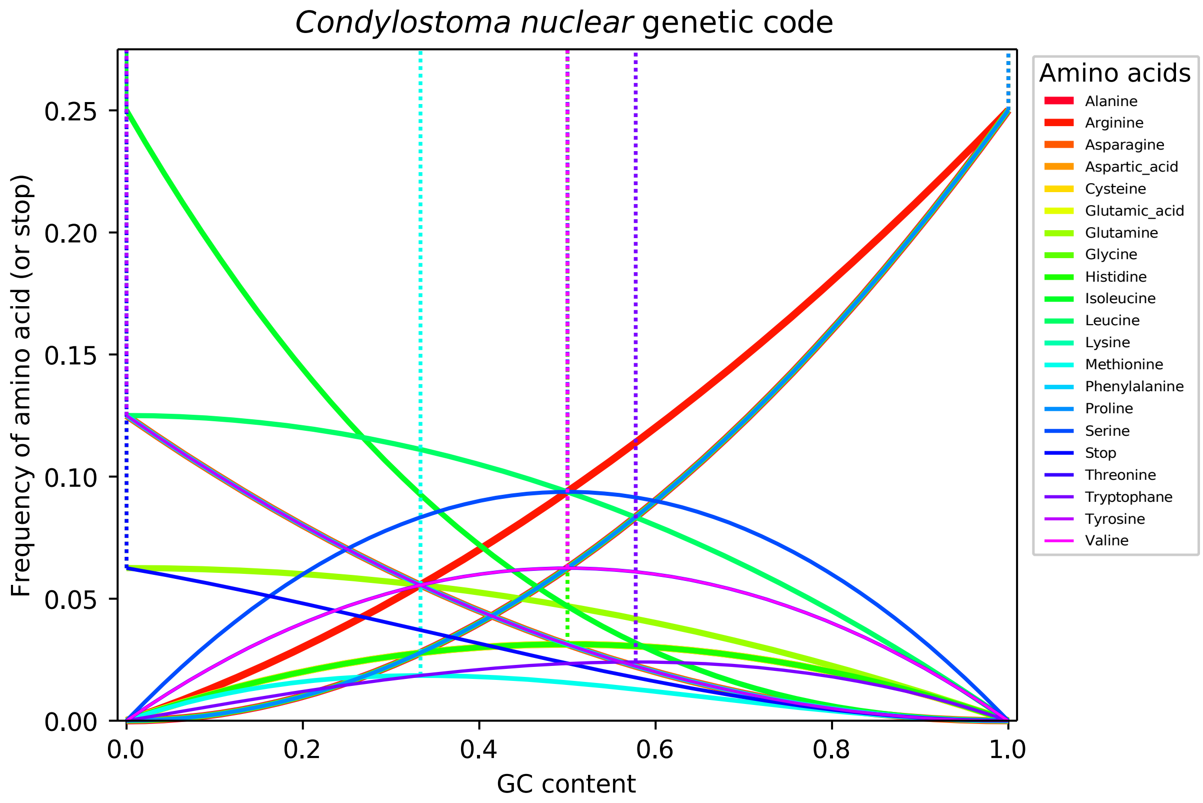

Figure S28. Frequencies of all amino acids (including stop) as encoded by the *Condylostoma nuclear* genetic code in random sequences as a function of GC content between 0 % and 100 %. The dashed lines mark the maximum achieved frequency for each amino acid (including stop).

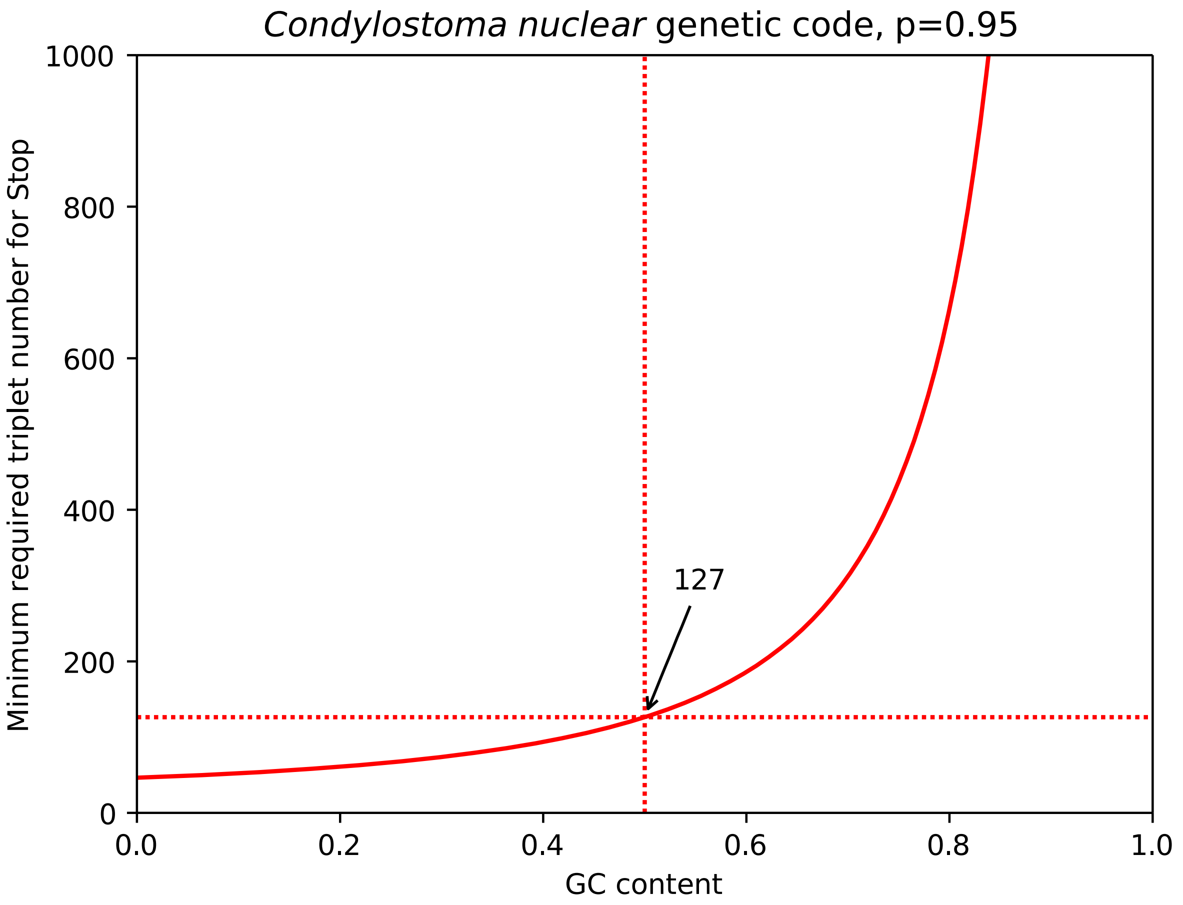

Figure S29. Number of triplets in a random sequence so as to contain at least one stop codon with a probability of 95 % using the *Condylostoma nuclear* genetic code as a function of GC content. The horizontal and vertical dashed lines indicate the number of triplets for a GC content of 50 % (127 triplets).

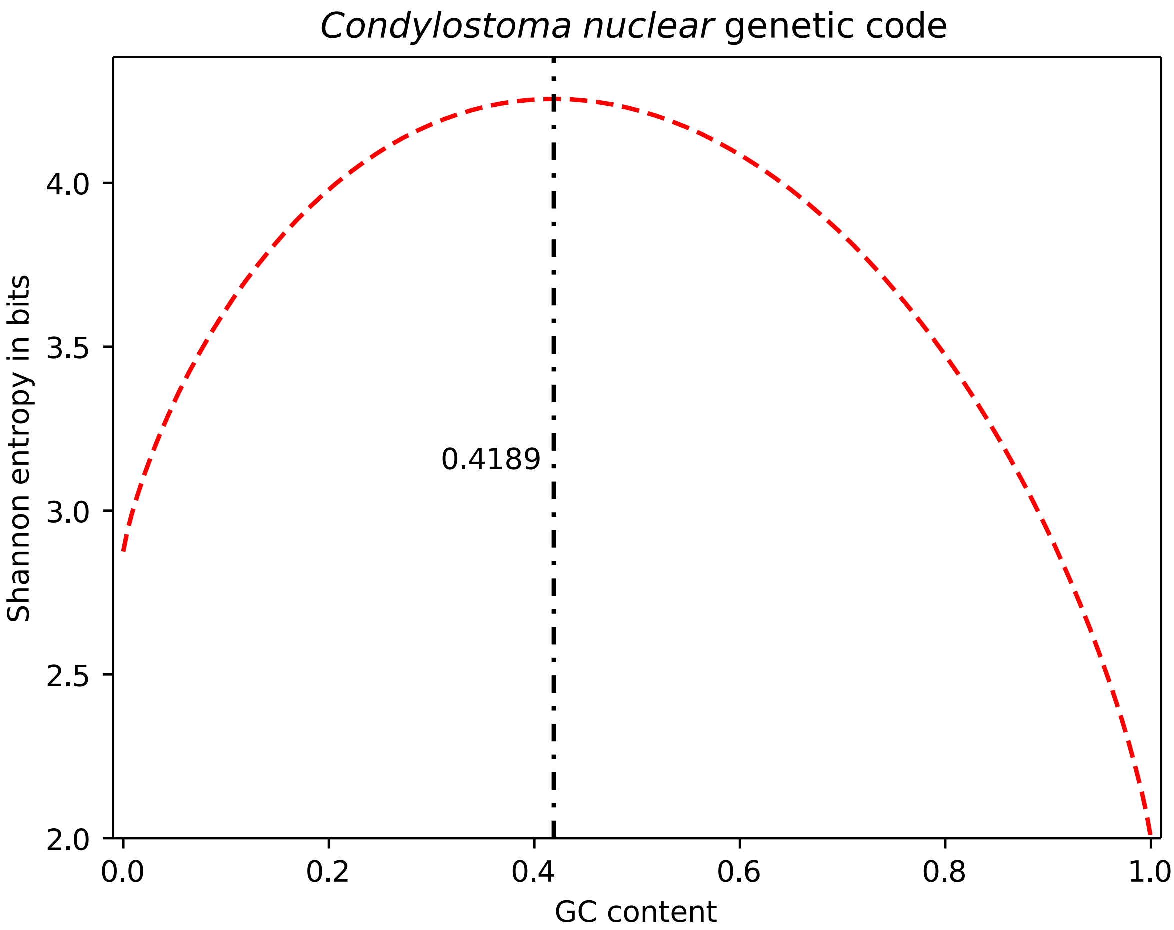

Figure S30. Entropy of the *Condylostoma nuclear* genetic code for its given codon assignments and GC contents between 0 % and 100 % as calculated by Shannon's entropy equation. The dash-dotted line indicates the GC content (41.89 %) at which this code reaches its entropy maximum (4.26 bits).

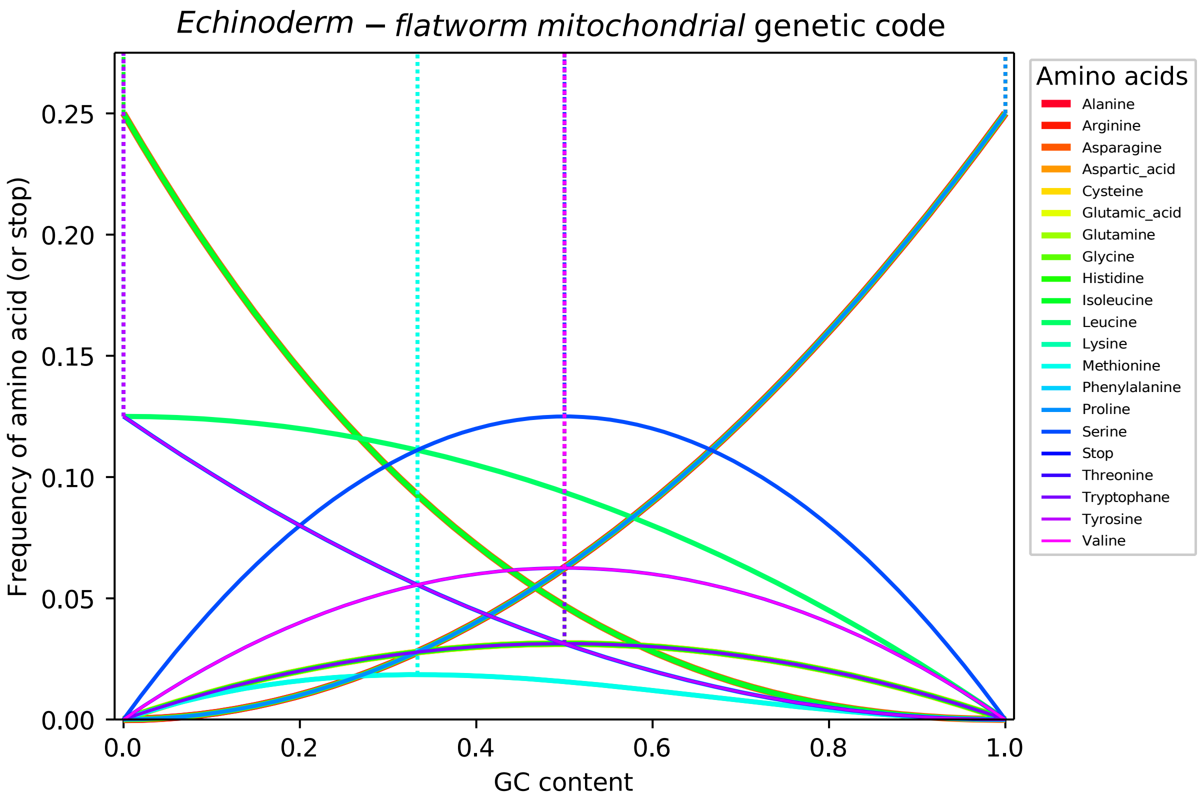

Figure S31. Frequencies of all amino acids (including stop) as encoded by the *echinoderm – flatworm mitochondrial* genetic code in random sequences as a function of GC content between 0 % and 100 %. The dashed lines mark the maximum achieved frequency for each amino acid (including stop).

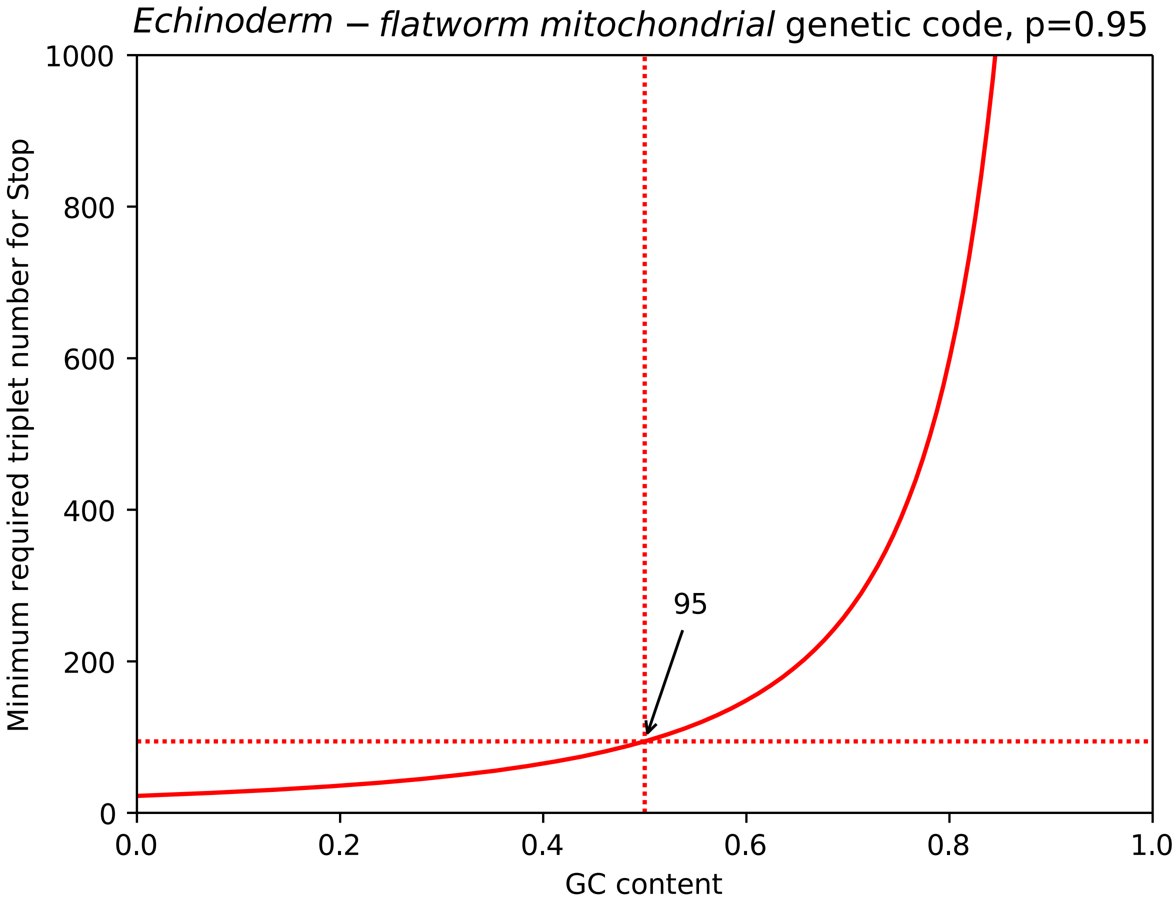

Figure S32. Number of triplets in a random sequence so as to contain at least one stop codon with a probability of 95 % using the *echinoderm – flatworm mitochondrial* genetic code as a function of GC content. The horizontal and vertical dashed lines indicate the number of triplets for a GC content of 50 % (95 triplets).

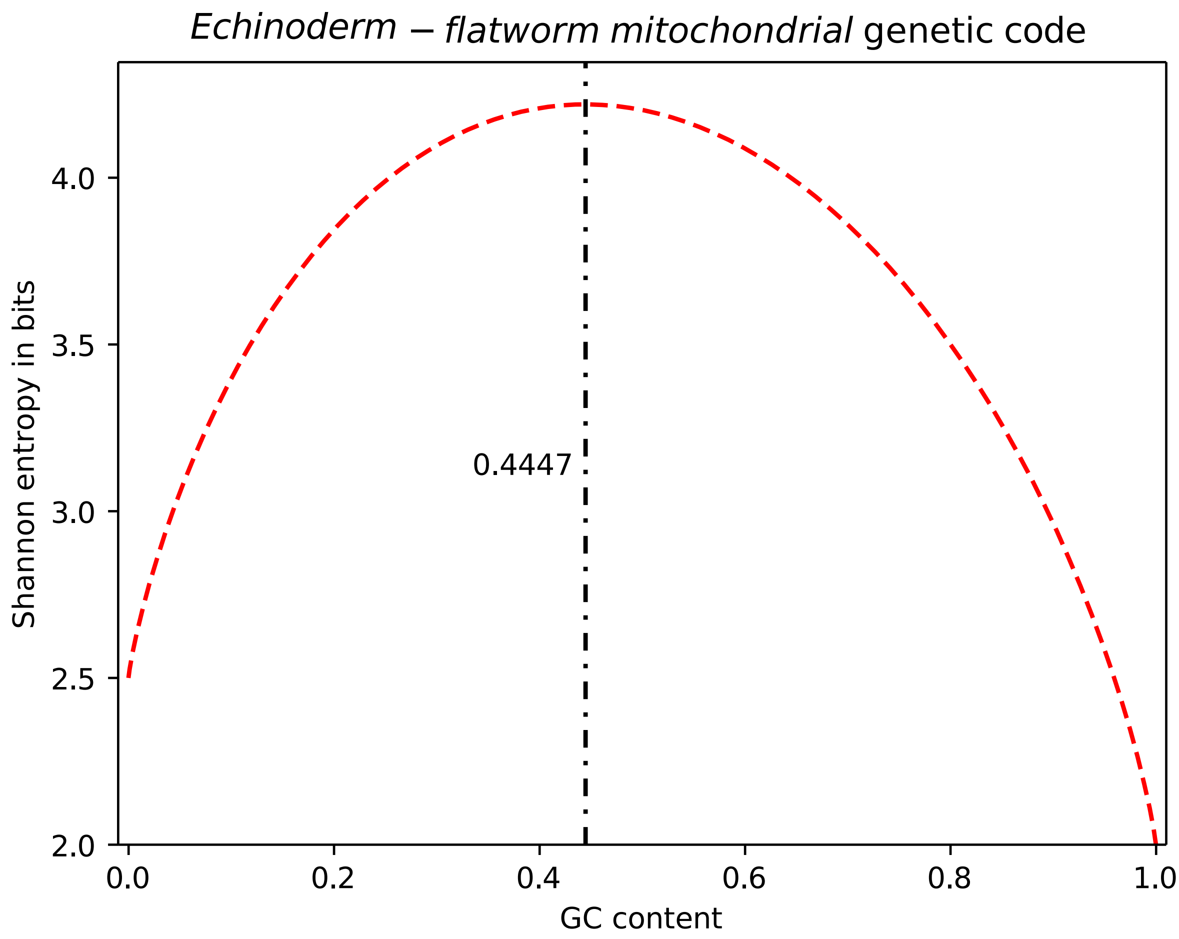

Figure S33. Entropy of the *echinoderm – flatworm mitochondrial* genetic code for its given codon assignments and GC contents between 0 % and 100 % as calculated by Shannon's entropy equation. The dash-dotted line indicates the GC content (44.47 %) at which this code reaches its entropy maximum (4.22 bits).

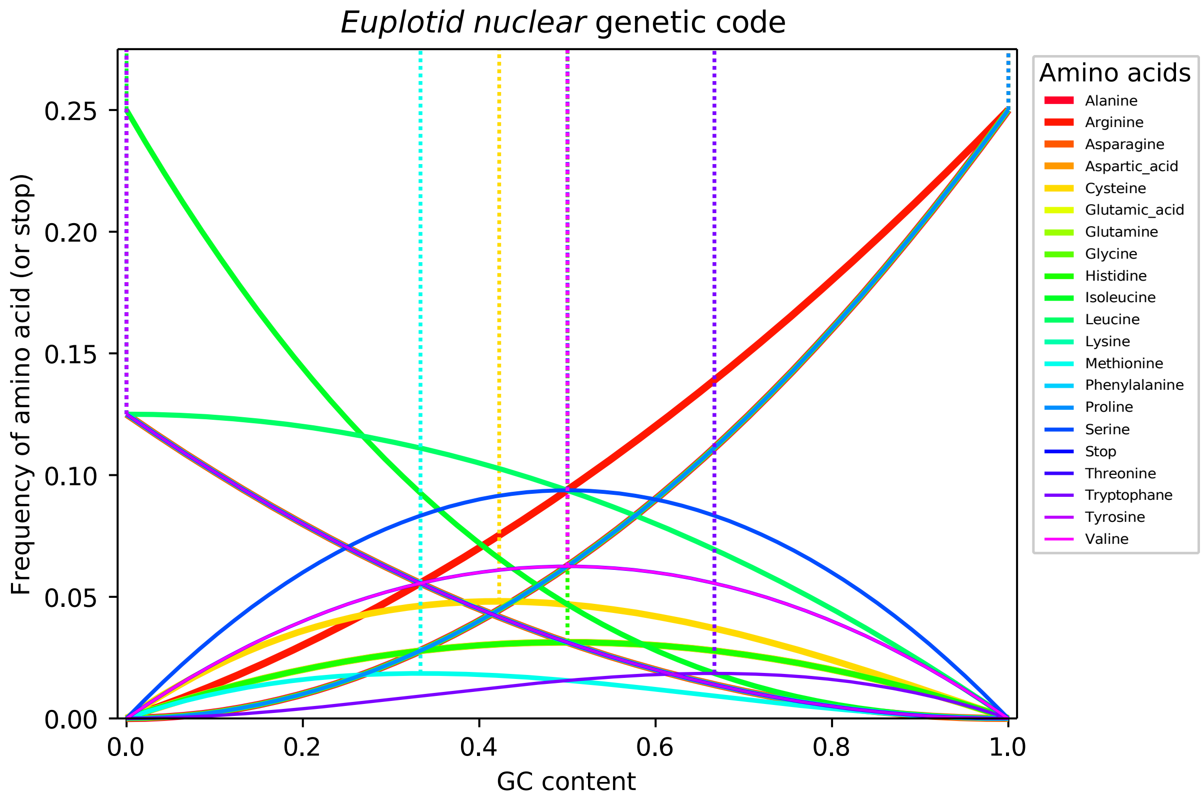

Figure S34. Frequencies of all amino acids (including stop) as encoded by the *Euplotid nuclear* genetic code in random sequences as a function of GC content between 0 % and 100 %. The dashed lines mark the maximum achieved frequency for each amino acid (including stop).

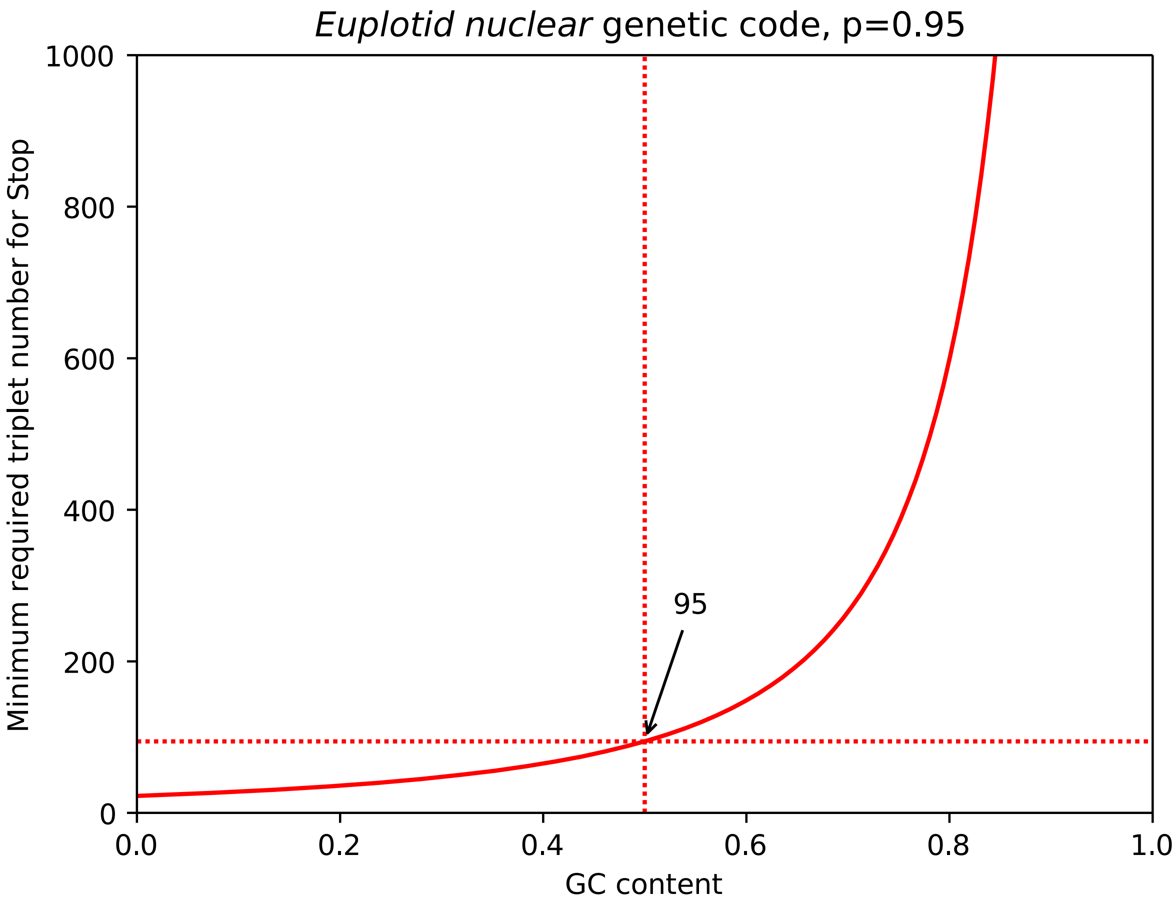

Figure S35. Number of triplets in a random sequence so as to contain at least one stop codon with a probability of 95 % using the *Euplotid nuclear* genetic code as a function of GC content. The horizontal and vertical dashed lines indicate the number of triplets for a GC content of 50 % (95 triplets).

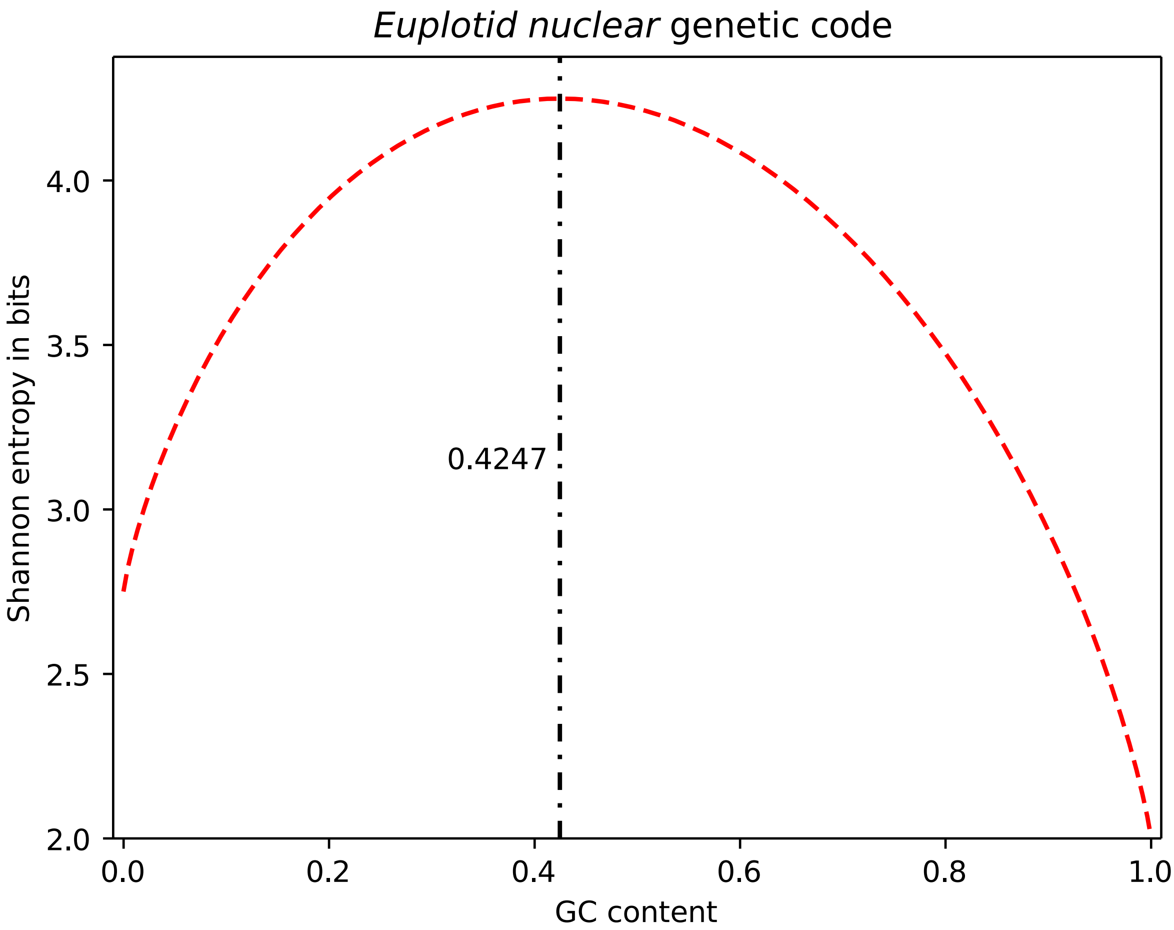

Figure S36. Entropy of the *Euplotid nuclear* genetic code for its given codon assignments and GC contents between 0 % and 100 % as calculated by Shannon's entropy equation. The dash-dotted line indicates the GC content (42.27 %) at which this code reaches its entropy maximum (4.25 bits).

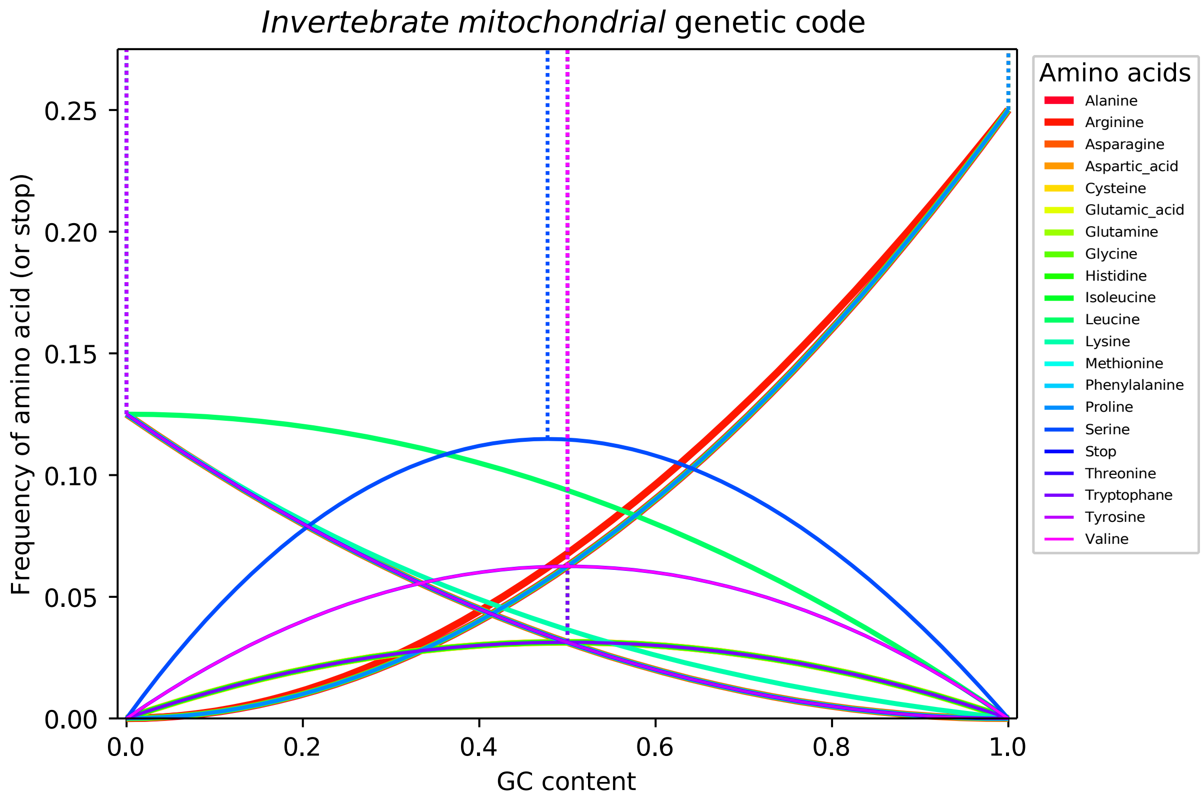

Figure S37. Frequencies of all amino acids (including stop) as encoded by the *invertebrate mitochondrial* genetic code in random sequences as a function of GC content between 0 % and 100 %. The dashed lines mark the maximum achieved frequency for each amino acid (including stop).

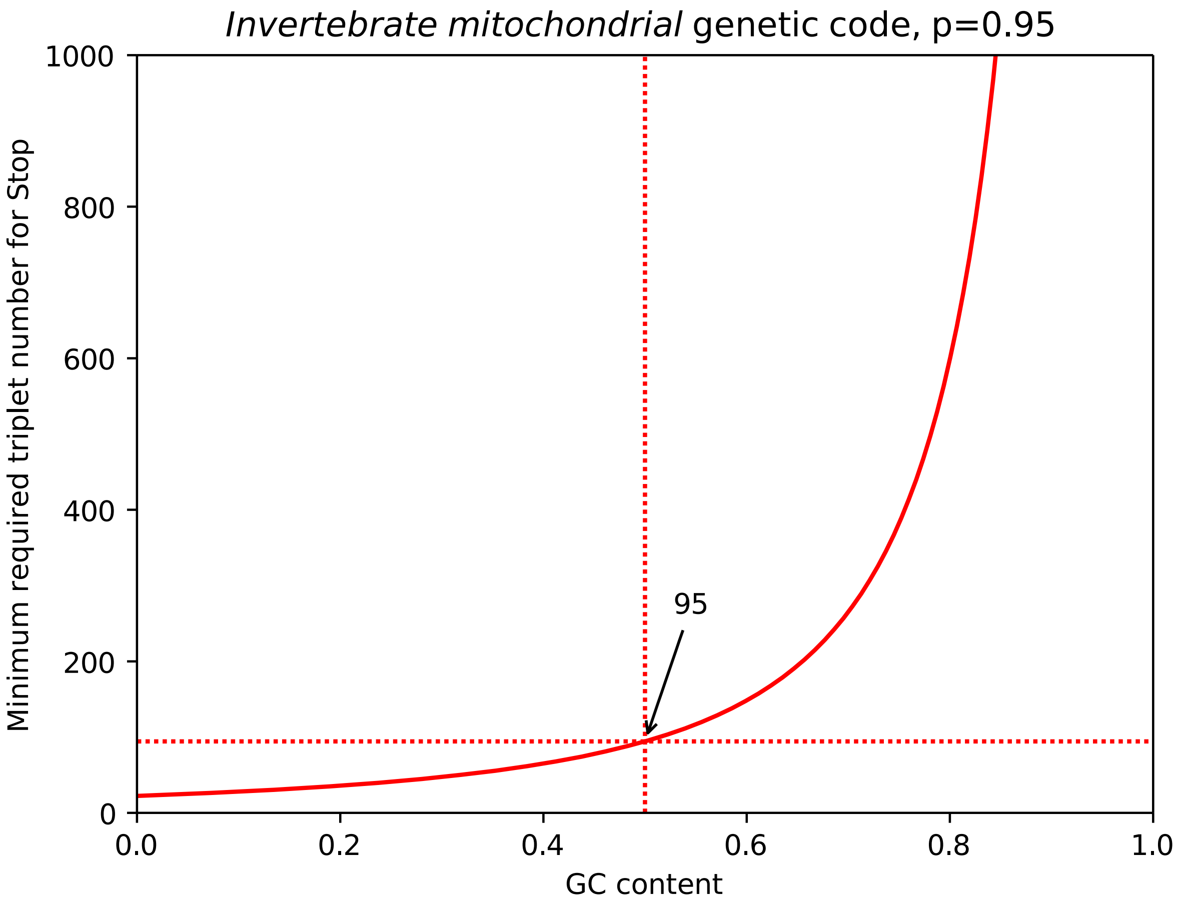

Figure S38. Number of triplets in a random sequence so as to contain at least one stop codon with a probability of 95 % using the *invertebrate mitochondrial* genetic code as a function of GC content. The horizontal and vertical dashed lines indicate the number of triplets for a GC content of 50 % (95 triplets).

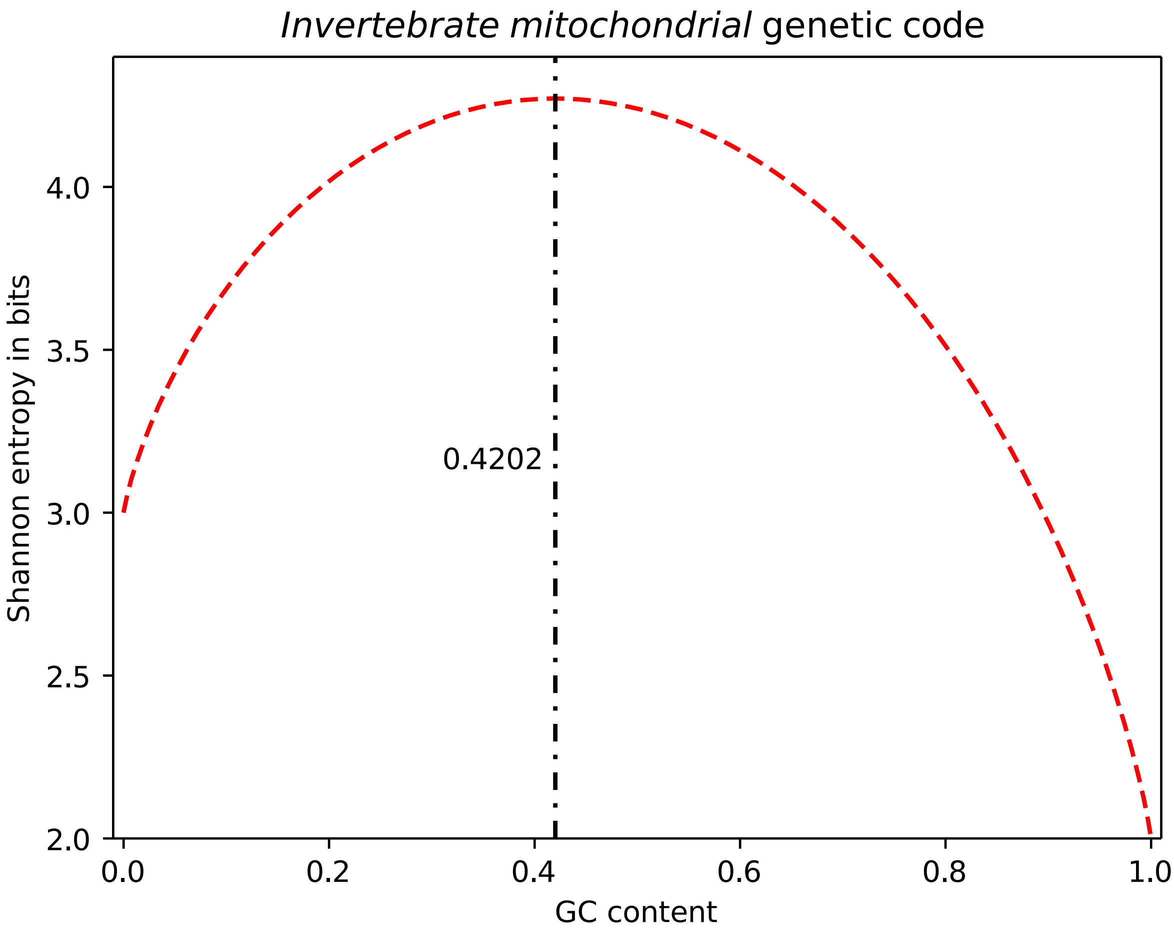

Figure S39. Entropy of the *invertebrate mitochondrial* genetic code for its given codon assignments and GC contents between 0 % and 100 % as calculated by Shannon's entropy equation. The dash-dotted line indicates the GC content (42.02 %) at which this code reaches its entropy maximum (4.27 bits).

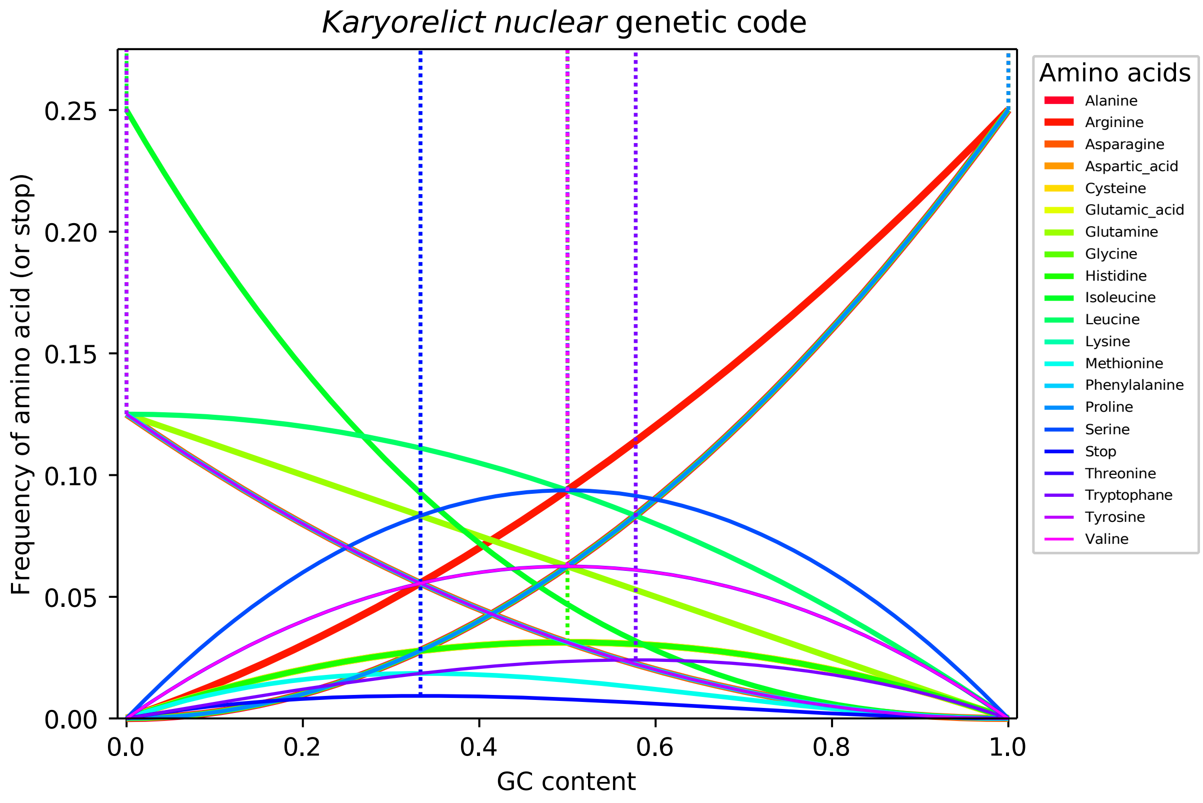

Figure S40. Frequencies of all amino acids (including stop) as encoded by the *Karyorelict nuclear* genetic code in random sequences as a function of GC content between 0 % and 100 %. The dashed lines mark the maximum achieved frequency for each amino acid (including stop).

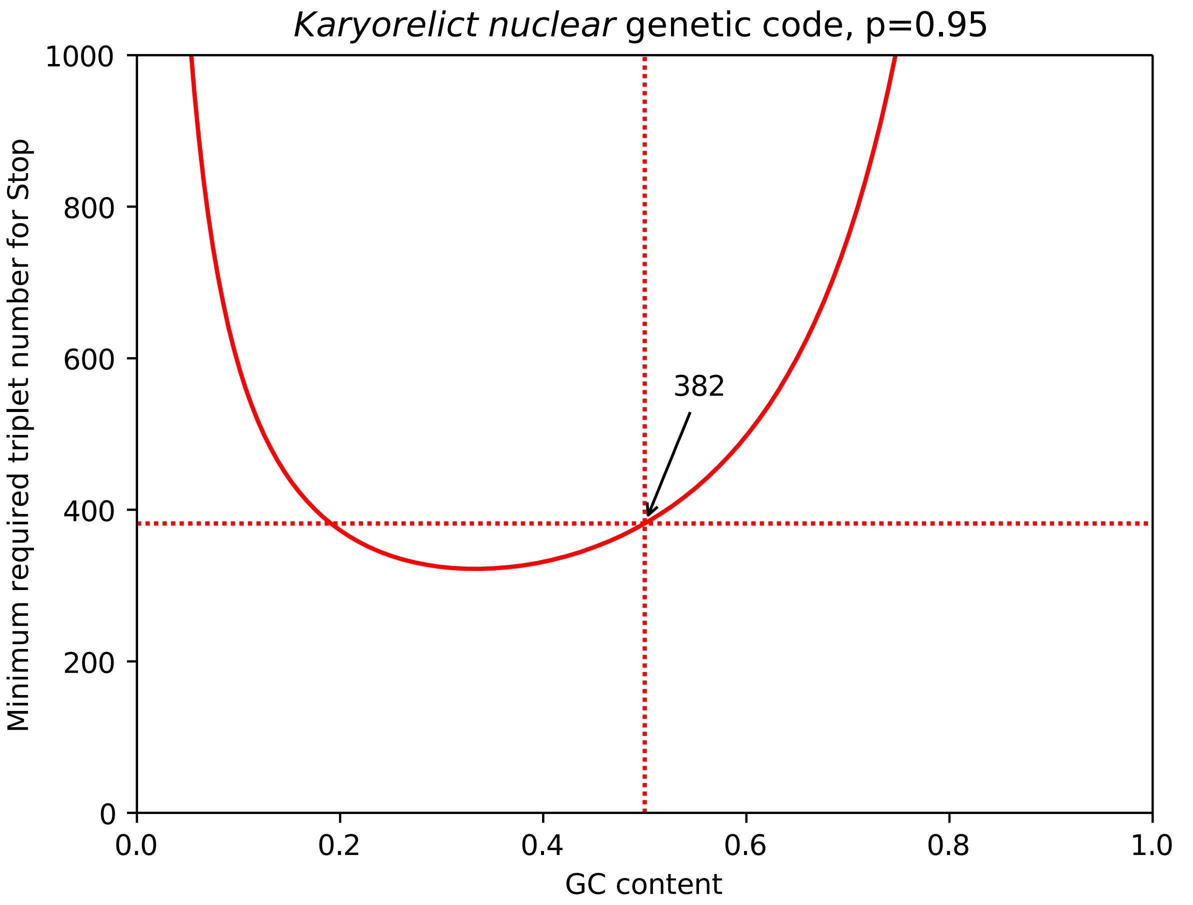

Figure S41. Number of triplets in a random sequence so as to contain at least one stop codon with a probability of 95 % using the *Karyorelict nuclear* genetic code as a function of GC content. The horizontal and vertical dashed lines indicate the number of triplets for a GC content of 50 % (382 triplets).

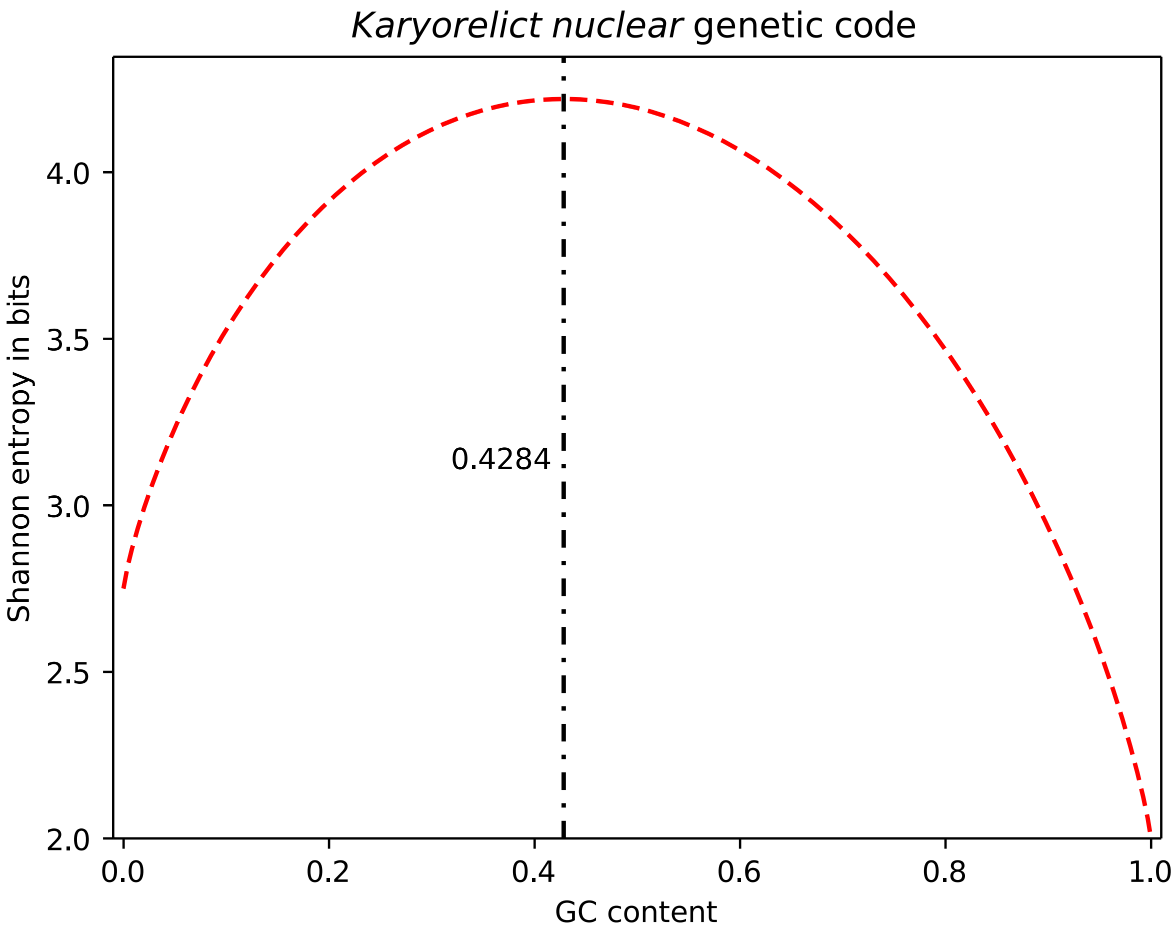

Figure S42. Entropy of the *Karyorelict nuclear* genetic code for its given codon assignments and GC contents between 0 % and 100 % as calculated by Shannon's entropy equation. The dash-dotted line indicates the GC content (42.84 %) at which this code reaches its entropy maximum (4.22 bits).

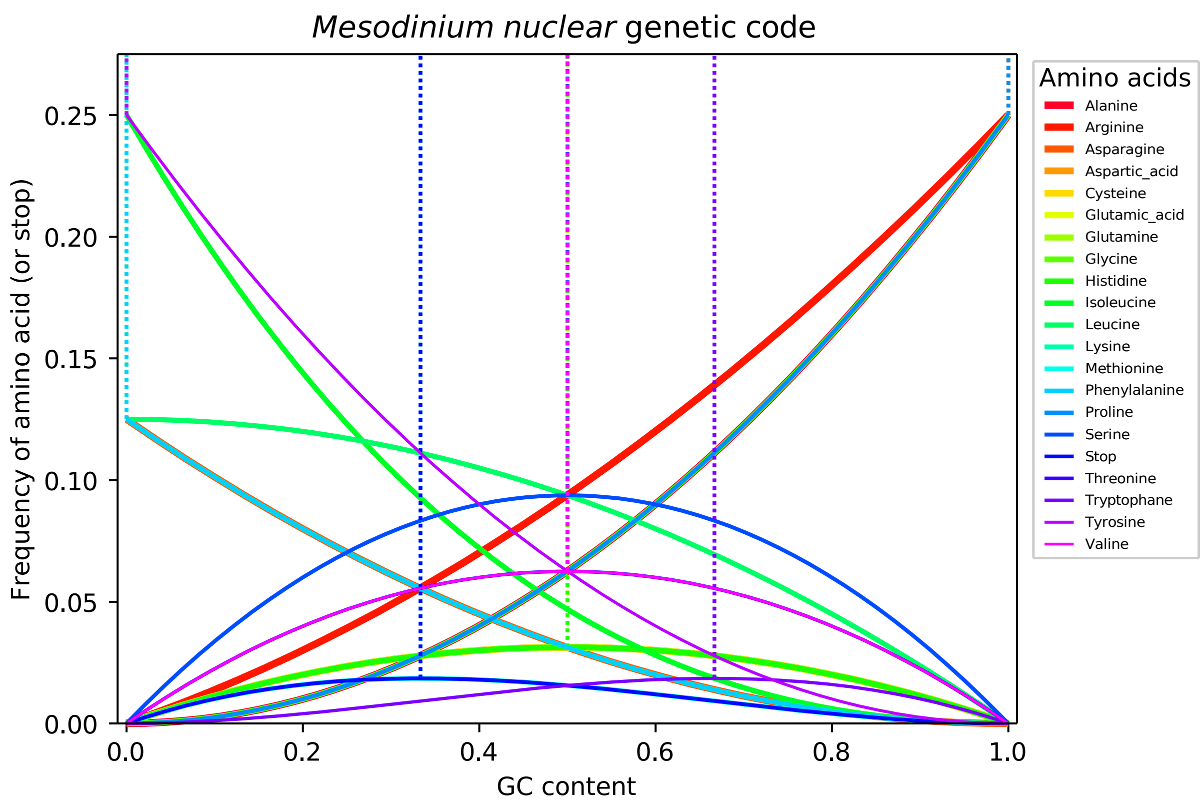

Figure S43. Frequencies of all amino acids (including stop) as encoded by the *Mesodinium nuclear* genetic code in random sequences as a function of GC content between 0 % and 100 %. The dashed lines mark the maximum achieved frequency for each amino acid (including stop).

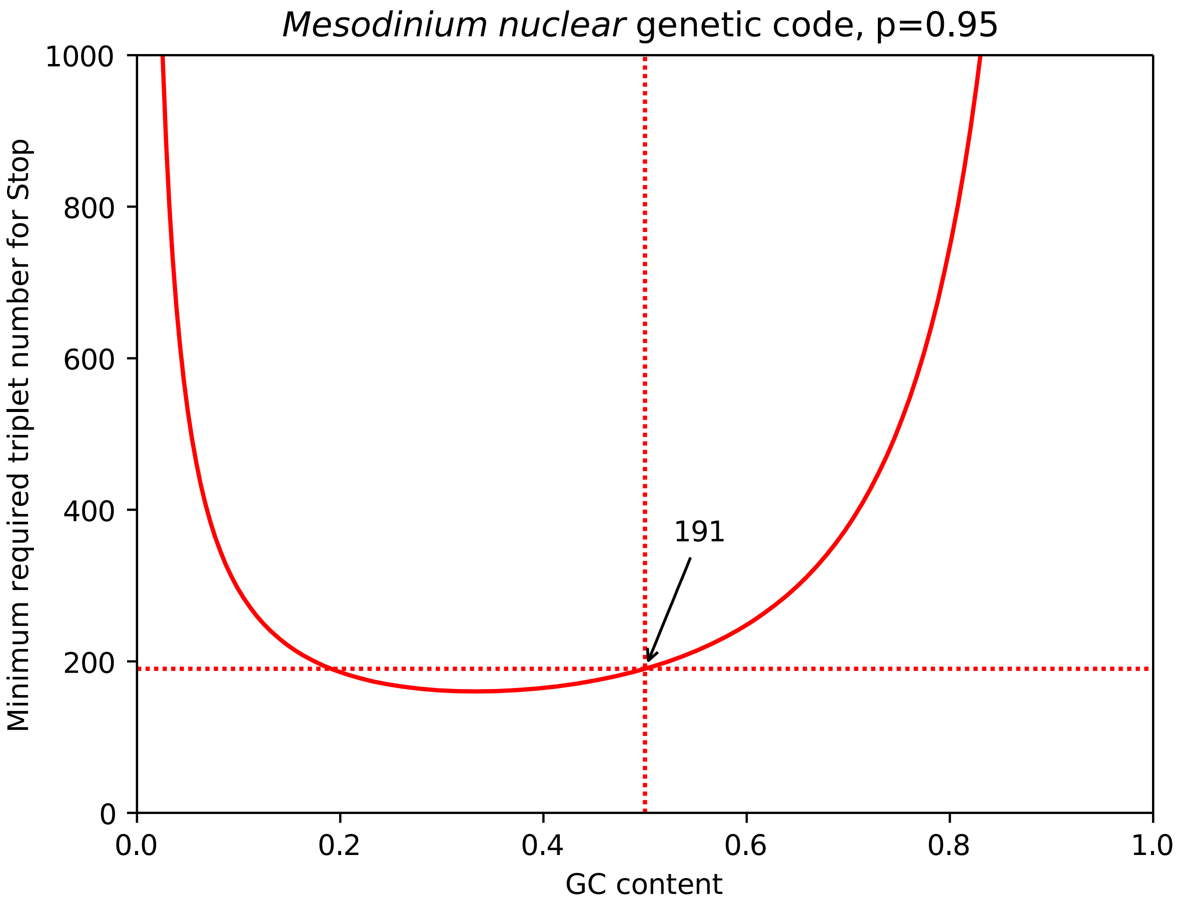

Figure S44. Number of triplets in a random sequence so as to contain at least one stop codon with a probability of 95 % using the *Mesodinium nuclear* genetic code as a function of GC content. The horizontal and vertical dashed lines indicate the number of triplets for a GC content of 50 % (191 triplets).

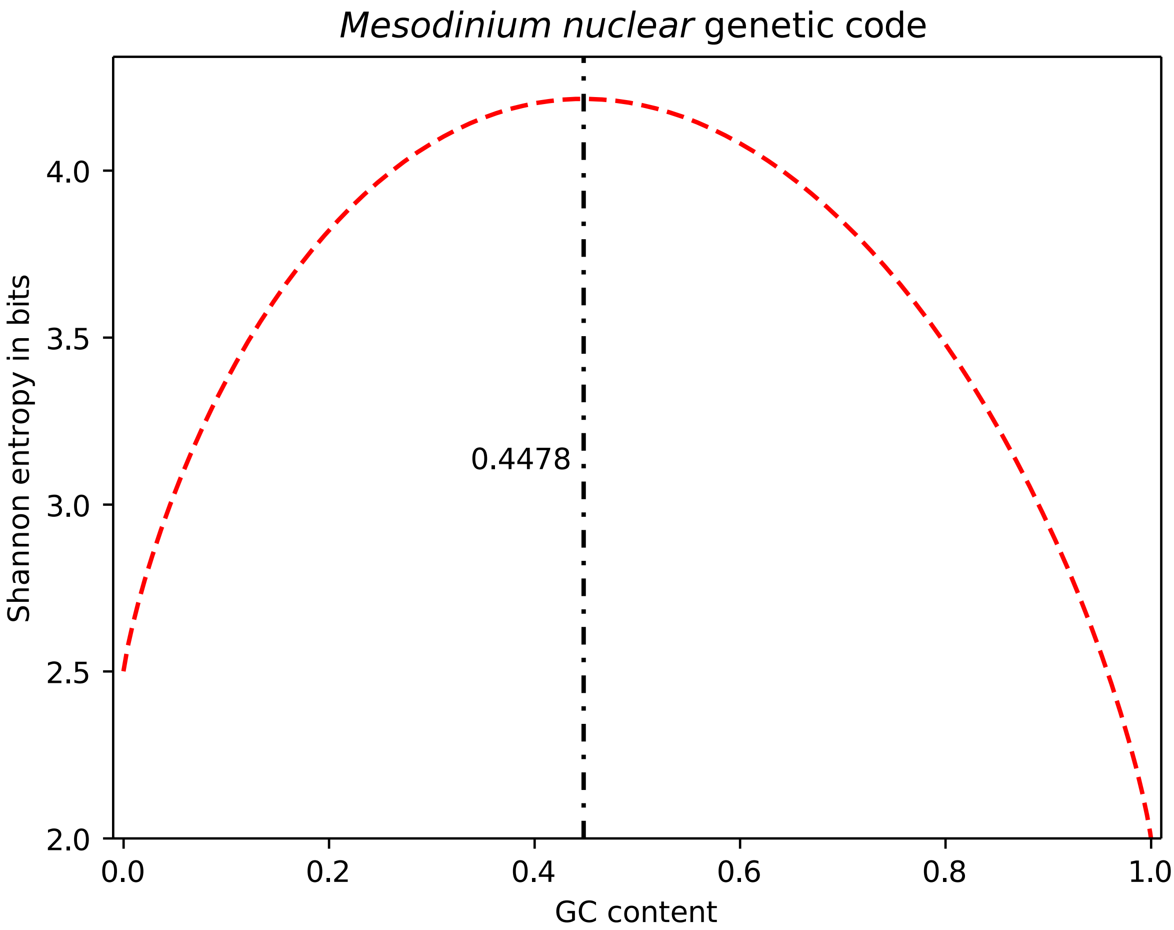

Figure S45. Entropy of the *Mesodinium nuclear* genetic code for its given codon assignments and GC contents between 0 % and 100 % as calculated by Shannon's entropy equation. The dash-dotted line indicates the GC content (44.78 %) at which this code reaches its entropy maximum (4.21 bits).

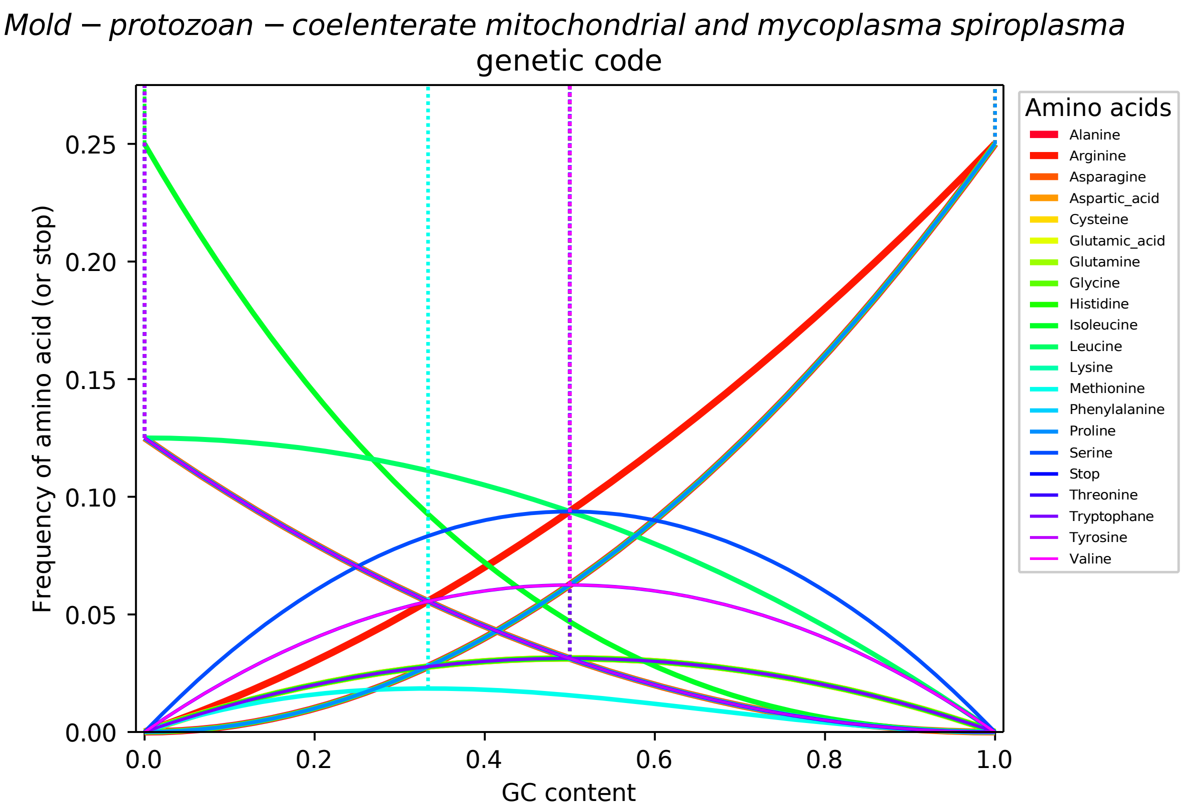

Figure S46. Frequencies of all amino acids (including stop) as encoded by the *Mold – protozoan – coelenterate mitochondrial and mycoplasma Spiroplasma* genetic code in random sequences as a function of GC content between 0 % and 100 %. The dashed lines mark the maximum achieved frequency for each amino acid (including stop).

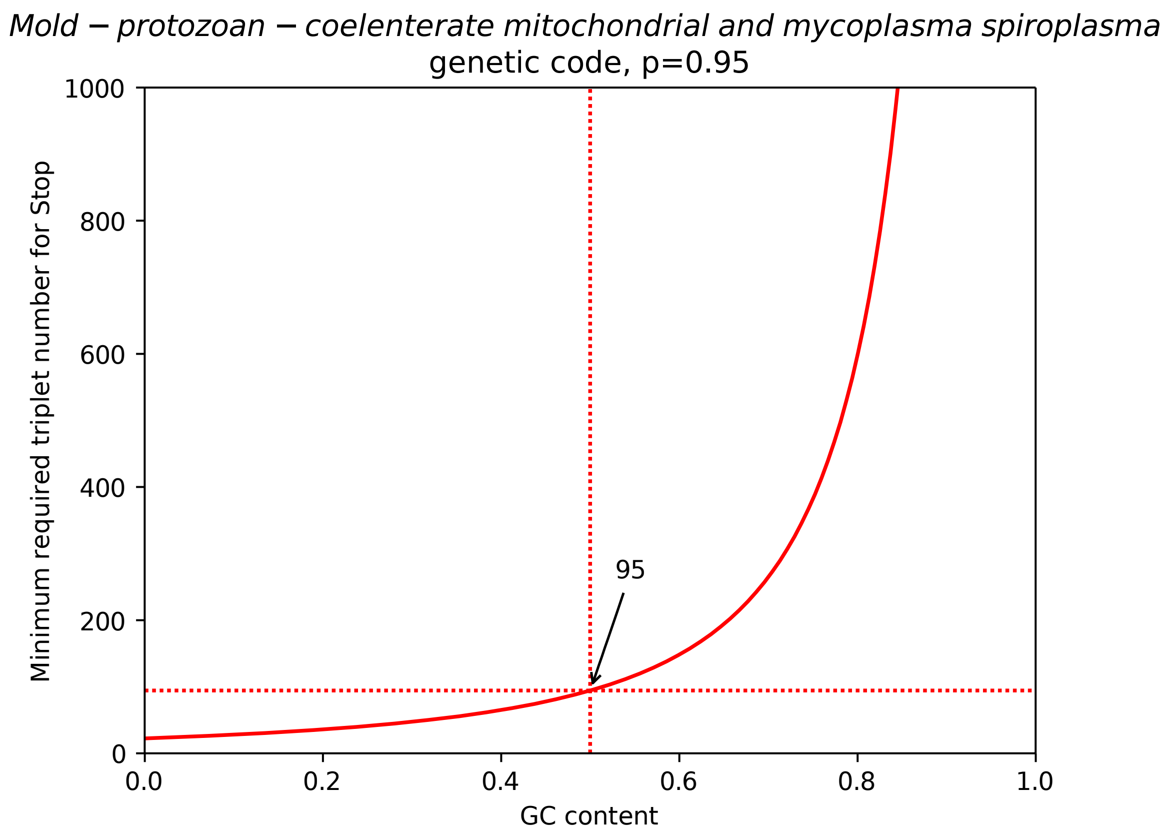

Figure S47. Number of triplets in a random sequence so as to contain at least one stop codon with a probability of 95 % using the *Mold – protozoan – coelenterate mitochondrial and mycoplasma Spiroplasma* genetic code as a function of GC content. The horizontal and vertical dashed lines indicate the number of triplets for a GC content of 50 % (95 triplets).

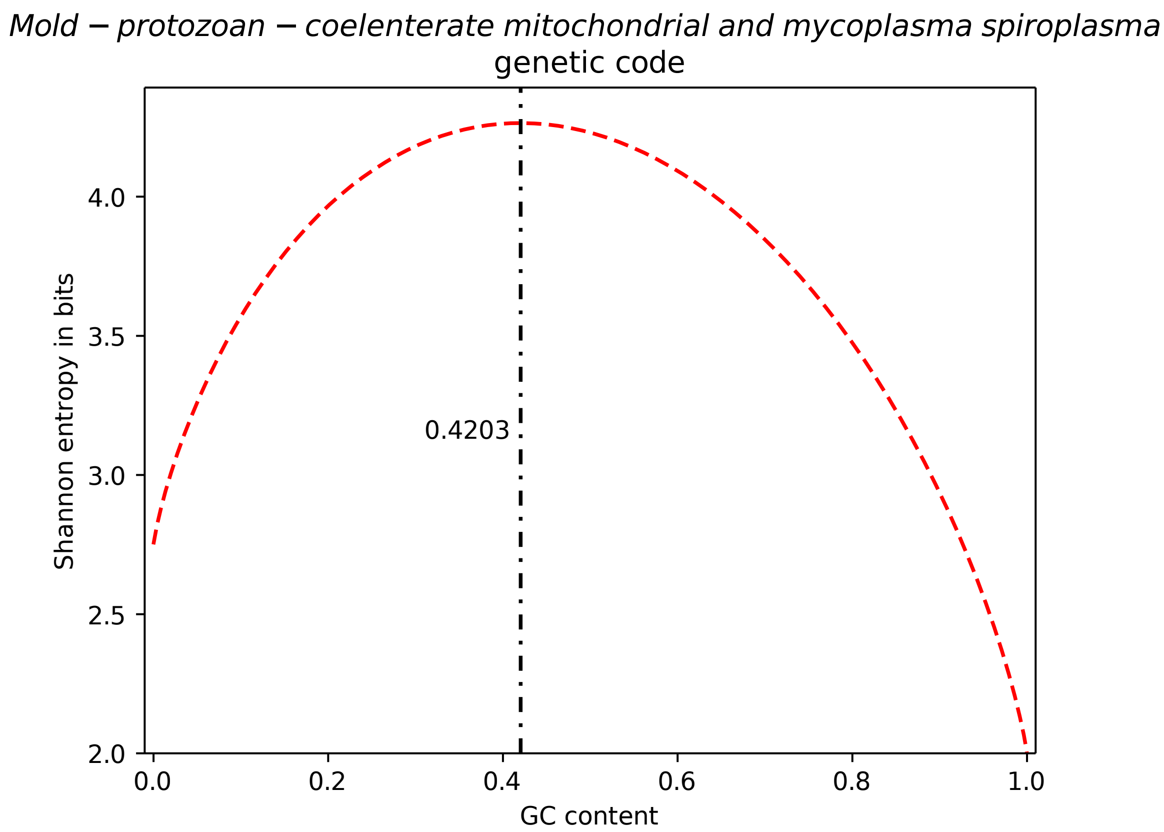

Figure S48. Entropy of the *Mold – protozoan – coelenterate mitochondrial and mycoplasma Spiroplasma* genetic code for its given codon assignments and GC contents between 0 % and 100 % as calculated by Shannon's entropy equation. The dash-dotted line indicates the GC content (42.03 %) at which this code reaches its entropy maximum (4.26 bits).

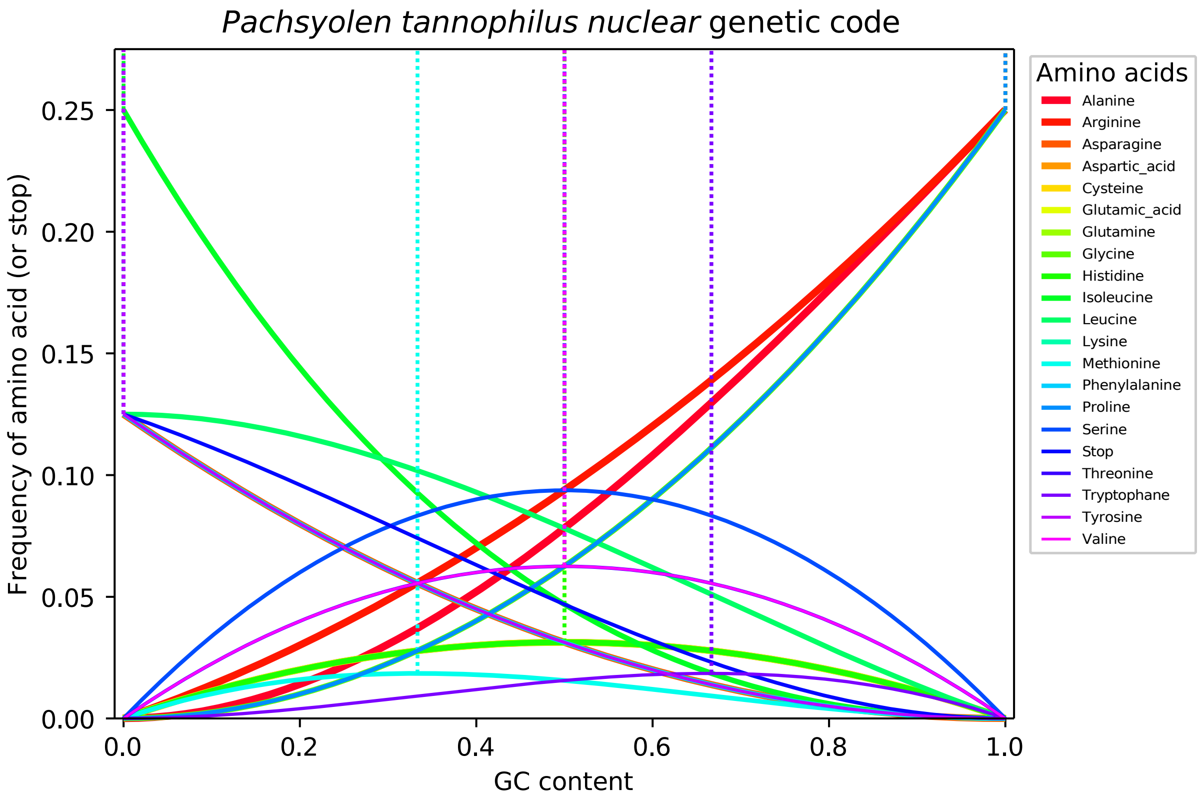

Figure S49. Frequencies of all amino acids (including stop) as encoded by the *Pachsyolen Tannophilus nuclear* genetic code in random sequences as a function of GC content between 0 % and 100 %. The dashed lines mark the maximum achieved frequency for each amino acid (including stop).

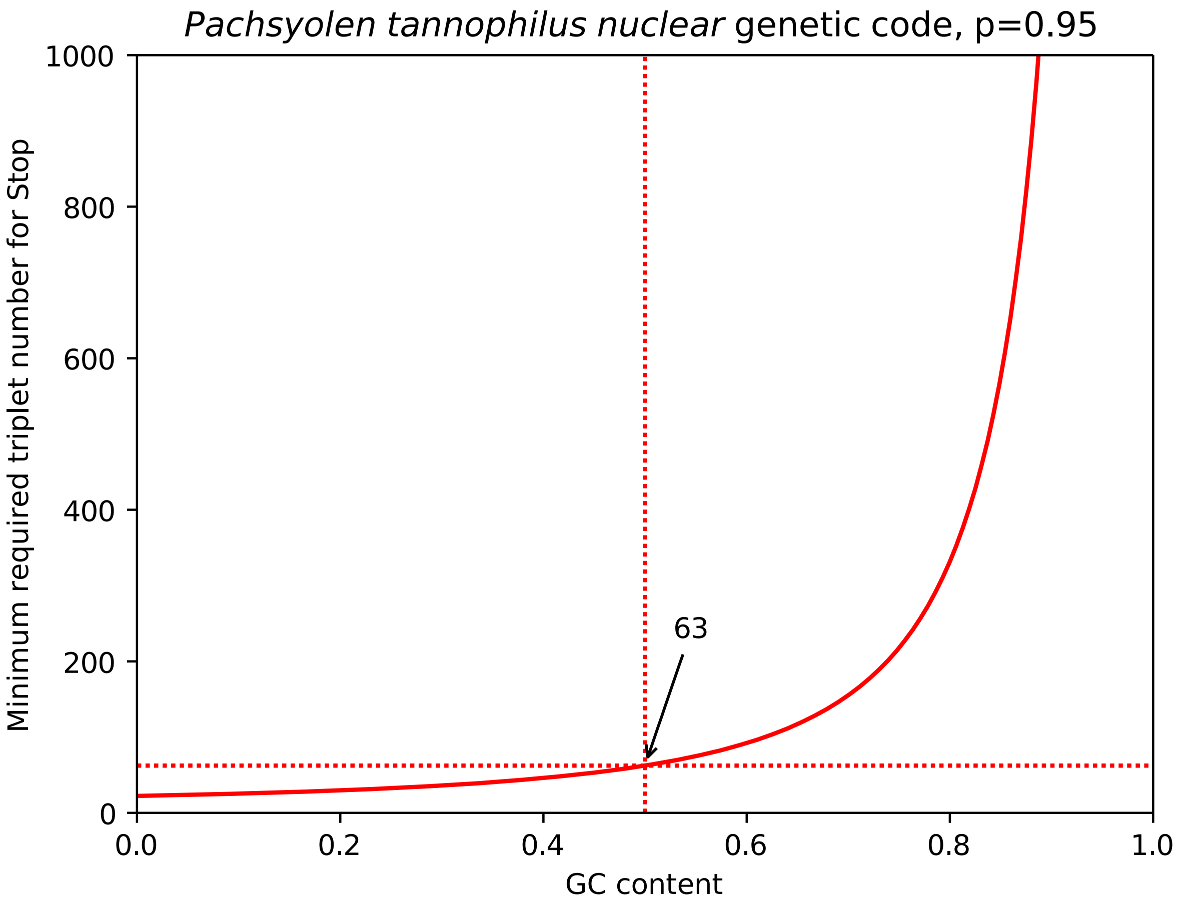

Figure S50. Number of triplets in a random sequence so as to contain at least one stop codon with a probability of 95 % using the *Pachsyolen Tannophilus nuclear* genetic code as a function of GC content. The horizontal and vertical dashed lines indicate the number of triplets for a GC content of 50 % (63 triplets).

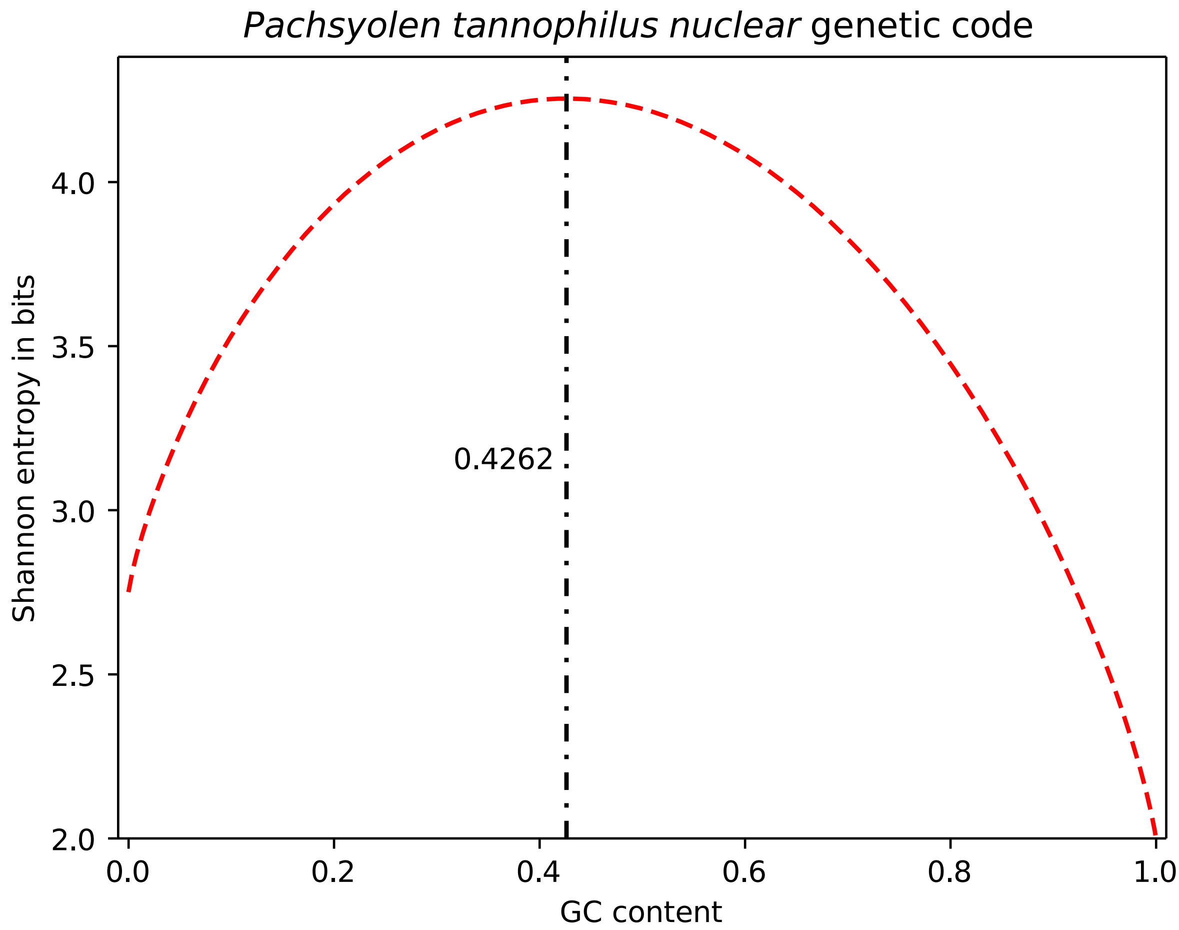

Figure S51. Entropy of the *Pachsyolen Tannophilus* nuclear genetic code for its given codon assignments and GC contents between 0 % and 100 % as calculated by Shannon's entropy equation. The dash-dotted line indicates the GC content (42.21 %) at which this code reaches its entropy maximum (4.25 bits).

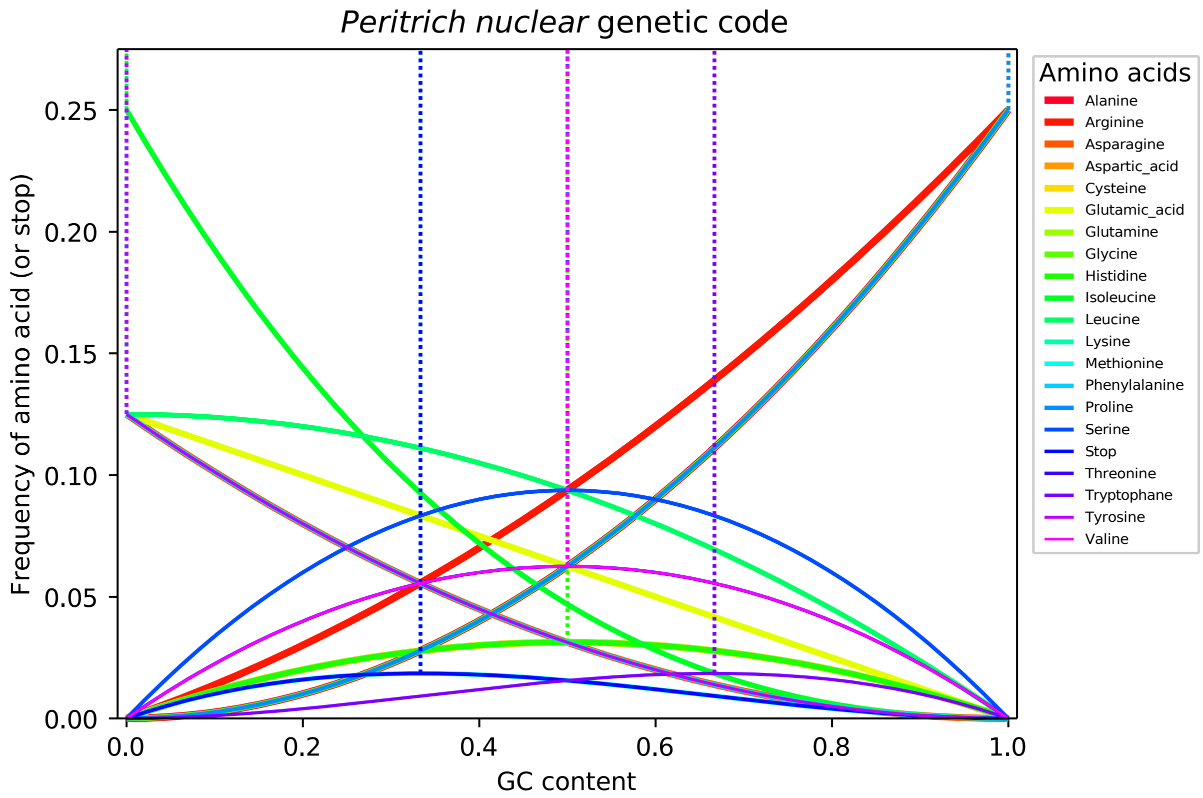

Figure S52. Frequencies of all amino acids (including stop) as encoded by the *Peritrich nuclear* genetic code in random sequences as a function of GC content between 0 % and 100 %. The dashed lines mark the maximum achieved frequency for each amino acid (including stop).

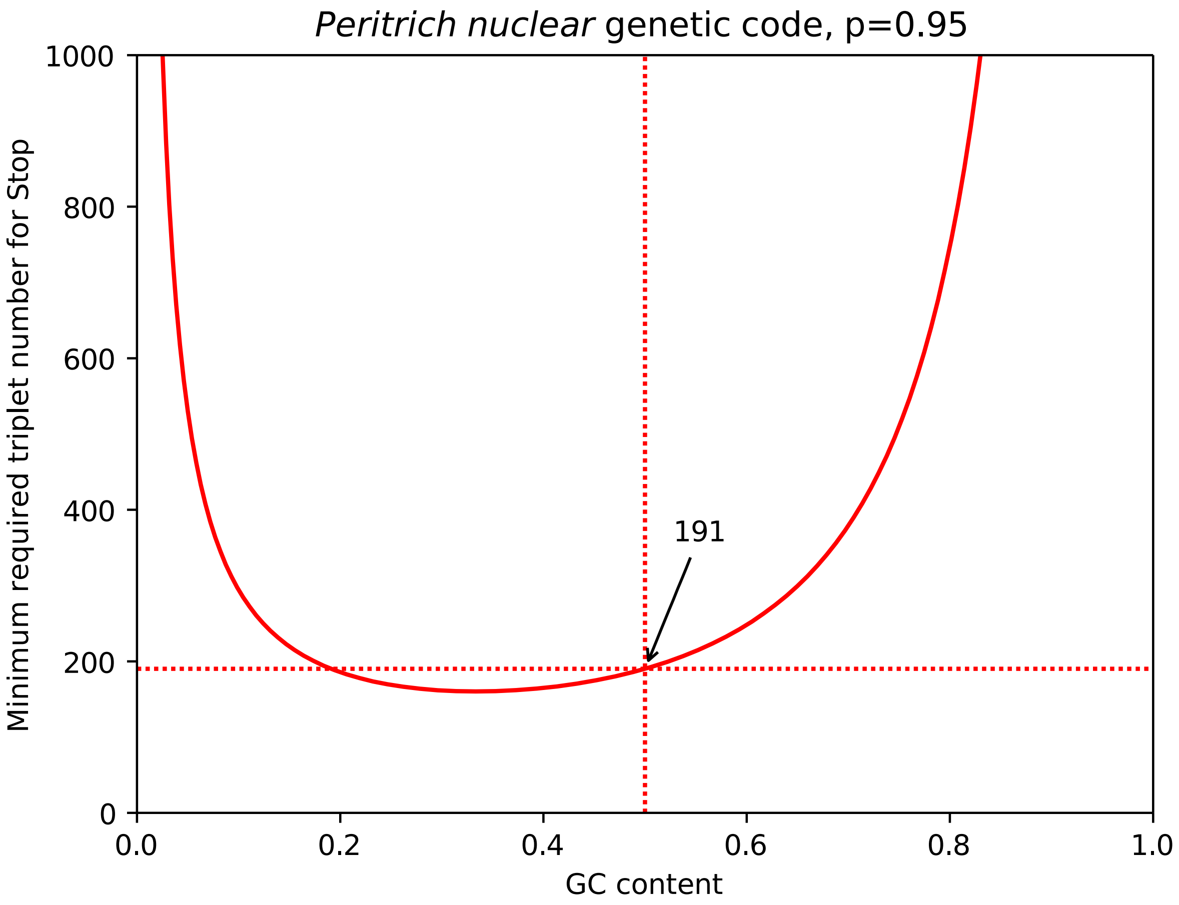

Figure S53. Number of triplets in a random sequence so as to contain at least one stop codon with a probability of 95 % using the *Peritrich nuclear* genetic code as a function of GC content. The horizontal and vertical dashed lines indicate the number of triplets for a GC content of 50 % (191 triplets).

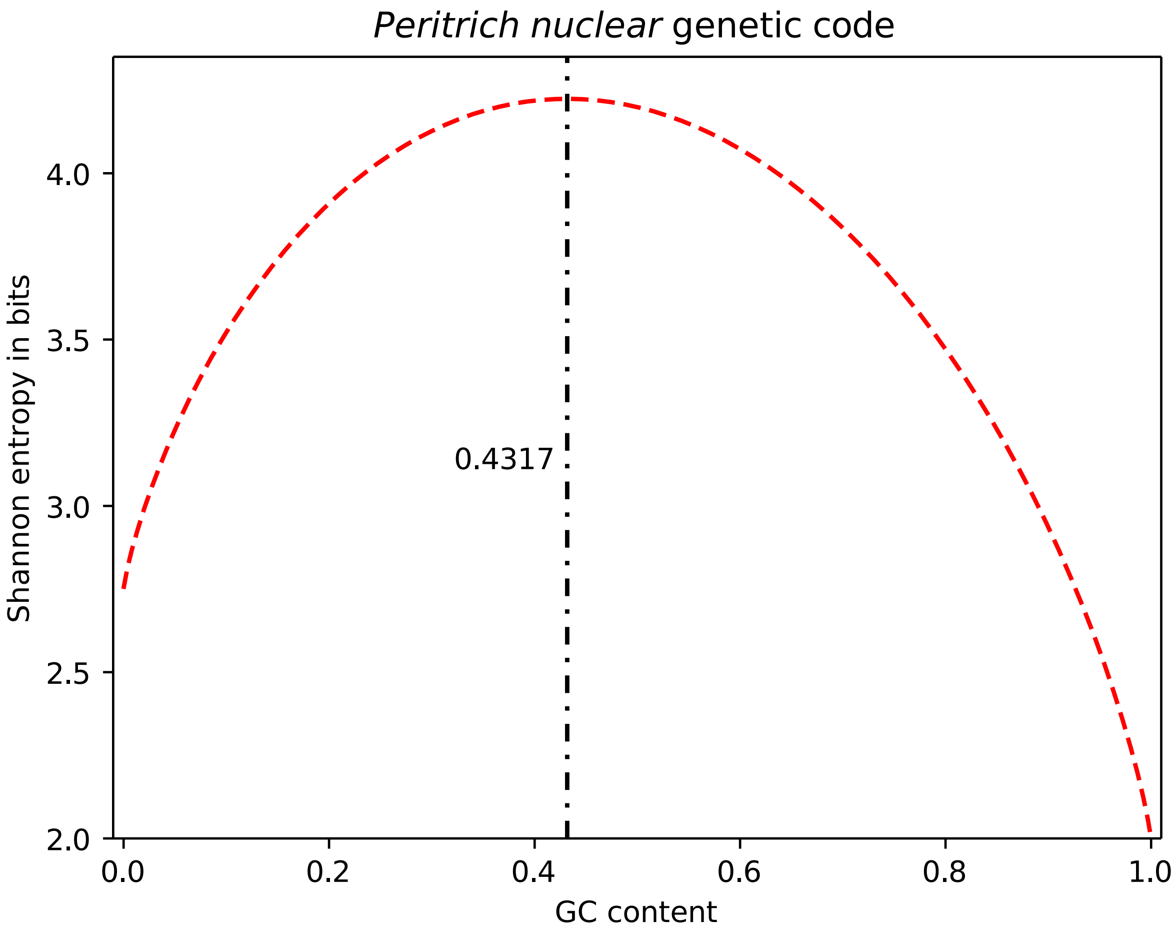

Figure S54. Entropy of the *Peritrich nuclear* genetic code for its given codon assignments and GC contents between 0 % and 100 % as calculated by Shannon's entropy equation. The dash-dotted line indicates the GC content (43.17 %) at which this code reaches its entropy maximum (4.22 bits).

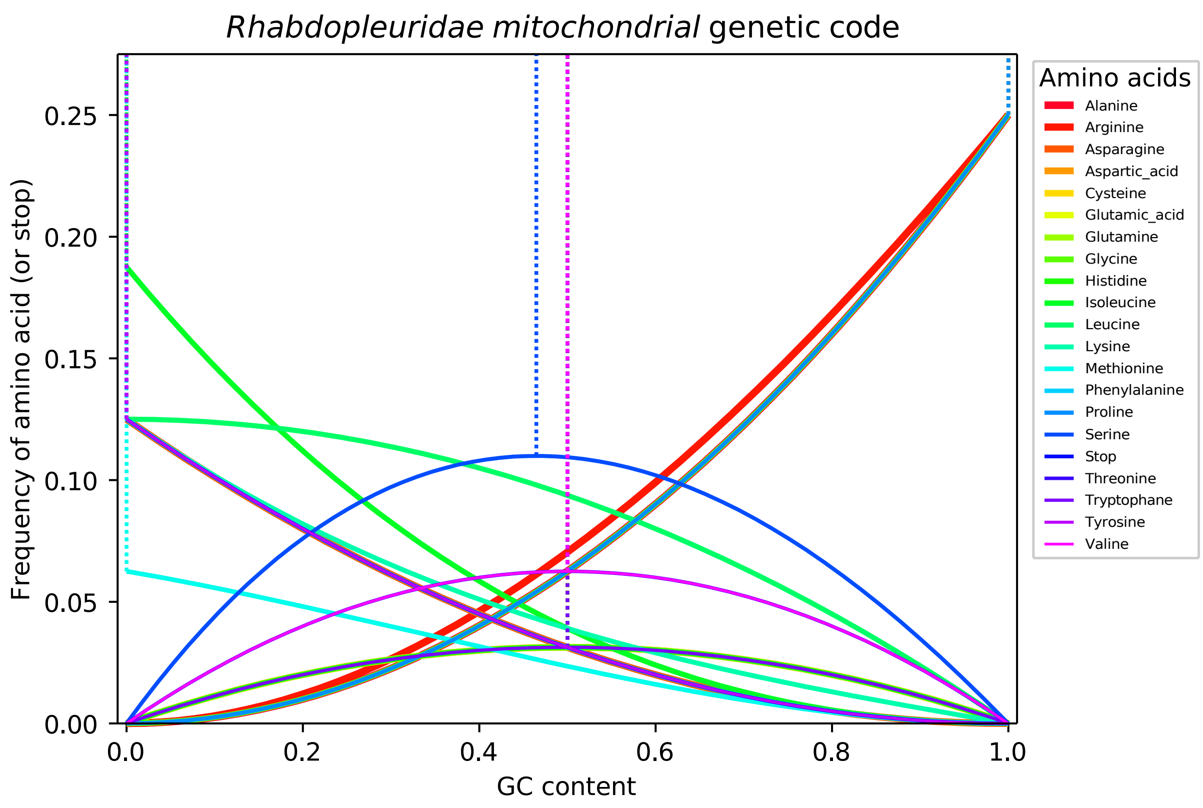

Figure S55. Frequencies of all amino acids (including stop) as encoded by the *Rhabdopleuridae mitochondrial* genetic code in random sequences as a function of GC content between 0 % and 100 %. The dashed lines mark the maximum achieved frequency for each amino acid (including stop).

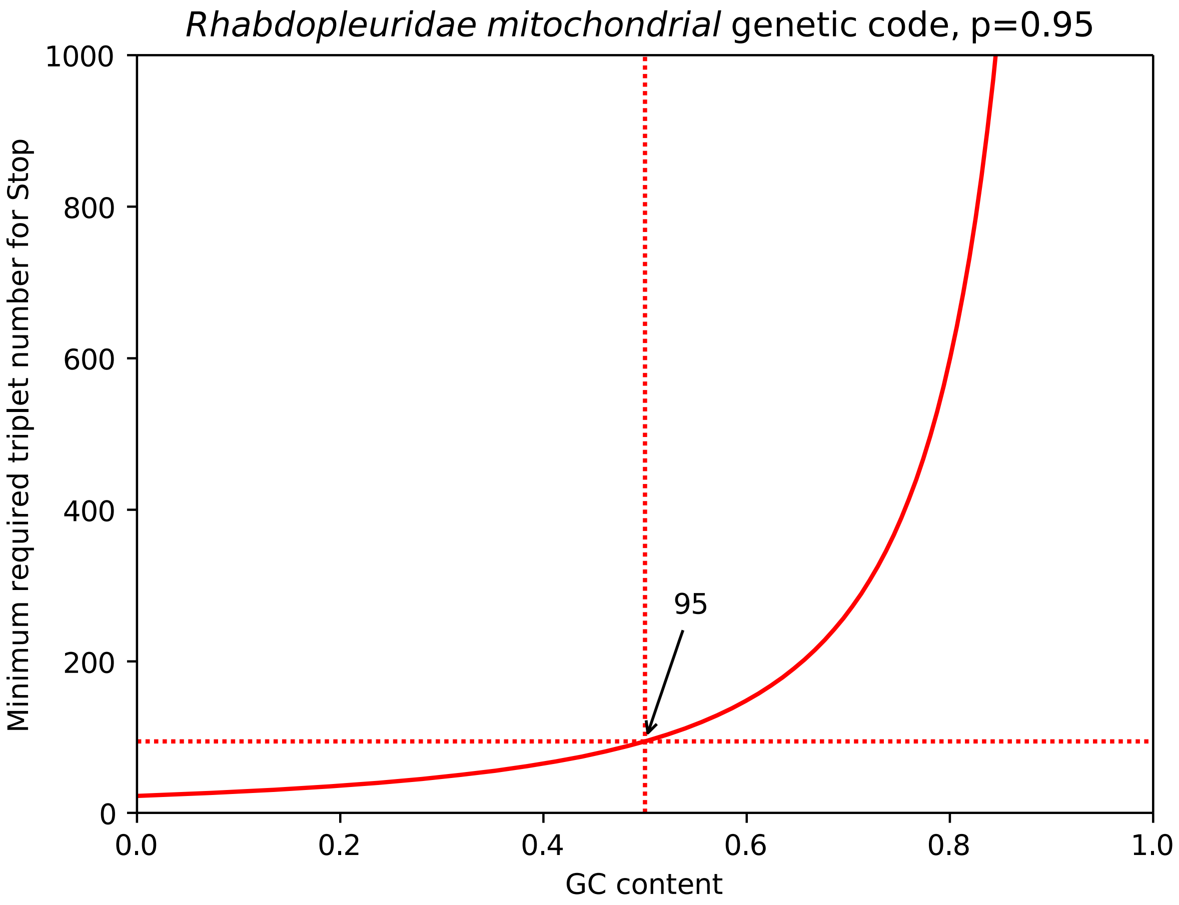

Figure S56. Number of triplets in a random sequence so as to contain at least one stop codon with a probability of 95 % using the *Rhabdopleuridae mitochondrial* genetic code as a function of GC content. The horizontal and vertical dashed lines indicate the number of triplets for a GC content of 50 % (95 triplets).

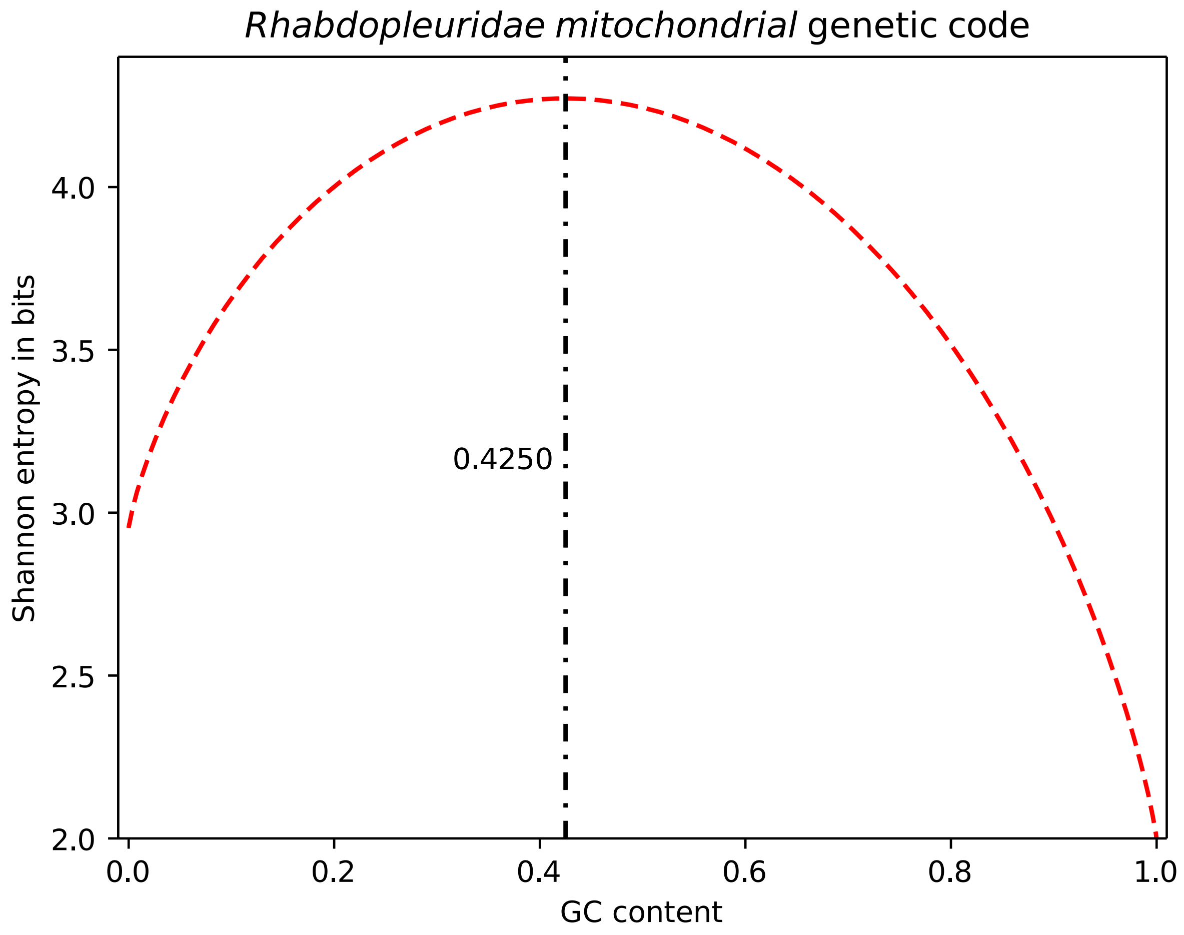

Figure S57. Entropy of the *Rhabdopleuridae mitochondrial* genetic code for its given codon assignments and GC contents between 0 % and 100 % as calculated by Shannon's entropy equation. The dash-dotted line indicates the GC content (42.50 %) at which this code reaches its entropy maximum (4.27 bits).

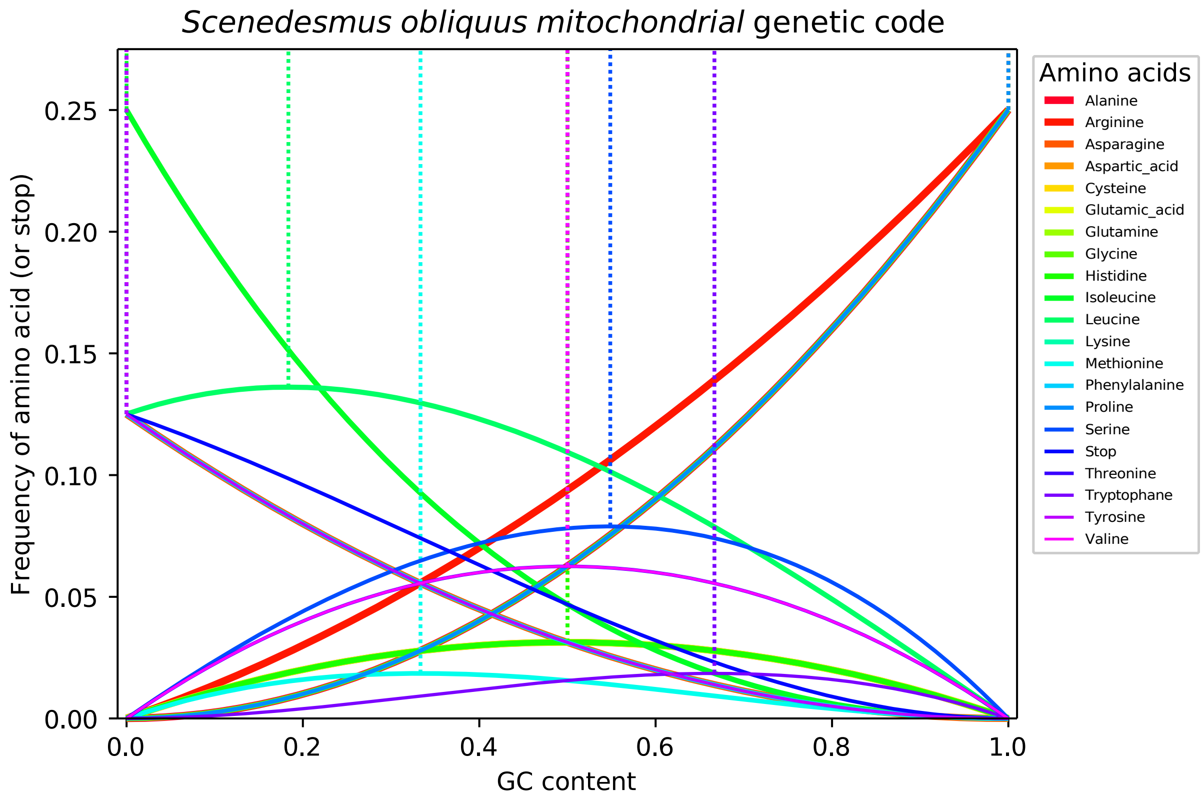

Figure S58. Frequencies of all amino acids (including stop) as encoded by the *Scenedesmus obliquus mitochondrial* genetic code in random sequences as a function of GC content between 0 % and 100 %. The dashed lines mark the maximum achieved frequency for each amino acid (including stop).

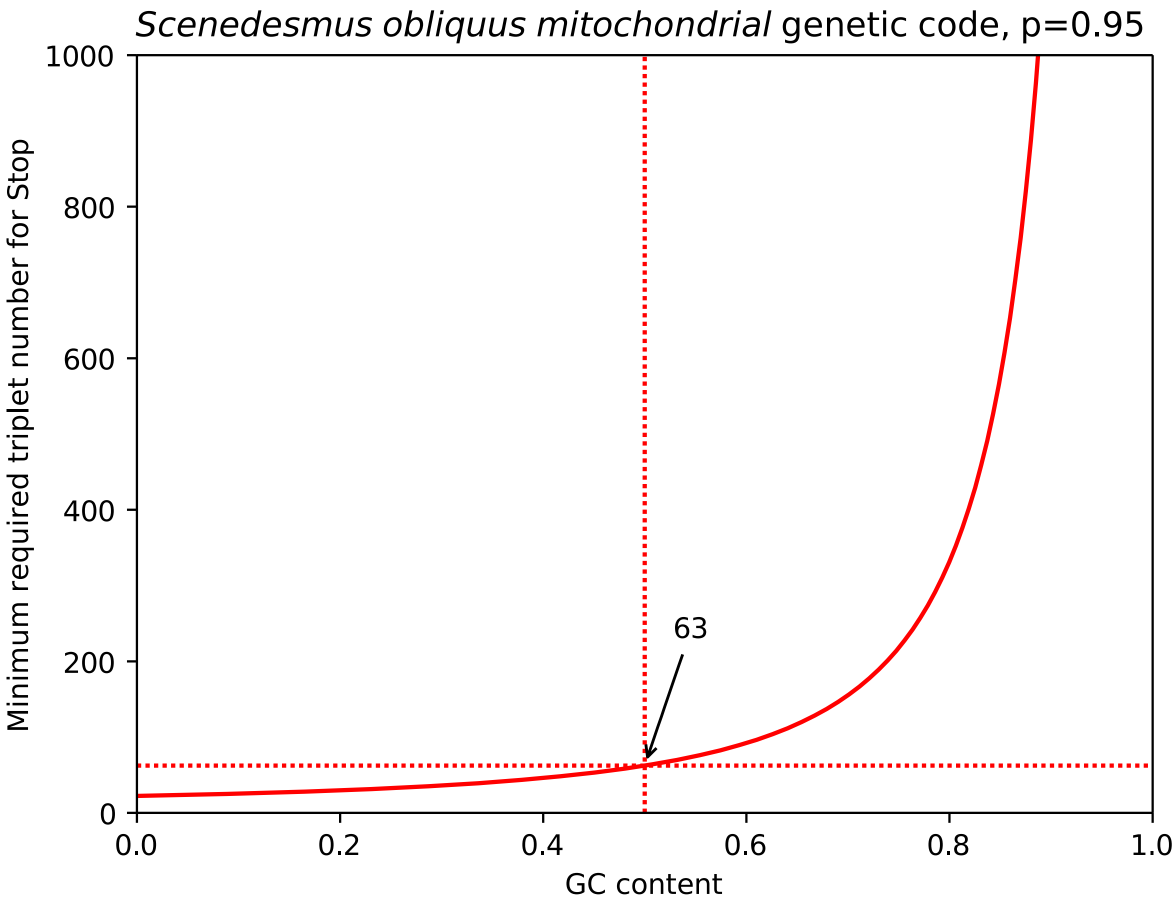

Figure S59. Number of triplets in a random sequence so as to contain at least one stop codon with a probability of 95 % using the *Scenedesmus obliquus mitochondrial* genetic code as a function of GC content. The horizontal and vertical dashed lines indicate the number of triplets for a GC content of 50 % (63 triplets).

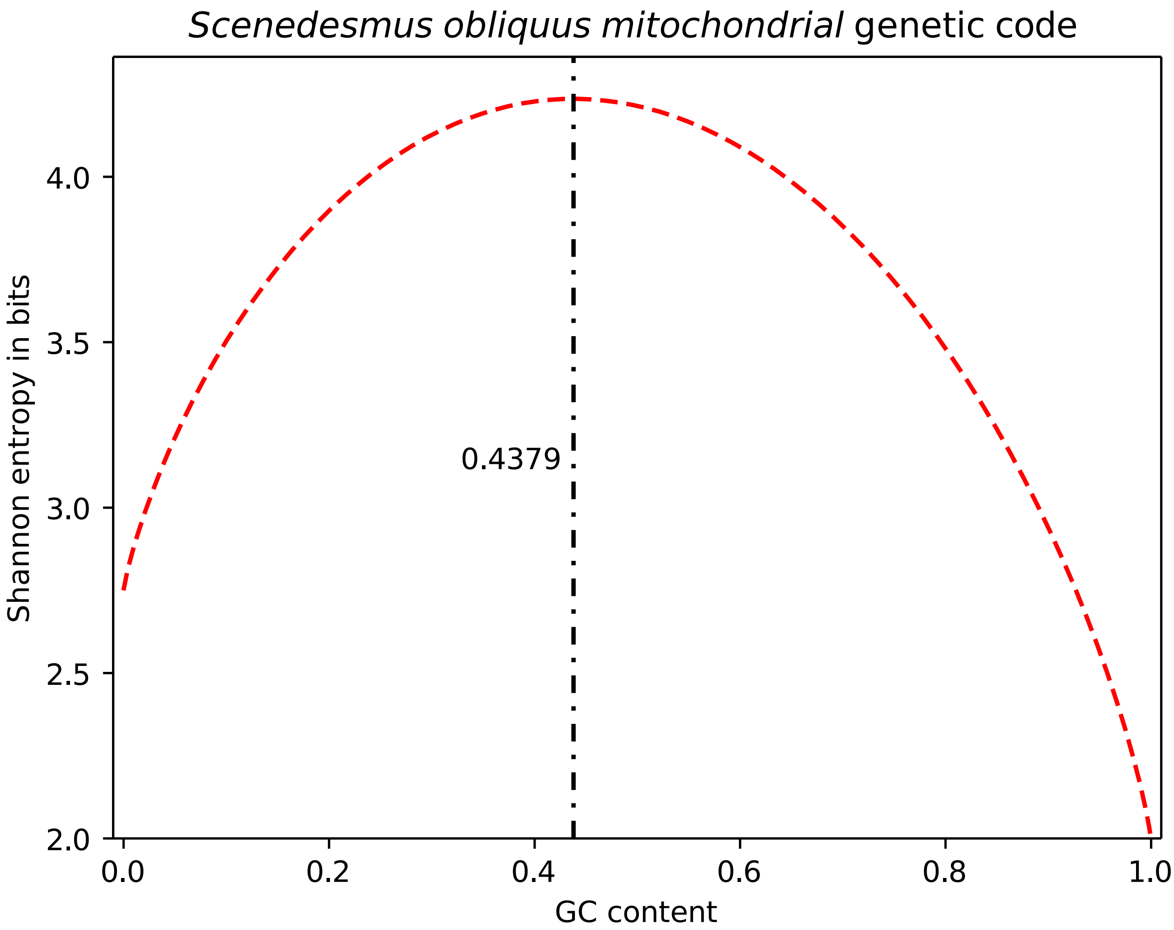

Figure S60. Entropy of the *Scenedesmus obliquus mitochondrial* genetic code for its given codon assignments and GC contents between 0 % and 100 % as calculated by Shannon's entropy equation. The dash-dotted line indicates the GC content (43.79 %) at which this code reaches its entropy maximum (4.24 bits).

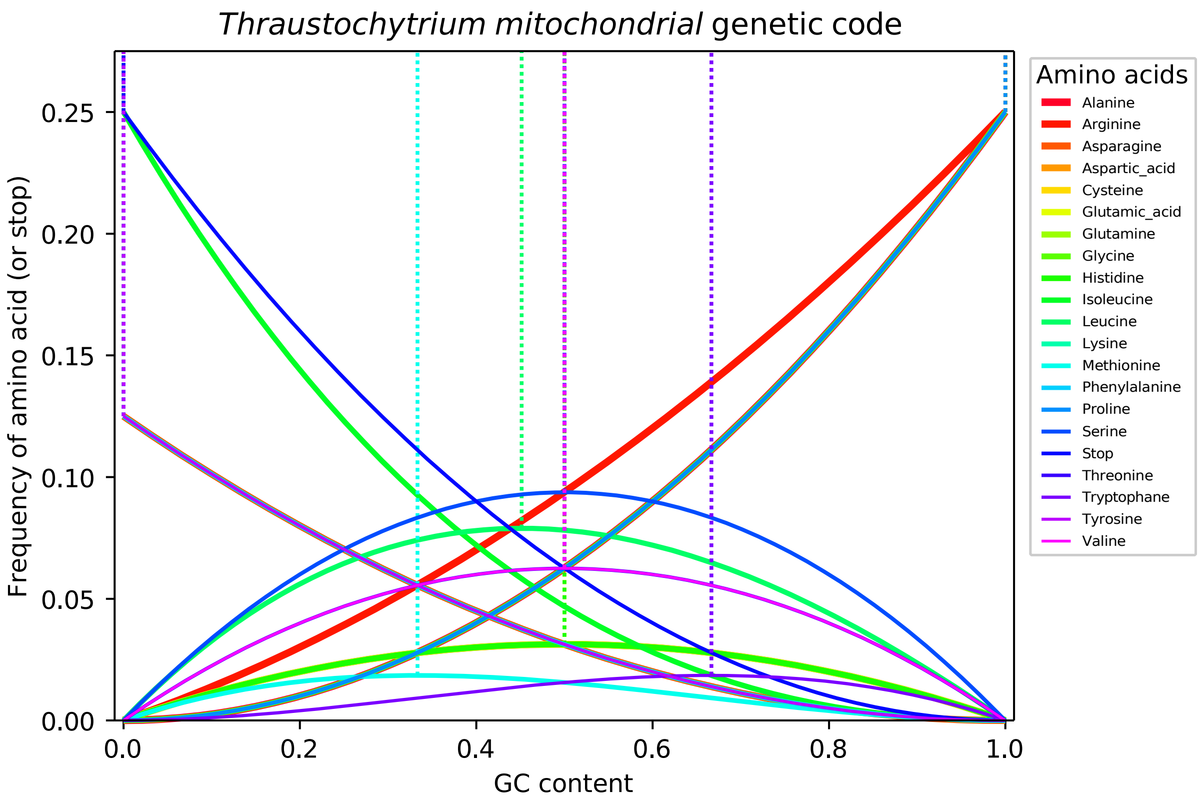

Figure S61. Frequencies of all amino acids (including stop) as encoded by the *Thraustochytrium mitochondrial* genetic code in random sequences as a function of GC content between 0 % and 100 %. The dashed lines mark the maximum achieved frequency for each amino acid (including stop).

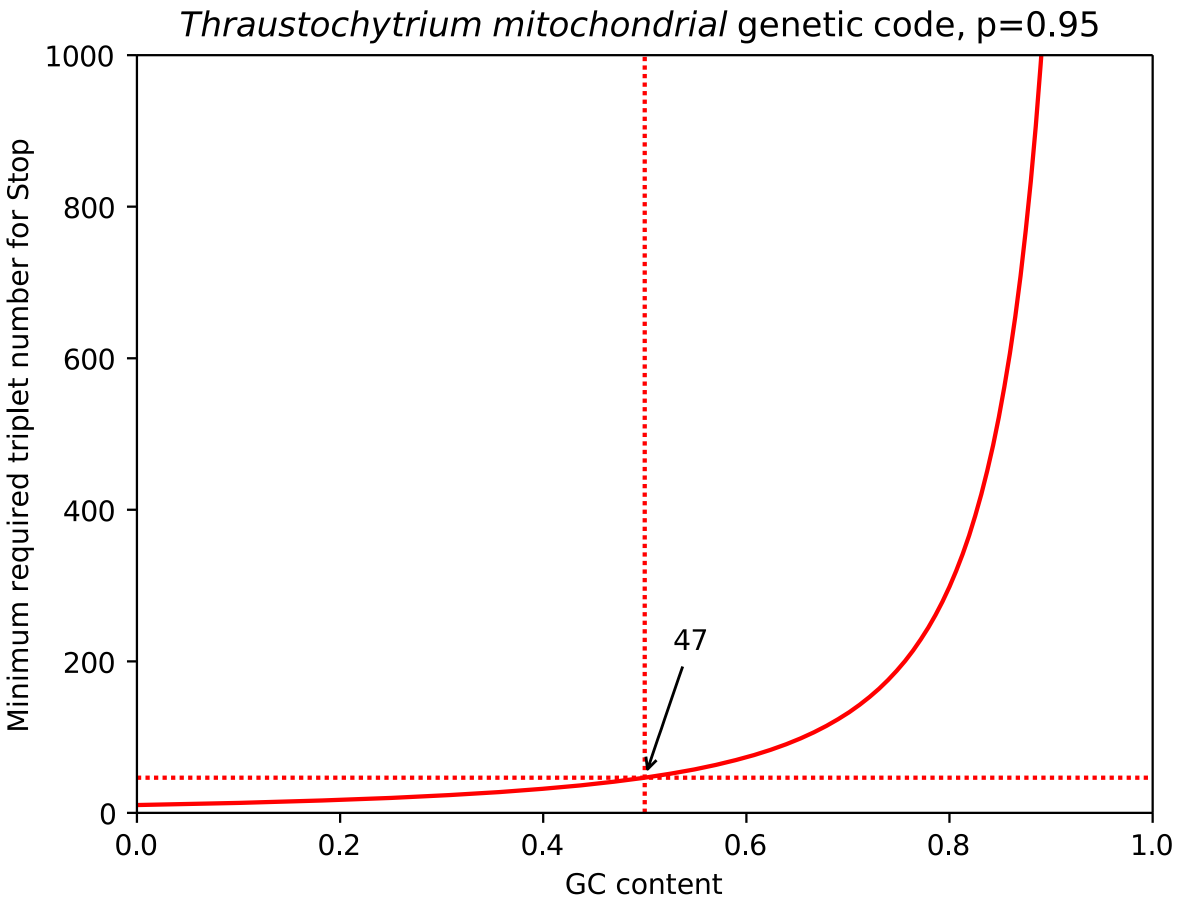

Figure S62. Number of triplets in a random sequence so as to contain at least one stop codon with a probability of 95 % using the *Thraustochytrium mitochondrial* genetic code as a function of GC content. The horizontal and vertical dashed lines indicate the number of triplets for a GC content of 50 % (47 triplets).

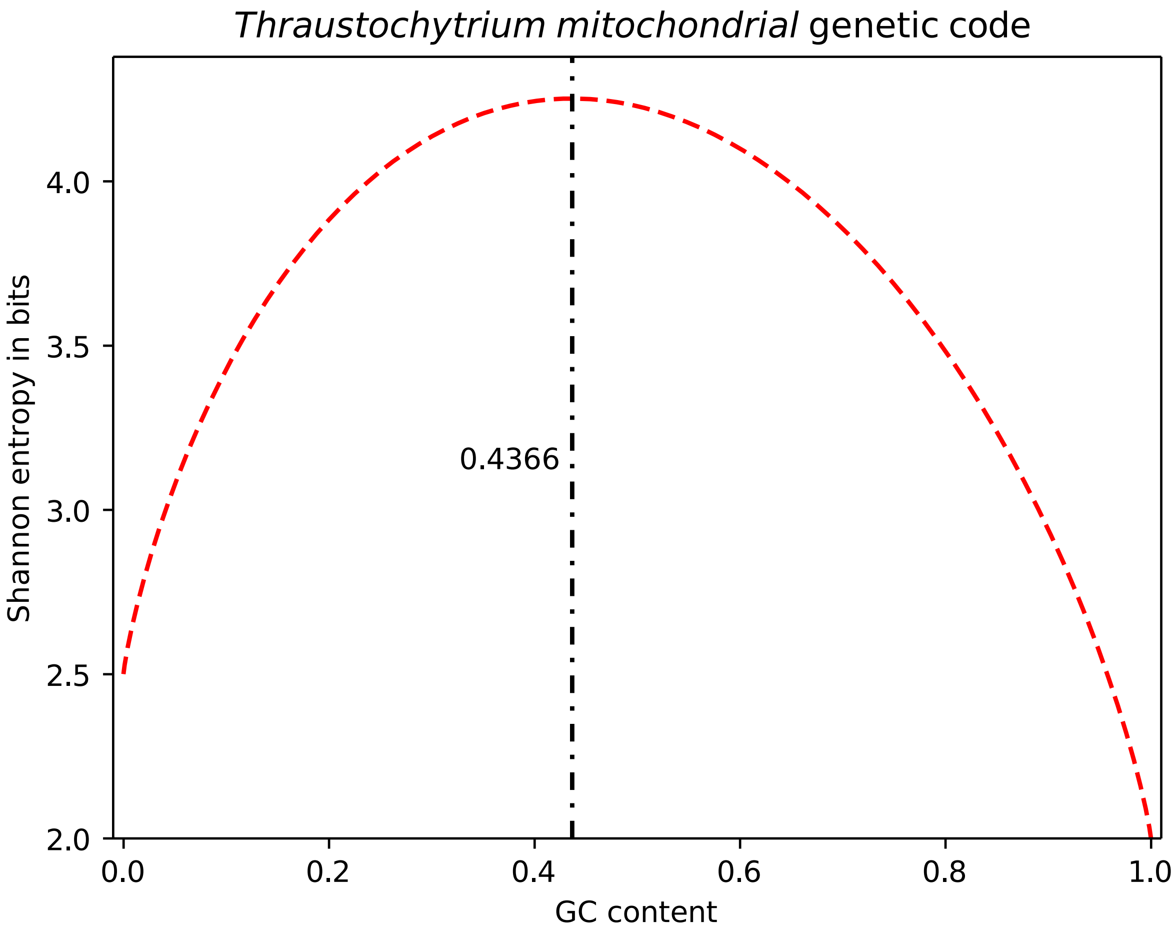

Figure S63. Entropy of the *Thraustochytrium mitochondrial* genetic code for its given codon assignments and GC contents between 0 % and 100 % as calculated by Shannon's entropy equation. The dash-dotted line indicates the GC content (43.66 %) at which this code reaches its entropy maximum (4.25 bits).

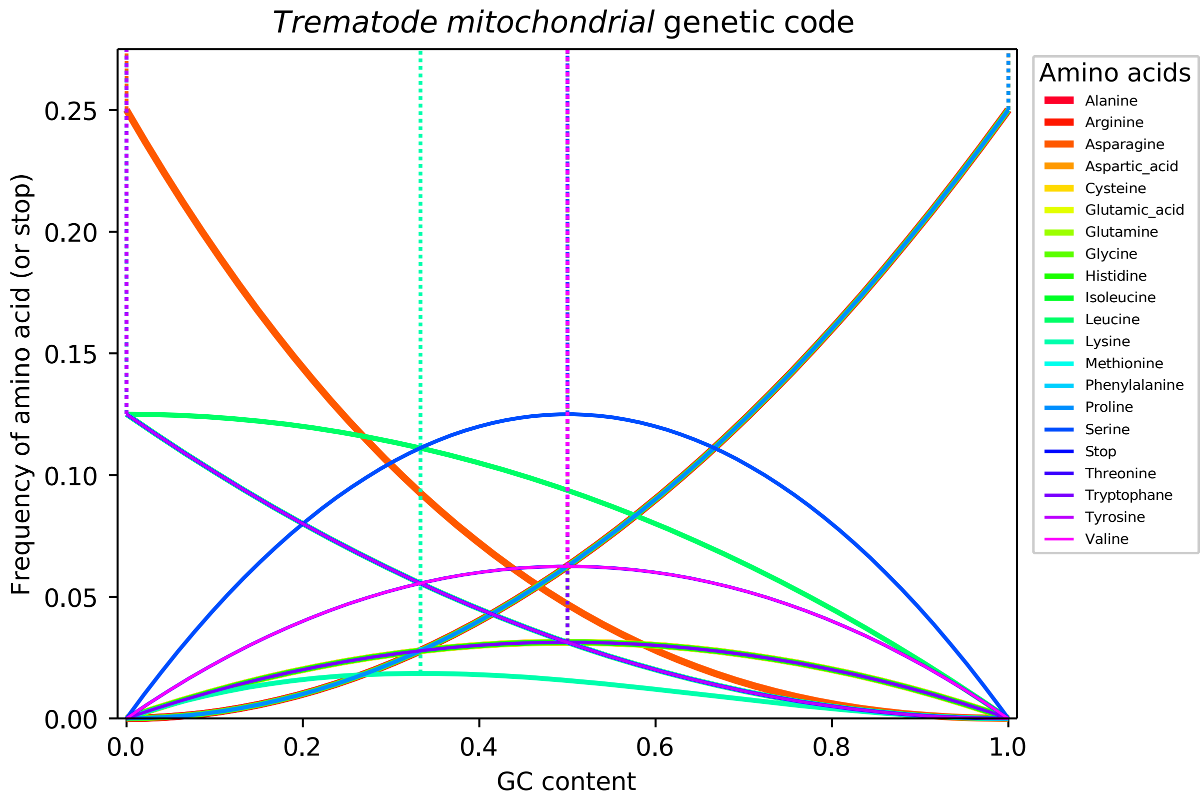

Figure S64. Frequencies of all amino acids (including stop) as encoded by the *trematode mitochondrial* genetic code in random sequences as a function of GC content between 0 % and 100 %. The dashed lines mark the maximum achieved frequency for each amino acid (including stop).

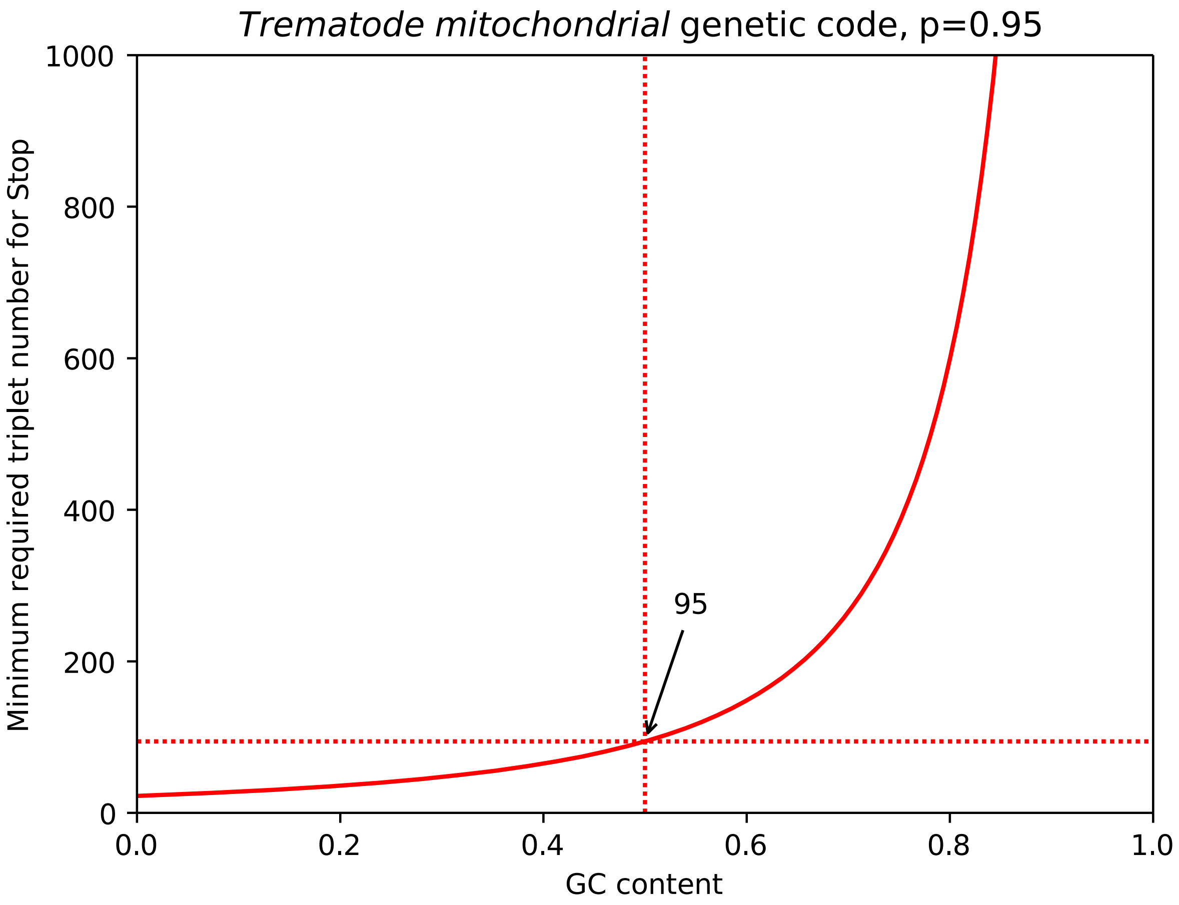

Figure S65. Number of triplets in a random sequence so as to contain at least one stop codon with a probability of 95 % using the *trematode mitochondrial* genetic code as a function of GC content. The horizontal and vertical dashed lines indicate the number of triplets for a GC content of 50 % (95 triplets).

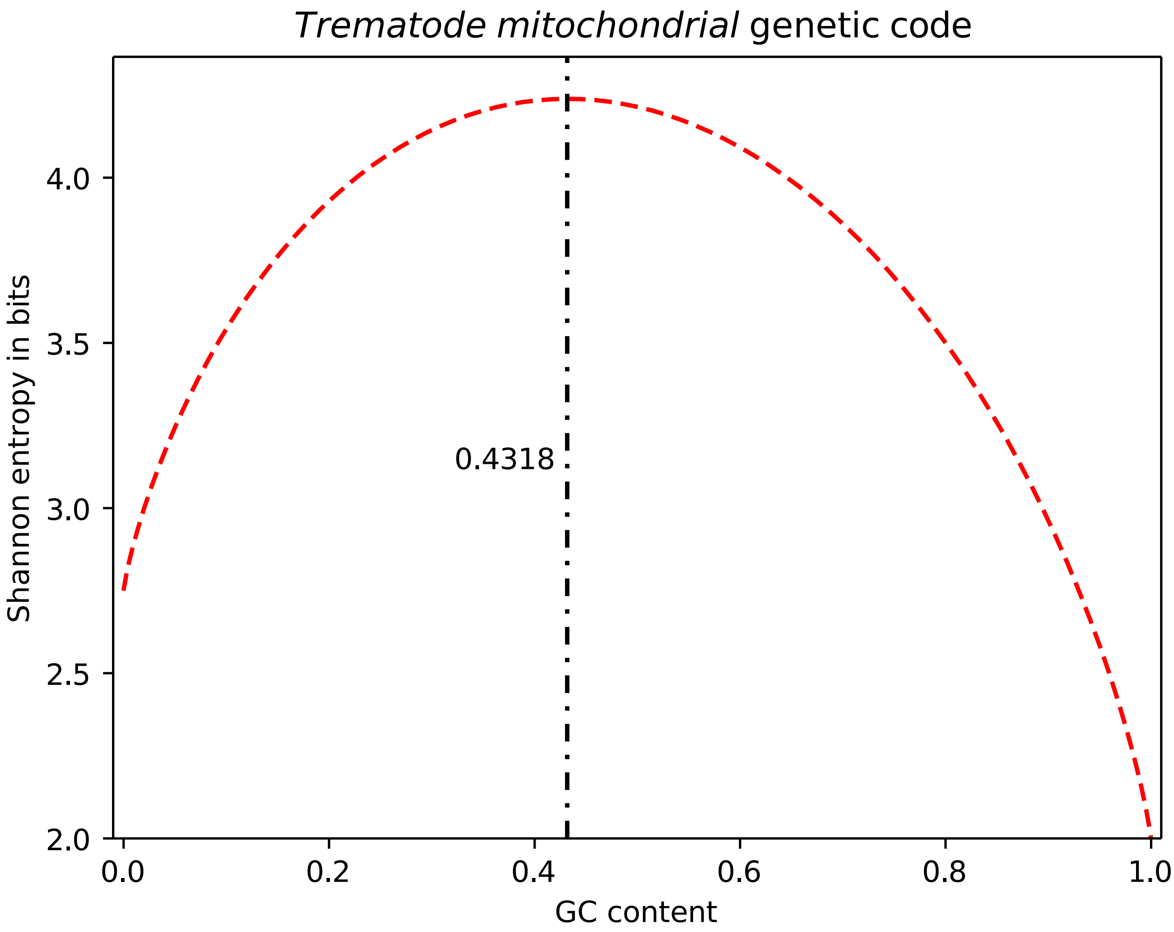

Figure S66. Entropy of the *trematode mitochondrial* genetic code for its given codon assignments and GC contents between 0 % and 100 % as calculated by Shannon's entropy equation. The dash-dotted line indicates the GC content (43.18 %) at which this code reaches its entropy maximum (4.24 bits).

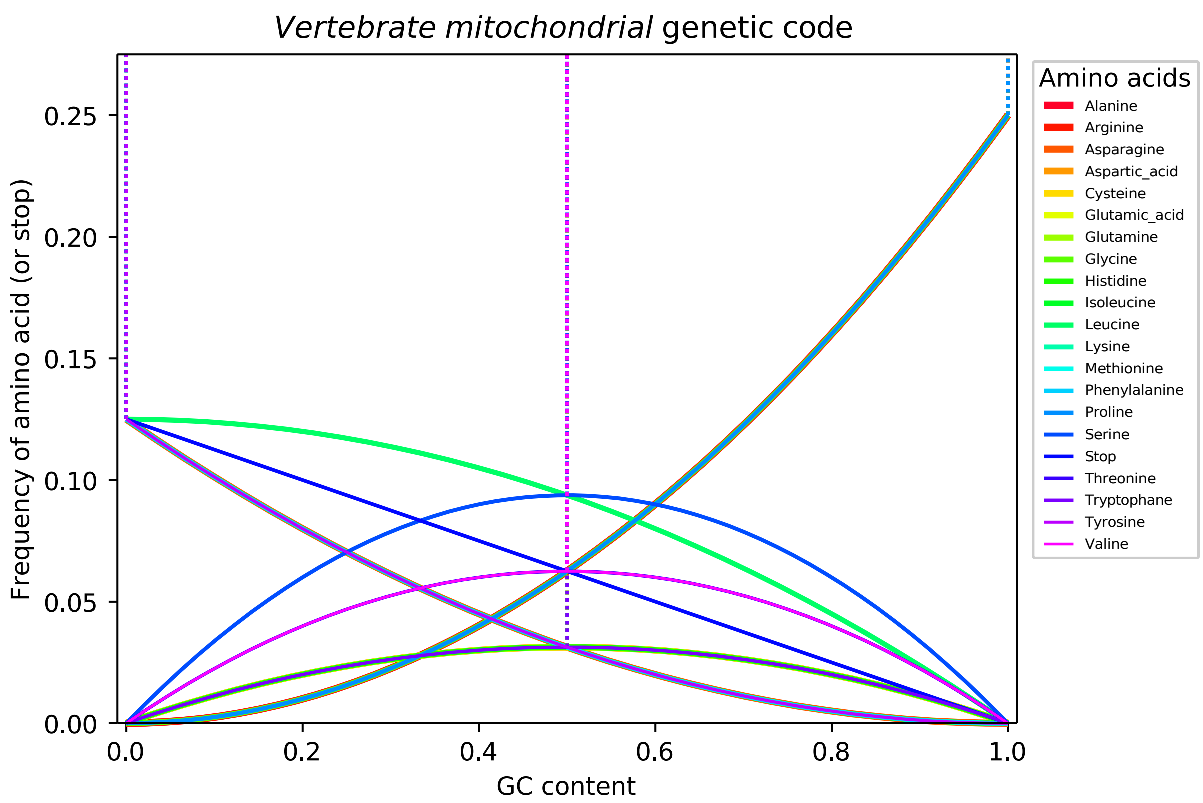

Figure S67. Frequencies of all amino acids (including stop) as encoded by the *vertebrate mitochondrial* genetic code in random sequences as a function of GC content between 0 % and 100 %. The dashed lines mark the maximum achieved frequency for each amino acid (including stop).

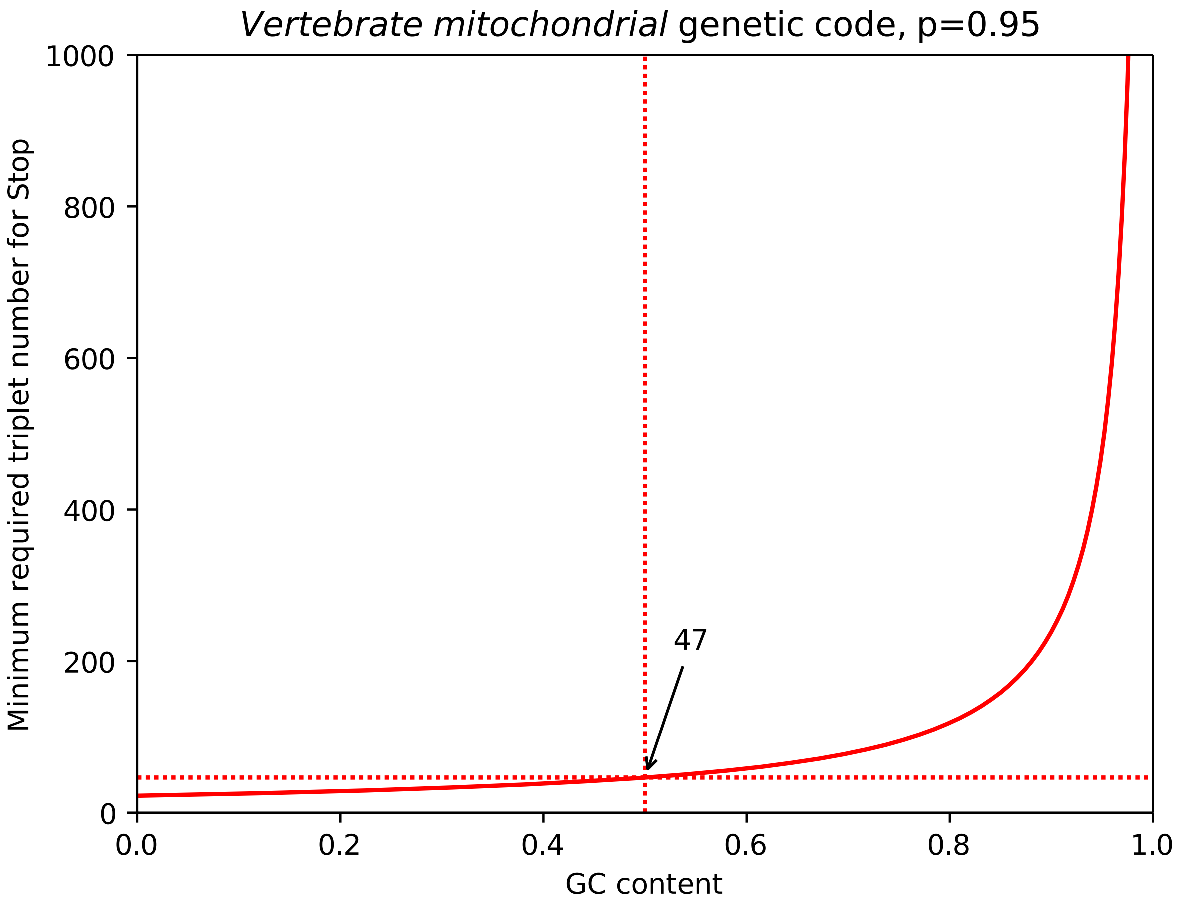

Figure S68. Number of triplets in a random sequence so as to contain at least one stop codon with a probability of 95 % using the *vertebrate mitochondrial* genetic code as a function of GC content. The horizontal and vertical dashed lines indicate the number of triplets for a GC content of 50 % (47 triplets).

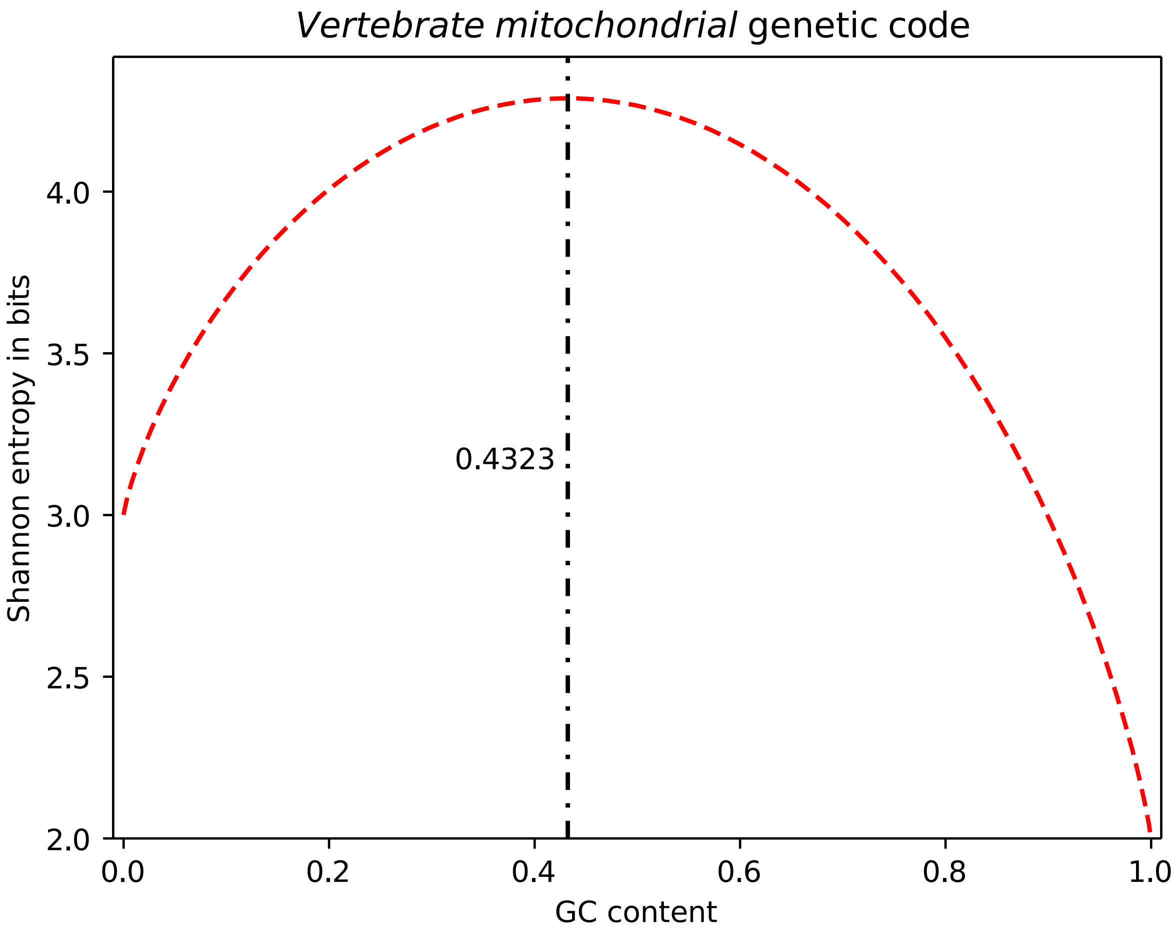

Figure S69. Entropy of the *vertebrate mitochondrial* genetic code for its given codon assignments and GC contents between 0 % and 100 % as calculated by Shannon's entropy equation. The dash-dotted line indicates the GC content (43.23 %) at which this code reaches its entropy maximum (4.29 bits).

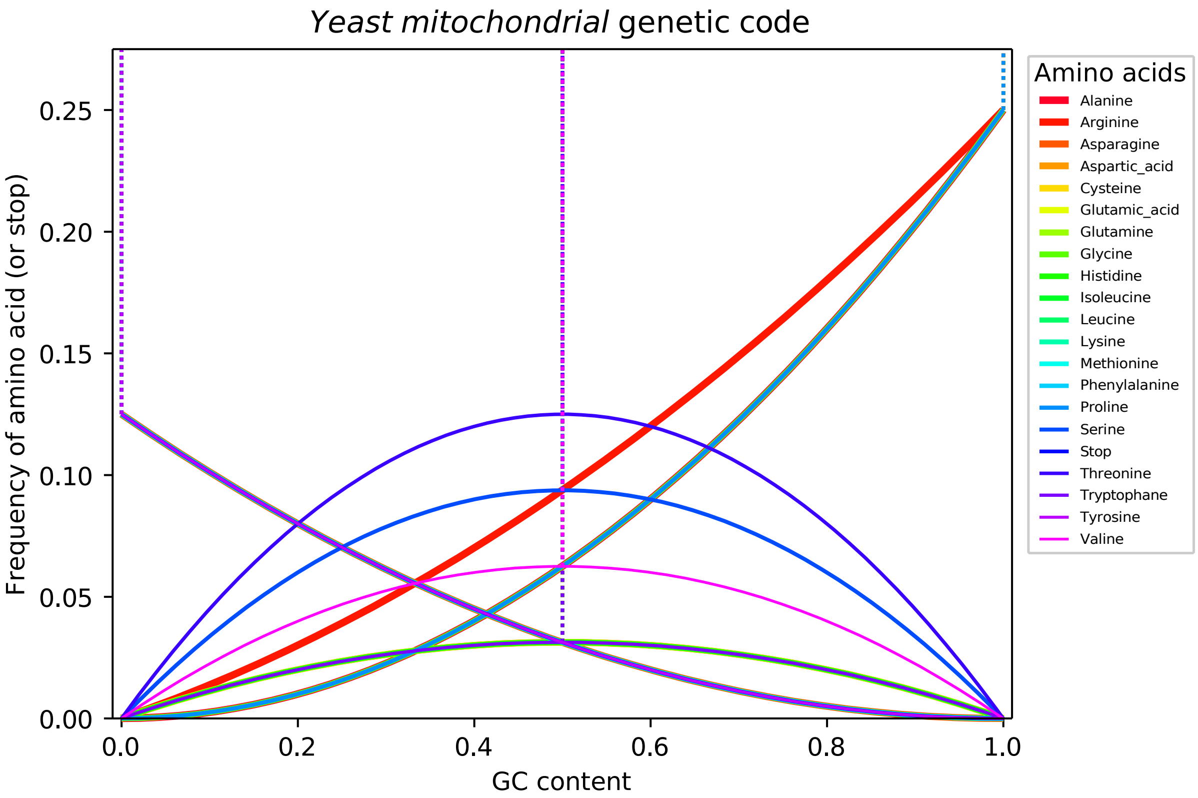

Figure S70. Frequencies of all amino acids (including stop) as encoded by the *yeast mitochondrial* genetic code in random sequences as a function of GC content between 0 % and 100 %. The dashed lines mark the maximum achieved frequency for each amino acid (including stop).

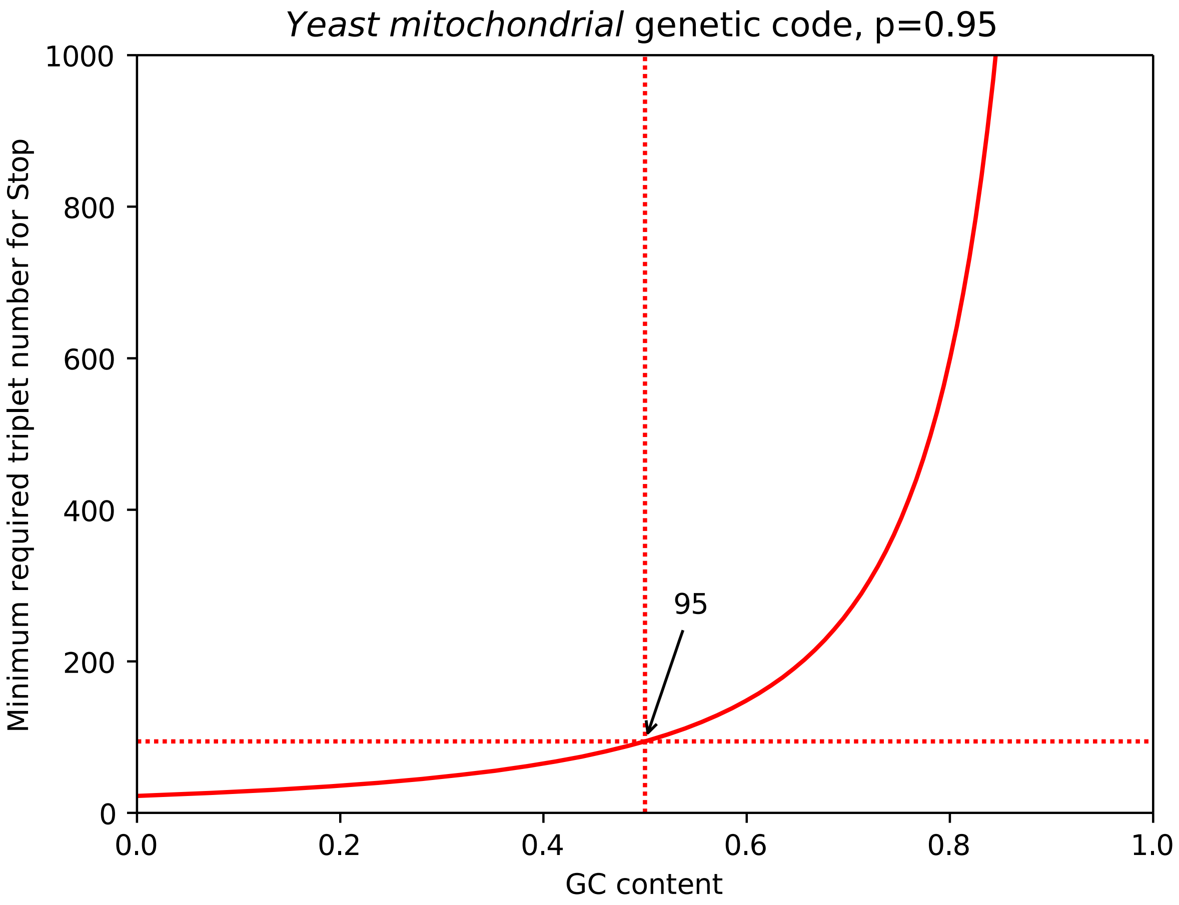

Figure S71. Number of triplets in a random sequence so as to contain at least one stop codon with a probability of 95 % using the *yeast mitochondrial* genetic code as a function of GC content. The horizontal and vertical dashed lines indicate the number of triplets for a GC content of 50 % (95 triplets).

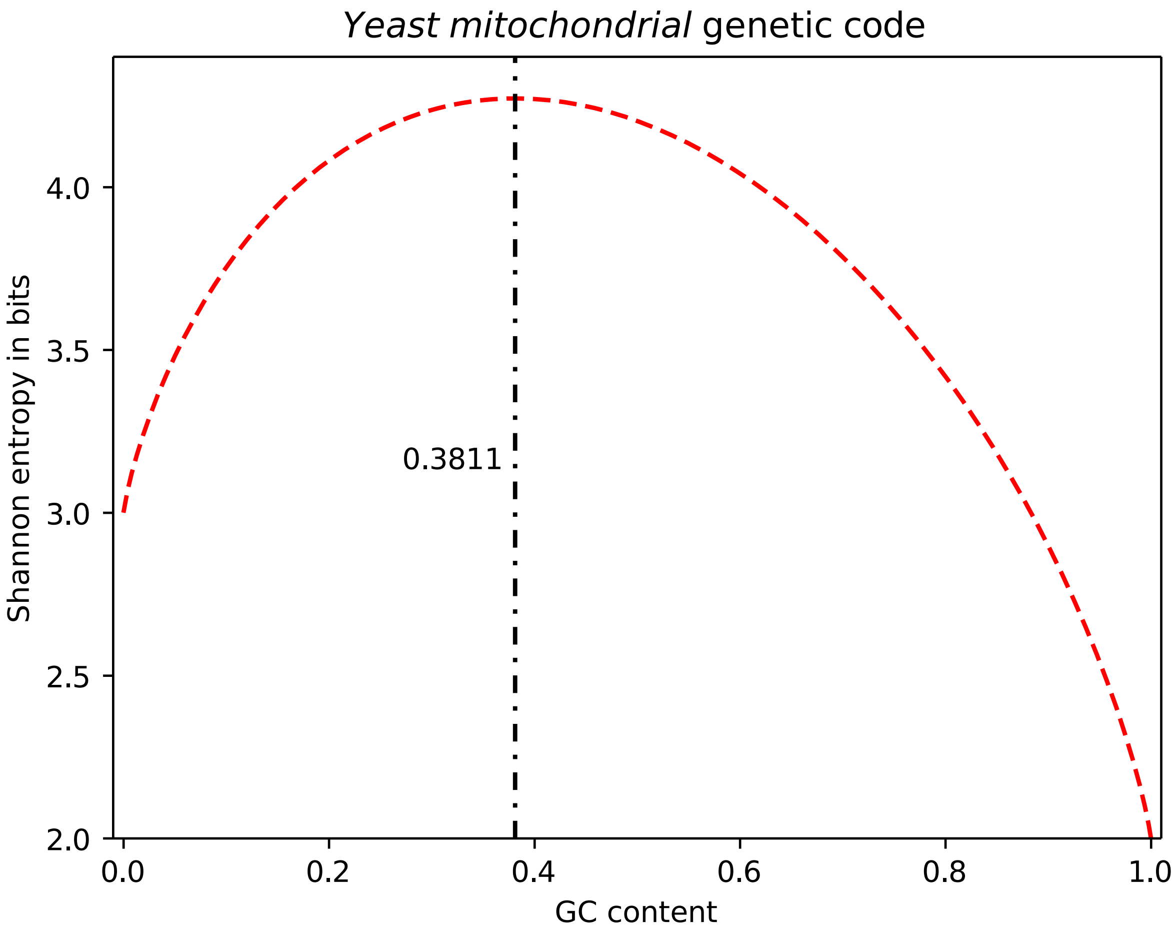

Figure S72. Entropy of the *yeast mitochondrial* genetic code for its given codon assignments and GC contents between 0 % and 100 % as calculated by Shannon's entropy equation. The dash-dotted line indicates the GC content (38.11 %) at which the SGCode reaches its entropy maximum (4.27 bits).
